# Laboratory x-ray micro-computed tomography: a user guideline for biological samples

**DOI:** 10.1093/gigascience/gix027

**Published:** 2017-04-17

**Authors:** Anton du Plessis, Chris Broeckhoven, Anina Guelpa, Stephan Gerhard le Roux

**Affiliations:** 1 CT Scanner Facility, Central Analytical Facilities, Stellenbosch University, Stellenbosch, 7602, South Africa; 2 Physics Department, Stellenbosch University, Stellenbosch, 7602, South Africa; 3 Department of Botany and Zoology, Stellenbosch University, Stellenbosch, 7602, South Africa.

**Keywords:** 3D imaging, micro-computed tomography, nano-computed tomography, non-destructive analysis, x-ray tomography

## Abstract

Laboratory x-ray micro–computed tomography (micro-CT) is a fast-growing method in scientific research applications that allows for non-destructive imaging of morphological structures. This paper provides an easily operated “how to” guide for new potential users and describes the various steps required for successful planning of research projects that involve micro-CT. Background information on micro-CT is provided, followed by relevant setup, scanning, reconstructing, and visualization methods and considerations. Throughout the guide, a Jackson's chameleon specimen, which was scanned at different settings, is used as an interactive example. The ultimate aim of this paper is make new users familiar with the concepts and applications of micro-CT in an attempt to promote its use in future scientific studies.

## Introduction

In recent years, substantial effort has been made to try and improve current techniques for investigating the morphology of biological samples in a non-destructive manner. One of these techniques is computerized axial tomography (CAT) or computed tomography (CT), a method widely used for non-invasive imaging of the anatomy of the human body [1]. Computed or computerized axial tomography involves the recording of two-dimensional (2D) x-ray images from various angles around an object, followed by a digital three-dimensional (3D) reconstruction. The resultant 3D-rendered volume not only allows for the multidirectional examination of an area of interest (e.g., organ), but also permits dimensional, volumetric, or other more advanced measurements to be made [2, 3].

Industrial x-ray computed tomography is a specialized form of CT scanning meant specifically for non-medical applications (hence the term “industrial”) and frequently involves resolutions in the micrometer (μm) range. The method is therefore termed micro–computed tomography (micro-CT) and in the case of sub-micron resolution, such methods are termed nano-CT or sometimes x-ray microscopy as the resolution is similar to optical microscopes. Industrial CT differs from medical CT in three important ways: (i) due to its medical application, the x-ray source and detector move around a stationary sample in medical CT, whereas in industrial CT, the x-ray source and detector are fixed around a rotating sample. This rotating sample design facilitates image resolution adjustment (e.g., higher image resolution for smaller samples). (ii) Industrial CT is more flexible than medical CT with regards to voltage and current modification, which allows for the setup to be modified to suit a range of materials (e.g., higher voltage for dense materials). (iii) The image resolution of industrial CT scanners is often higher than that of medical CT scanners. Resolutions of industrial CT scanners are generally in the range of 5–150 μm, compared to medical CT scanners, which have best resolutions of 70 μm. In contrast, most nano-CT scanners have resolutions as low as to 0.5 μm. However, it is important to note that medical micro-CT scanners optimized for scanning small live animals are available and can obtain similar resolutions as industrial CT scanners.

Industrial CT has numerous applications and is useful in any scientific field where non-destructive analysis is warranted. The versatility of this technique is shown in the number of reviews that have been published recently in areas as diverse as food sciences [4], the geosciences [5], materials sciences [6, 7], and biological sciences [8]. In biological sciences, industrial CT has gained popularity in recent years due to its application in taxonomy [9], paleobiology [10], and evolutionary and ecological biology [11]. The reason that micro-CT scans confer a strong advantage over physical specimens is threefold: (i) measurements are not limited to the external anatomy, (ii) measurements can be obtained at high precision, and (iii) the need to borrow fragile and often valuable museum specimens is eliminated [12]. Replacing access to physical specimens with open access 3D stereotypes that contain morphological and anatomical information of comparable accuracy to that of physical specimens can significantly speed up the documentation of biodiversity (i.e., cybertaxonomy) [13] and facilitate high-power ecological and evolutionary research [12]. In addition, Broeckhoven et al. [14] have recently proposed a protocol that makes use of industrial CT to obtain high-resolution images of the internal anatomy of live reptiles and amphibians without the need to sacrifice study organisms.

Despite its numerous applications and capabilities, the use of industrial CT has not reached its full potential as researchers in the biological sciences are often unfamiliar with the technique and its process, which includes sample preparation, the scanning process itself, and 3D reconstruction. Lack of knowledge can result in poor scan quality and/or inability to extract adequate information for the required research purpose or question. Here, we provide guidelines that can be referred to, not only by new users with a general biological background, but also by CT operators who are unfamiliar with biological specimens. A multi-scale investigation of the Jackson's chameleon (*Trioceros jacksonii*) is used as an interactive study aid throughout the guideline. Ultimately, our aim is to improve the efficiency of micro-CT facilities and biological research through an improved understanding of the capabilities and limitations of the technique.

## Background to Computed Tomography

**Figure 1:**
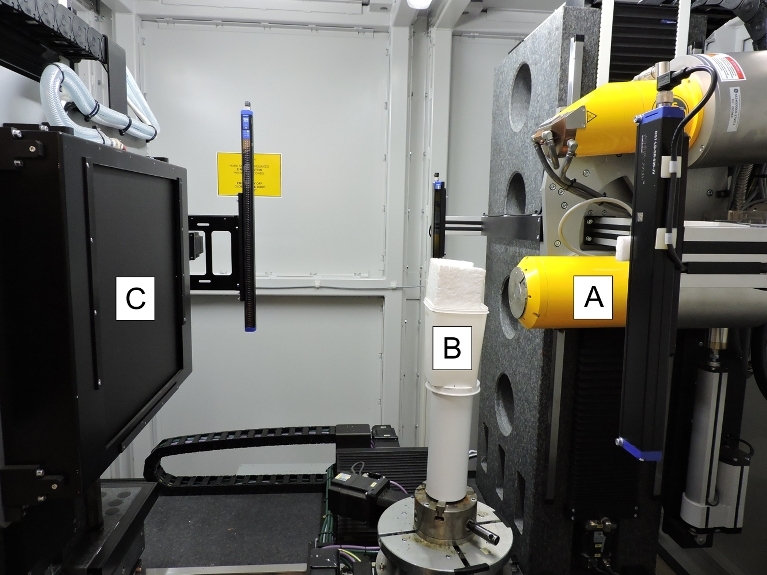
Photograph of the micro-CT scanner used during the study showing the fundamental components of the setup. A typical micro-CT scanner consists of an x-ray tube **(A)** that emits x-rays, which pass through a sample **(B)** before being recorded by an x-ray detector **(C)**.

Micro-CT makes use of an x-ray source and detector to obtain 2D images of a sample that, in turn, can be combined to create a 3D reconstruction [15]. The fundamental components of any micro-CT instrument are (i) penetrating ionizing radiation, (ii) a sample manipulator, and (iii) a detector (Fig. 1) [16]. The basic principle of micro-CT is described in Kak and Slaney [17]. X-rays are generated by a micro-focus x-ray tube, which uses a beam of electrons accelerated by a voltage of up to 240 kV (or more in a vacuum tube), and are focused onto a tungsten or similar metal target. The interaction between the fast-moving electrons and the metal target is responsible for creating x-rays. The x-rays are then directed through and around a sample before being collected on a 2D x-ray detector in the form of a “shadow image,” also called a projection image or radiograph [3]. In industrial CT, the sample manipulator (or rotation table) positions the sample in the path of the radiation beam and rotates it through a specific angle (usually 180° or 360°). The detector converts the attenuated radiation, which passes through the sample along a straight line into the 2D digital images, consisting of thousands of pixels. In this way, many hundreds or thousands of 2D projection images are recorded during the scan process. After scanning, these images are used to reconstruct a 3D data set by making use of filtered back-projection algorithms [18]. Effectively, every volumetric pixel (or voxel) is imaged (by 2D projections) from many angles, and the sum of its view from every angle produces a representation of the actual x-ray density and hence brightness of that voxel [3]. Following reconstruction, a variety of software tools can be used for data visualization and analysis. These steps are all described below with a discussion of practical considerations (Fig. 1).

## Computed Tomography Procedure

The micro-CT procedure includes various steps such as (i) sample preparation and mounting, (ii) scanner setup and parameter selection, (iii) scanning procedure, (iv) image reconstruction, and (v) image visualization. We refrain from explaining the image processing and analysis steps as this is highly dependent on the software used, but researchers can make use of the program developer's user manuals for this information. The setup considerations are explained here, together with three general guidelines (Guidelines I to III) that can be used to aid the scanning process. The entire micro-CT procedure will then be explained, where applicable, using a Jackson's chameleon (*Trioceros jacksonii*) from the Ellerman Collection at Stellenbosch University (voucher specimen deposited under number USEC/H-2927) as an example. No ethics or institutional approval was required as the sample concerned was an ethanol-preserved specimen. The sample was scanned using a Phoenix V|Tome|X L240 (General Electric Sensing and Inspection Technologies/Phoenix X-ray, Wunstorff, Germany) micro-CT system, as well as a Phoenix nanotom S (General Electric Sensing and Inspection Technologies/Phoenix X-ray, Wunstorff, Germany) nano-CT system, both located at the CT Scanner Facility of the Central Analytical Facility (CAF), Stellenbosch University, South Africa [19]. Full data sets that accompany the descriptive analysis are provided as supplementary information [20]. These data sets can be used as an interactive study aid to obtain a better understanding of viewing and handling typical 3D data sets resulting from the proposed procedure.

### Sample preparation and mounting

**Figure 2:**
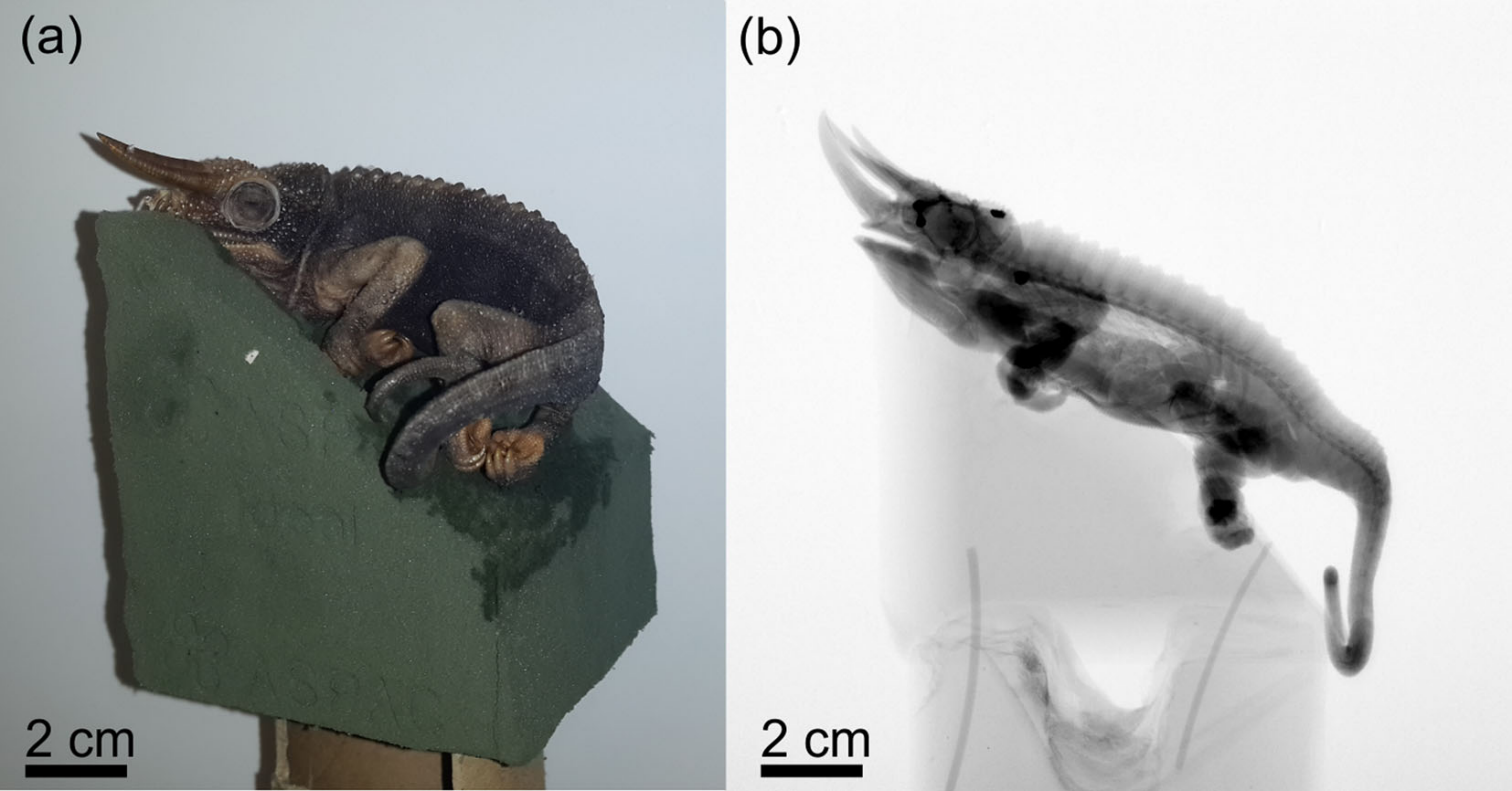
Mounting of a Jackson's chameleon. Florist foam mounting material forms the basis onto which the sample is placed **(A)**. A 2D x-ray projection image shows the very low density of the mounting material **(B)**.

Micro-CT requires very little, if any, sample preparation, and a sample can usually be scanned exactly as provided. Because of the rotating sample design of industrial CT scanners, it is important to load the sample correctly to avoid movement during scanning. Sample mounting involves the use of a low-density materials (e.g., cardboard tubes, plastic bottles, or glass rods) that hold the sample in place on a rotation stage but separate the sample from the dense rotation stage hardware. We suggest that samples are loaded at a slight angle to ensure that parallel surfaces to the x-ray beam are minimized (Fig. 2). This is because parallel surfaces are not penetrated properly by the x-ray beam and can lead to image artifacts and lack of detail in the data set, particularly in the plane of the flat surface parallel to the beam.

As mentioned previously, the most important factor is to avoid movement of the sample during scanning. For example, if the sample is not properly secured in its holder, sample movement will inevitably result in a blurred 3D image that might not be suitable for analysis. Likewise, dehydration of a preserved or wet sample can cause shrinking and might result in a blurred image; this is particularly relevant during longer scan times. Various approaches can be used to overcome these problems, the most convenient being to dry the sample before scanning. However, as this technique is rather invasive, it is unsuitable for valuable or delicate samples, such as fragile museum specimens, and should be avoided unless the samples are not being reused. A more suitable method is to wrap the sample in a wet cloth (i.e., drenched in water, ethanol, formalin, or isopropanol), thereby keeping the sample moist during the scanning procedure. Another option is to scan samples inside liquid-filled tubes. However, care must be taken that the sample is not held in place by the edges of the container because these edges will not be separable from the sample during the image processing steps. It should be noted that some samples are too small or delicate to be removed or are prohibited from being removed from their containers and might need to be scanned *in situ*. In these cases, staining should be considered to increase the contrast of the specimen compared to that of the surrounding medium. For further information on soft tissue scanning and staining methods to enhance contrast, see the studies by Mizutani and Suziki [8], Metscher's [21], and Pauwel et al. [22]. The choice of mounting method will often be determined by the museum to which the sample belongs. In this case, the museum curators should carefully weigh up the advantages and disadvantages of the abovementioned options to ensure that researchers can easily and rapidly obtain scans with high image quality.

The mounting procedure for nano-CT scanning is similar to that of micro-CT scanning of very small samples. The sample is mounted on top of a small glass rod and secured with double-sided tape or glue, or it can be placed inside a small cube of foam, fitted with a small cavity, or slit and attached to the glass rod. A plastic film (e.g., Parafilm^®^) can be used to cover soft tissue or wet samples to avoid dehydration (Fig. 2).

### Scanner setup and parameters

#### Sample size versus resolution

**Figure 3:**
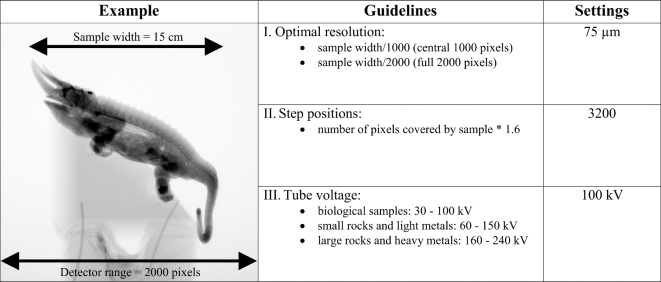
Summary of Guidelines I–III showing how the optimal scanning settings for our Jackson's chameleon example were determined. Note that these guidelines are based on a 2000 pixel detector. See text for further information.

Careful selection of resolution is the first major factor affecting a micro-CT scan. A useful guideline (Guideline I, see Fig. 3) when estimating the best possible resolution for a sample of known dimensions is as follows:

- The optimal resolution is a factor 1000 smaller than the width of the sample. For instance, a sample with a width of 100 mm has an optimal resolution of approximately 100 μm.

The above guideline is based on the standard practice of using only the central 1000 of 2000 available pixels of the detector to minimize possible artifacts from the edges. This is due to two reasons: first, the cone beam has reduced intensity near the edges, and second, the cone beam geometry results in non-ideal reconstruction away from the central slice. For both these reasons, it is suggested to use the middle of the detector to minimize the artifacts and reduce contrast near the edge. While most detectors have 2000 pixels, some models have more to allow for improved magnification for the same sample size. This, however, might introduce other problems, including an increase in data set size and prolonged reconstruction times. It must be noted that it is theoretically possible to use all 2000 available pixels in the above example, resulting in a resolution of 50 μm. However, besides the risk of artifacts from the edge regions, it can be challenging to mount a sample perfectly central on the rotation axis to avoid movement out of the field of view during rotation.

#### Resolution, voxel size, and x-ray spot size

The voxel size of a micro-CT image is dependent on the magnification and object size as described above. This is related to the distance of the sample from the x-ray source and the detector [4]. Voxel size and spatial resolution are two concepts that are often confused since the voxel size is the size of a pixel in 3D space, i.e., the width of one volumetric pixel (isotropic in three dimensions). This value does not consider the actual spatial resolution capability of the scan system. For example, if the x-ray spot size (focused x-ray spot from the source) becomes larger than the chosen voxel size, the spatial resolution of the system becomes poorer. That means that fewer details are detectable, despite a good voxel size, due to the actual resolution being non-optimal. Since most commercial systems limit the size of the x-ray spot to the required voxel size (or provide the user an indication of this), the actual and voxel resolution are usually the same, but this is not regularly tested or reported. It is possible to use resolution standards (such as calibrated-thickness metal wires) to confirm spatial resolution, and some reference standards exist, although a generally accepted standard for industrial CT systems does not yet exist. It is therefore possible that the amount of detail that is detectable in a scan can vary considerably from system to system, or even between different scans from the same type of system. These quality differences are either due to improper settings that may result in large x-ray spot sizes or to an improper choice of other scan parameters. The sole way of testing the scan quality is to image a small feature of known dimensions and ensure the feature is visible in the CT slice image.

#### Scan time, number of images, and rotational options

The major consideration for scan time is the acquisition time of single projection images, which can vary from system to system due to detector sensitivity and dynamic range differences, x-ray tube brightness differences, and differences in physical distance form source to detector [3]. A typical image acquisition time in a walk-in cabinet system with a 16-bit flat-panel detector is 500 ms per image, while some benchtop systems may have image acquisition times from a few hundred ms to up to several seconds per image. All systems have variable image acquisition times, and therefore scan times can vary considerably. To obtain the highest possible scan quality, the full dynamic range of the detector should be explored. By doing so, the image contrast is maximized by raising the image acquisition time up to near saturation of the detector for a particular x-ray setting. If the image acquisition time is too low, the resulting contrast will be poor, with grainy images in extreme cases.

Some scanners involve continuous scanning (i.e., continuous rotation and image acquisition without steps), but for simplicity discussion here is limited to stepwise rotation. At each step position, one or more images can be acquired and averaged to provide an improved image quality compared to a single image per position. While the averaging method reduces noise and consequently improves image quality, its effect highly depends on the inherent noise of the detector used. For samples that might experience small vibrational movements during rotational movement (e.g., leaves or hairs), it is advisable to use the skip function (if available) because it ignores the first image acquired at each new step position (during which time the sample stabilizes). Since this vibration is due to the stepwise process, an alternative approach would be to use continuous scanning because it also reduces vibration. In this case, however, averaging is not possible.

The number of step positions required depends on the sample size relative to the magnification. Therefore, the higher the magnification and hence the number of pixels used on the detector, the larger the number of images required for a good reconstruction. A useful guideline in this regard (Guideline II, see Fig. 3) is that

- the number of pixels covered by the sample on the detector in width (pixels) multiplied by 1.6 equals the number of projection step positions required. Consequently, up to a maximum of 3200 step positions are used for a typical 2000 pixel–wide detector.

#### Scanner parameters

Voltage: x-ray voltage highly depends on the type and material composition of the sample. The most optimal material discrimination is usually obtained by using lower voltages. However, the x-ray penetration value (i.e., the percentage of detector counts around and through the sample) might be too low in the case of dense material, thereby causing noise and artifacts. Beam hardening represents the most common CT artifact, causing noise and artifacts (see the “Scan quality problems and artifacts” section for more). Beam hardening occurs when the x-ray beam, which comprises a range of x-ray energies, encounters differences in absorption from different angles and along different paths through the object, either due to a very dense object itself or due to dense parts of an object. Different x-ray paths result in varying absorption of the easily absorbed low-energy x-rays, in turn resulting in either “cupping” artifacts in dense objects (brighter regions around the edges of the material) or streaky artifacts in dense parts of a larger object (especially for very dense parts, such as metal tags).

Filtration: two applications of filters exist: (i) the filter is placed between the x-ray source and the sample or (ii) the filter is placed between the x-ray detector and the sample. The first type of filtration, called beam filtering, is useful when the voltage is increased and a beam filter is added to pre-compensate for expected beam hardening. The filter effectively reduces the polychromaticity of the beam, thereby preventing streaky artifacts. Frequently used beam filters include 0.1 to 2 mm of copper and 0.5 to 1.5 mm of tin, combinations of both, and aluminum. The second type of filtration, detector filtration, can also be used to reduce noise if, due to the density of the object, secondary x-ray emission is produced or scattering is present. This may happen when a dense material strongly absorbs x-rays and re-emits lower-energy x-rays by fluorescence or when a large amount of scattering is present from nanostructured samples, causing x-ray scattering. In both cases, using a filter after the sample and before the detector shields the detector from low-energy x-ray emission and scattering, limiting noise.

Guideline III is presented for the calculation of the scanner voltage and determining adequate penetration values.

- The following x-ray tube voltages can be used as a starting point: biological samples: 30 to 100 kV; small rocks and light metals: 60 to 150 kV; large rocks and heavy metals: 160 to 240 kV or more; and in general: small samples require low voltage.
- A typical setup method to find the best settings for a particular sample type is to rotate the sample until its 2D x-ray projection image shows the darkest region (its longest or densest axis). The user can then calculate the sample's minimum penetration ratio compared to the background x-ray intensity using the gray value counts measured in the x-ray image. Penetration values from 10% to 90% should result in good scan quality. If the penetration value is less than 10%, an increased voltage or current is required, whereas if it is above 90% the voltage or current should be lowered.
- If the x-ray detector becomes saturated as a result of (ii), beam filters can be applied to prevent saturation while still increasing the penetration value. By making use of a beam filter, a higher voltage or current can be obtained with a reduction in low-energy x-rays, such that the detector does not yet saturate (Fig. 3).

### Scanning procedure

Prior to scanning, it is important that the background is normalized. Background normalization is achieved by removing the sample and using the x-ray beam at the chosen settings to correct for all intensity variations across the detector (i.e., the x-ray beam being more intense in the middle of the detector compared to the edges of the detector). This normalization procedure can be conducted prior to each scan but in practice is only required if x-ray or acquisition settings change or after a long period of scanner inactivity. In addition, it is necessary to run a beam centering prior to scanning to ensure correct focusing of the electrons, thereby ensuring the smallest spot and highest emission. In most commercial systems, however, this is an automated process. Once the sample is loaded and settings chosen, images can be acquired. The scanning itself is done automatically with no user interaction. Frequent supervision is advisable as several errors may occur during this process, including x-ray source instability (requiring a warm-up) or filament burn (requiring replacement). It is important to note that addressing these issues can take a considerable amount of time and this should be taken into account during data collection planning.

Although our proposed scanner settings are aimed at acquiring high-quality images, it is possible to obtain a shorter scanning duration. This can be achieved by using fewer images, eliminating averaging, and reducing exposure times. Fast scans (e.g., 5–15 minutes) might not be optimal but can be sufficient in some cases, e.g., when trying to identify a relatively large feature or when simple measurements have to be taken. Alternatively, they are also used as an exploratory method to find a region of interest prior to commencing a long, higher-quality scan.

### Image reconstruction

After all 2D image projections are obtained, a 3D volume can be constructed. The reconstruction process involves the mapping of each voxel by using projection image representations of a particular voxel from many angles. This mapping is done by a Feldkamp filtered back-projection algorithm [23]. Commercial micro-CT systems have built-in reconstruction software packages that might differ in settings but are all based on the same algorithms. For example, Volume Graphics [24] is a standalone software package mainly used for 3D image analysis but also offers a module for reconstruction. Another commercial standalone software for reconstruction is Octopus Reconstruction from Inside Matters [25].

Reconstruction software involves a series of settings, which might affect the quality of the obtained 3D data. These options will be described in general here, though reconstruction software packages might differ in their availability of the features offered. First, the field of view can be cropped to make the total reconstructed volume smaller. This helps by reducing data volumes as well as the duration of the reconstruction since less memory is required. This is especially helpful when time or computational power is limited. Second, the type of output file can be chosen, which is usually selected as 16-bit. Here, it is also possible to select 8-bit if storage space or memory is limited. Third, the exact location of the rotation axis in each projection image is found by making use of an automated algorithm that finds the central pixels in all 2D x-ray images. The use of the exact rotation axis in the back-projection algorithm improves the quality of the reconstruction and is especially important at higher resolutions. This process can also be coupled with a refinement process, correcting for small movement or any shift of the sample and improving the edge clarity in the reconstructed data set. Next, beam hardening correction is considered. Beam hardening corrects much of the generally occurring “cupping” effect in samples where the edges seem brighter than the middle of the scan. Another option called clamping disregards a certain percentage of pixels that are “outliers” in terms of strong or weak absorption compared to the rest of the data and effectively improves the gray value contrast in the images. Clamping can be very useful when a small quantity of bright dense phases that are not of interest is present. The percentage of pixels that are clamped and the clamping direction (lowest or highest gray values only, or both) can be set. Furthermore, it may also be possible to make use of special settings to select the background detector counts in each image and normalize this across the series of images, which is useful when scattering is present, resulting in brighter or darker projection images from different angles. It is possible to use special algorithms to remove ring artifacts by disregarding “dead” pixels from the 2D projection images. Ring artifacts, especially near the center of rotation, are also removed by making use of a detector shift process whereby the detector shifts horizontally between step positions, which are corrected in the reconstruction process, resulting in a smoothing of the rotational center artifact. It is clear that various options exist for the reconstruction of a data set, thereby making this process an important step that can help the user with obtaining improved image quality. Since the reconstruction process itself can vary significantly, it is suggested that the raw 2D x-ray projection images are retained after completion of the reconstruction process as this will allow the user to improve the reconstruction of the same data in the future.

### Image visualization

Micro-CT data can be visualized in two different ways, either by volume rendering or surface rendering. Volume rendering is typically conducted in a 3D data analysis software package and involves iso-surface views using a user-defined threshold value or a user-defined grayscale gradient for more advanced 3D rendering algorithms. These differ from 3D Computed Aided Design (CAD) software in that they handle full voxel data, i.e., the data exists throughout a 3D voxel grid, not only on surfaces of the object. In other words, CAD software packages use triangulated mesh data of surfaces only (point locations), while full CT data are comprised of data at every point in 3D space (gray value at every point). Therefore, a volumetric data set is significantly larger and requires more intensive computing power, even for simple visualization. Commonly used commercial software available for volume rendering include Volume Graphics VGStudio [24], Amira and Avizo [26], and Simpleware [27], whereas surface rendering software are Blender [28], SolidWorks [29], and Autodesk [30]. Additionally, freeware (or open source) software, which can be used for analysis of CT data in 2D or 3D, include ImageJ [31], MIPAR [32], Blob3D [33], Quant3D [34], and 3dma_rock [35]. For more detailed information regarding software options that allow visualization of micro-CT data, see Walter et al. [36].

### Scan quality problems and artifacts

**Figure 4:**
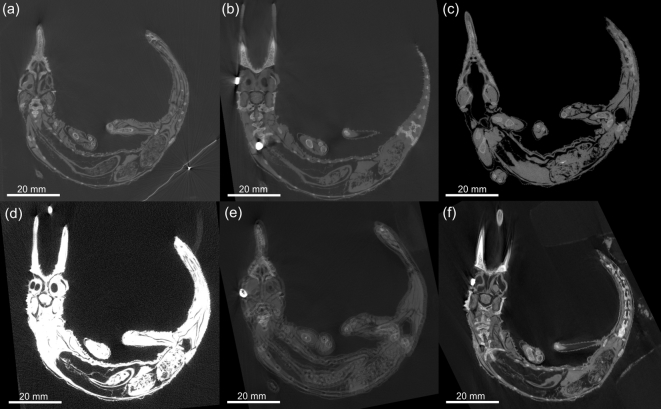
Micro-CT slice images of a Jackson's chameleon illustrating the common artifacts. In **(A)**, a metal tag is included in the scan volume, resulting in streaky artifacts (bottom right in image). In **(B)**, an insufficient voltage was used, thereby creating image artifacts around the dense parts of sample. In **(C)**, the voltage setting was too high, resulting in poor contrast. In **(D)**, poor image quality is caused by reconstruction clamping, which was set too high. In **(E)**, double edges are present due to incorrect offset calculations during reconstruction. In **(F)**, slight blur is present due improper mounting.

The diversity of available scanner options and settings when used incorrectly can be associated with various image quality problems and artifacts, the following examples demonstrating some of the typical problems. Figure 4, A–C, shows micro-CT slice images of the chameleon with metal streak artifacts present, too-low voltage, and too-high voltage, respectively. In the first case, the streak artifacts reduce the image quality of the specimen, while too-low voltage causes brightness variations around dense objects in the image and too-high voltage results in poor contrast between materials. It is not only the scan process but also reconstruction that can affect the image quality as shown in Fig. 4, D–F. Figure 4D has poor contrast, in this case due to incorrect reconstruction setting (clamping). The same effect may occur when a sample is scanned with the metal rotation table in the scan volume. Figure 4E has a double edge due to incorrect reconstruction setting (i.e., offset correction). This double edge can also occur if the sample moves during a scan, though to a lesser degree. Figure 4F illustrates a slight blur on the edges, which is due to sample vibration due to non-rigid mounting of the sample and stepwise rotation, causing the sample to move slightly, more so on the top than the bottom of the sample (Fig. 4).

Beam hardening has been mentioned previously within the context of streak artifacts. However, samples with homogenous material density scanned with an insufficient voltage might also result in a “cupping” effect. This artifact arises when x-rays do not penetrate the sample sufficiently. Other artifacts and unwanted image effects include cone beam artifacts affecting the edges of materials near the edges of the detector, double edges due to tilt axis misalignment relative to beam axis, and blurring due to an unstable rotational axis. For more on this, see relevant publications on CT artifacts by Barrett and Keat [37] and Boas and Fleischmann [38]. Additionally, Table 1 summarizes problematic micro-CT scans as discussed in this paper, providing the causes and possible solutions to the problem (Table 1).

**Table 1:** Summary of the various errors and artifacts discussed throughout this paper, stating the problems, possible causes, and potential solutions, respectively.

Problem	Cause	Solution
Grainy image	Image acquisition time too low	Increase image acquisition time
Streaky artifacts	Differences in absorption from different angles; x-ray penetration is insufficient	Increase voltage
Poor contrast	Too high voltage is used	Reduce voltage
Blurred image	Improper sample mounting; allowing sample to move during scanning	Proper mounting to ensure no movement during scanning
Stitching artifacts/vertical or horizontal line	Reconstruction algorithms when stitching sample is too wide for a single scan	Make sub-sections of sample; use a smaller sample or less magnification
Beam hardening/cupping effect	Insufficient penetration of the sample	Reconstruction: use beam hardening correction option or scan with higher voltage and more beam filters
Small movement or shift (double edge)	Inaccuracy of rotation stage or movement of sample	Reconstruction: do an offset correction or rescan if offset cannot be corrected; reset stages; hardware could be faulty, e.g., tilt axis alignment
The image is very dark on materials of interest, with bright spots in places	Small quantity of bright dense phase is present, but irrelevant	Reconstruction: make use of the clamping option
Scattering	Causes brighter or darker projection images from different angles	Reconstruction: select background detector counts in each image and normalise across the series of images
Ring artifacts	Bright rings are visible in the top slice view	Reconstruction: make use of ring artifact reduction by disregarding “dead” pixels from the projection image (or disregard pixels in the acquisition process)
Central rotation artifact	The center of rotation is visible as a line in a side slice view or a dot with concentric rings from the top view	Make use of detector shift option in acquisition, which smooths out the artifact
Bright ring around outside of scan volume, resulting in poor image quality	In ROI scans where the sample extends over the side of the 2D image	Use special reconstruction algorithm that corrects for this or crop the ROI further in reconstruction
Cone beam artifacts	Affecting the edges of materials near the edges of the detector	Use less magnification to fill fewer pixels on detector

### Example: micro-CT scanning of a three-horned chameleon

The considerations, guidelines (Fig. 3) and options related to micro-CT scanning of biological samples are presented here and can be used as guiding principles when conducting micro-CT scans and analysis. The three-horned chameleon is used as an example and will follow the step-wise guidelines as presented in this paper (data available for inspection in the *GigaScience* database [20]).

**Figure 5:**
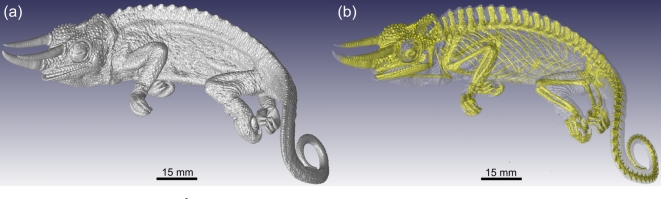
Three-dimensional reconstructions of a Jackson's chameleon illustrating a surface view **(A)** and a semi-transparent view showing the skeleton in yellow **(B)**.

- Sample preparation and mounting: a preserved three-horned chameleon specimen was taken out of its preservation jar and dried out at ambient conditions for a few hours prior to being mounted on florist foam fixed on top of a cardboard tube (Fig. 2A). Although this method might not be ideal for museum specimens (see the “Sample preparation and mounting” section), it was chosen to avoid imaging artifacts associated with movement during dehydration. The densest features of the chameleon can be seen as the darker regions of a digital x-ray projection image of the specimen (Fig. 2B).
- Scanner set-up and parameters: the maximum horizontal width of the sample, when loaded at an angle as shown in Fig. 2, was approximately 150 mm. When positioning the sample such that this width almost covers the full 2000 pixels of the detector, and using Guideline I, the best possible resolution that could be obtained was 75 μm. Following Guideline II, 3200 step positions were used. The sample was loaded at 45° because it provided a slight improvement in the best possible voxel size compared to horizontal or vertical mounting for a single scan volume (vertical or horizontal would be limited to the longest axis of the chameleon sample). It would have been possible to load the sample vertically and scan at a similar resolution, but this would have required multiple scans. Averaging was set to 2, and skipping of the first image at each new position was used. Initially, a typical image acquisition time of 500 ms was set, resulting in a total scan duration of approximately one hour. Tube voltage was set to 100 kV, whereas the beam current was set to 100 μA. No beam filtration was used. This setting showed a good penetration value, but due to relatively low signal values on the detector, the current was increased to 200 μA to obtain approximately 8000 counts, where 10 000 is the saturation level of the detector (Guideline III). In this process, a trade-off between scan time and image quality was found. Higher-quality imaging would have been possible with more averaging, resulting in longer scan times. Higher quality would also have been possible at lower voltage since the penetration values were quite high. When lowering the voltage, the total x-ray emission from the source reduces, which requires a longer image acquisition time to allow the best possible contrast capable with the detector. However, this also increases scan time, and additionally lower voltages can cause unexpected artifacts as explained above.
- Scanning: the background was corrected by removing the sample and creating a smooth background image. A beam centering was conducted, the sample mounted on florist foam was loaded, and the image acquisition process was started. The process was monitored to correct for any errors.
- Image reconstruction: reconstruction settings used for the chameleon scan included cropping to remove unwanted regions around the edges using the manual crop editor, selecting the 16-bit data type, and correcting for offset by using a scan optimization process. Additionally, a low beam hardening correction value and a background intensity value were used to correct for variations in intensity. The reconstruction process resulted in a single data file with a size of 6.3 gigabytes.
- Image visualization: the 3D visualization of the chameleon is shown in Fig. 5, A and B. A simple thresholding function (see the Glossary) allows for the visualization of the skeleton structure, which is notably denser than the rest of the animal (Fig. 5).

### Scanning at higher resolution

**Figure 6:**
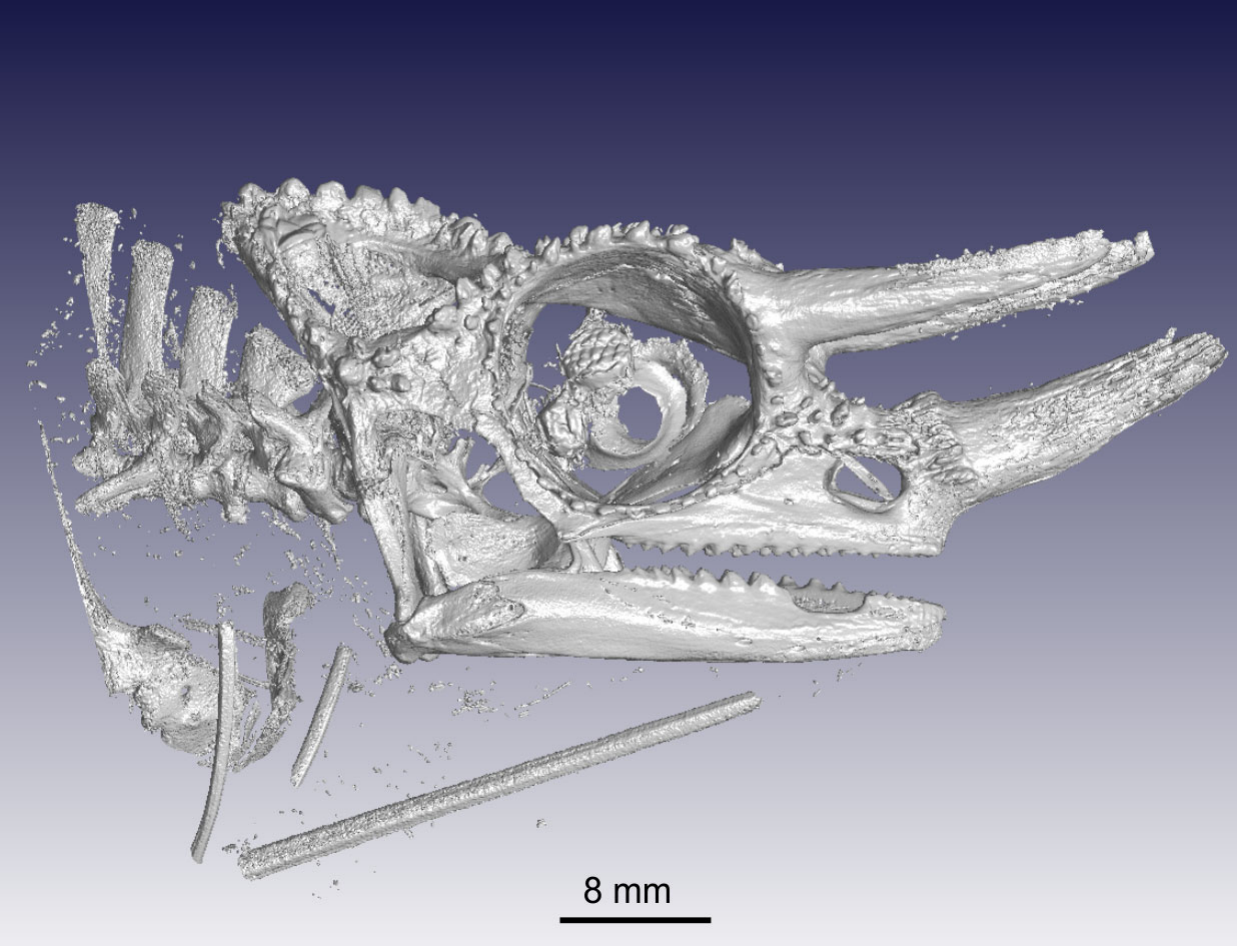
A high-resolution (30 μm) scan of a Jackson's chameleon showing the skeletal elements present in the head.

As discussed in the “Sample size versus resolution” section, the choice of resolution is perhaps the most important factor for data collection planning. Here, we briefly illustrate the differences between resolution settings using the example chameleon. First, the full body scan (resolution: 75 μm) is compared to a close-up of the head scanned at 30 μm. Figure 6 demonstrates that a higher resolution allows smaller features (e.g., skeleton structures) to be visualized. As mentioned earlier, a higher resolution (e.g., 30 μm) can be used to scan the entire sample with an automated multiple-scan process in which a sequence of scans are performed at different height positions across a vertically mounted sample. The multiple scans can afterwards be stitched together to form a large data set. However, it should be noted that this can be a time-consuming process (Fig. 6).

**Figure 7:**
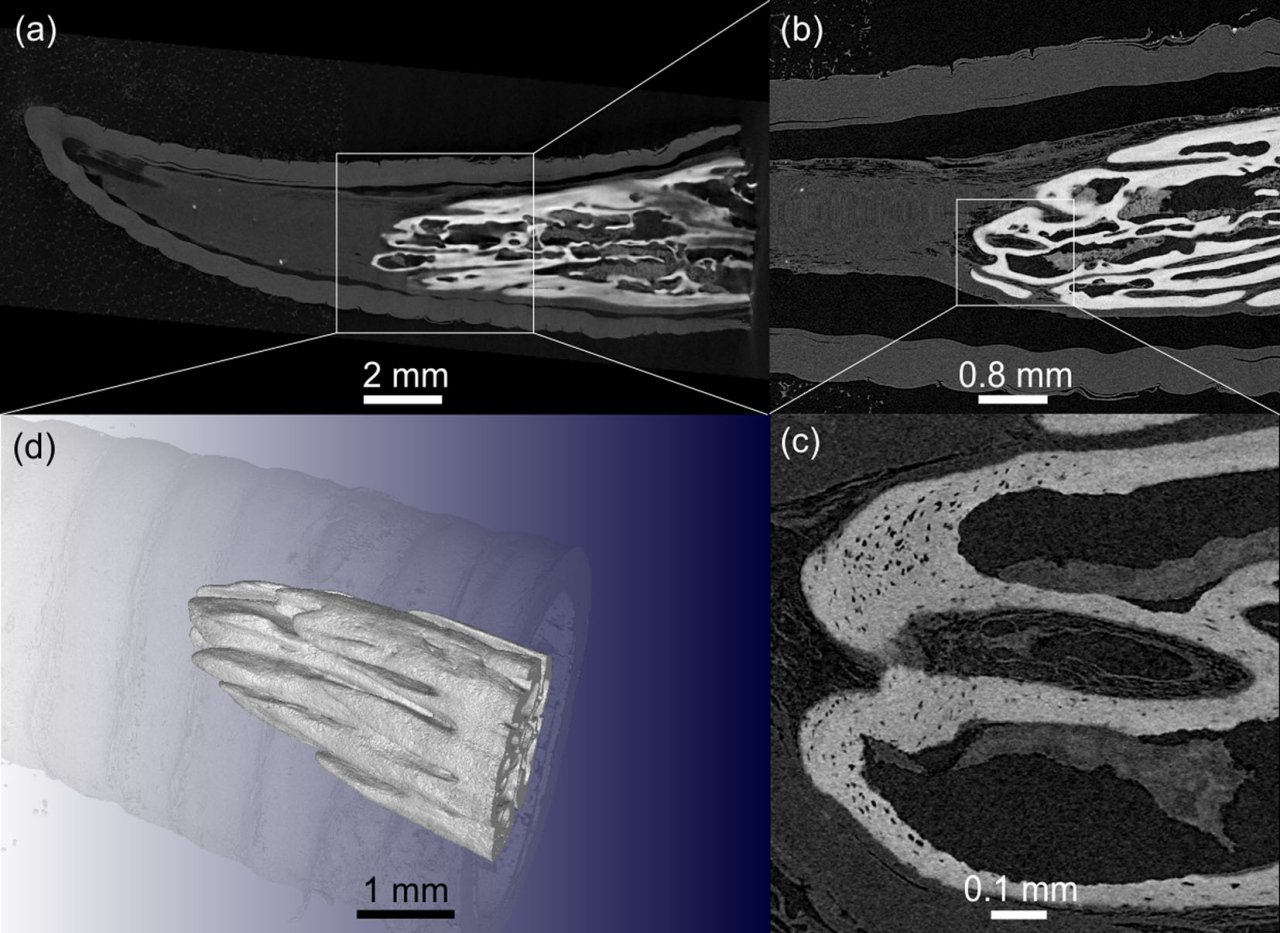
Slice images of the horn of a Jackson's chameleon obtained by using nano-CT showing the bony core at 10 μm **(A)** and 4 μm **(B)**. At a very high resolution of 0.95 μm **(C)**, the bone micro-architecture becomes clearly visible. A 3D rendering of the structure of the bony core inside the chameleon horn is visualized in **(D)**.

Secondly, the horn of the chameleon was scanned after dissection to obtain sub-micron resolution. The improvement in resolution (from 10 μm to 0.95 μm) is depicted in Fig. 7, A–D. The sub-micron resolution allows the user to obtain detailed information on, e.g., the bone micro-architecture of a sample. The 10 μm scan was conducted using a nano-CT instrument, but it must be noted that most micro-CT models are able to achieve this resolution, with some models allowing up to 4 μm. The choice of nano-CT vs micro-CT instrument depends primarily on the sample size and resolution required – typically image quality is better when using the nano-CT for samples smaller than 10 mm. The sub-micron resolution in particular allows clear viewing of the horn microstructure, which can be used to accurately measure bone volume fraction, for example (Fig. 7).

## Summary

In this paper, we aimed to provide a “how to” guide for new users unfamiliar with micro-CT to obtain a better understanding of the technique. In addition, we provided suggestions and guidelines that can be used during research planning and facilitate the interaction between researchers and CT operators and/or facilities. An example, the Jackson's chameleon, scanned at various settings, was used to illustrate the procedure, and by making use of the guidelines, users can adapt the procedure to suit a variety of study objects or organisms.

## Glossary of terms

Background normalization: the background intensity of the 2D detector is calibrated to equal values without any sample in the path of the x-ray beam.

Beam filtering: the x-ray beam is filtered using thin plates of copper or other material to precompensate for beam hardening.

Beam hardening correction: a software correction factor is used in the reconstruction to compensate for beam hardening artifacts.

Clamping: a software correction factor is used in the reconstruction to limit the range of gray values and thereby vary the contrast in the image.

Cupping (artifacts): beam hardening causes a brighter region around the outside than the middle of the sample, causing a gradual variation from middle to side, referred to as a cupping effect.

Polychromaticity: the x-ray beam contains a range of wavelengths, making it polychromatic.

Ring artifact: due to the sample rotation, slice images may show rings around the center of rotation.

Surface rendering: 3D data view of the surface/edge of the sample.

Thresholding function: selecting the edge of material based on a gray value threshold.

Tomography: creating slice images of a sample, thereby viewing its internal details.

Volume rendering: 3D data viewing.

Voxel: volumetric (3D) pixel.

## Abbreviations

2D: two-dimensional; 3D: three-dimensional; CAD: Computed Aided Design; CAT: computerized axial tomography; CT: computed tomography; μm: micrometer.

## Supplementary Material

GIGA-D-16-00031_Original_Submission.pdf

GIGA-D-16-00031_Revision_1.pdf

GIGA-D-16-00031_Revision_2.pdf

GIGA-D-16-00031_Revision_3.pdf

Response_to_reviewer_comments_Orginal_Submission.pdf

Response_to_reviewer_comments_Revision_1.pdf

Response_to_reviewer_comments_Revison_2.pdf

Reviewer_1_Report_(Revision_1).pdf

Reviewer_2_Report_(Original_Submission).pdf

Reviewer_2_Report_(Revision_1).pdf

Reviwer_1_Report_(Original_Submission).pdf

## Availability of supporting data

Supporting micro-CT data of the Jackson's chameleon example presented in the paper are available for inspection from the *GigaScience* database (GigaDB) repository [20].
