# Supplementary material for: Laboratory x-ray micro-computed tomography: a user guideline for biological samples
Source: Gigascience. 2017 Apr 17;6(6):1–11. doi: 10.1093/gigascience/gix027 (PMC5449646; doi:10.1093/gigascience/gix027)
Supplement: GIGA-D-16-00031_Original_Submission.pdf [file gix027_GIGA-D-16-00031_Original_Submission.pdf]

Manuscript

Click here to download Manuscript CT manuscript 8 June 2016 GigaScience.doc

Click here to view linked References

Laboratory X-ray micro-computed tomography: a generalised approach for biological samples using a three-horned chameleon as example

Anton du Plessis[1,2, a *], Stephan Gerhard le Roux[1,b] & Anina Guelpa[1,c]

[1] CT Scanner Facility, Central Analytical Facilities, Stellenbosch University, Stellenbosch, South Africa

[2] Physics Department, Stellenbosch University, Stellenbosch, South Africa

[a] anton2@sun.ac.za , [b] lerouxsg@sun.ac.za, [c] aninag@sun.ac.za

* Corresponding author

Abstract

This paper provides a detailed “how-to guide”, describing many important concepts for users of micro-computed tomography (micro-CT) facilities to consider when planning their work. In this process the method is described in technical detail, explaining the important choices with regards to scan setup and parameters. A unique three-horned chameleon is presented, which was scanned at different resolutions and accompanied by simple analysis. Full data sets of this three-horned chameleon are provided as supplementary information. The aim is for new users to gain experience in working with typical micro-CT data and to be able to view analysis results in 3D. Besides being a fascinating test sample, this species of chameleon has not been analysed in such detail before. It is important to note that the technical detail and discussion is relevant to all commercial types of micro-CT instruments and we hope this paper will assists researchers in making better use of this powerful and emerging technology.

Keywords: Industrial CT, X-ray tomography, micro-computed tomography, nano-computed tomography, 3D imaging, non-destructive testing, three-horned chameleon

1. Introduction

The ability to perform non-invasive analysis is often of prime concern when working with biological samples. Therefore, much attention has been given to find techniques for the inspection of internal properties or the evaluation of quality attributes from biological samples, non-destructively. One such technique, computerised axial tomography (CAT) or medical computed tomography (CT), is widely used for non-destructive imaging of the internal organs and structures of the human body [1]. Generally, this method involves the recording of two-dimensional (2D) X-ray images from various angles around an object, followed by digital three-dimensional (3D) reconstruction. More generally, CT can refer to any form of penetrating radiation being used to record 2D images of any object from different angles followed by reconstruction of these images using digital algorithms, into a 3D data set. Thereby, the data from CT results in a virtual rendering of the object under investigation. This allows one to virtually travel through the volume in any direction and angle, view selected internal features, or make dimensional or volumetric or other more advanced three dimensional measurements [2, 3].

Industrial X-ray computed tomography is a specialised form of CT scanning meant specifically for non-medical applications (hence the term industrial) and frequently involves resolutions in the micro-meter scale. The method is therefore termed micro-computed tomography (micro-CT) and in the case of sub-micron resolution, such methods are termed nano-CT or sometimes X-ray microscopy, as the resolution is similar to optical microscopes. Other terms in general use are X-ray CT (XCT), industrial CT, and laboratory X-ray tomography. Industrial CT differs from medical CT in two major ways: firstly, the resolution is potentially much improved and variable in micro-CT systems, due in part to the stable fixed source and detector and a rotating sample (in contrast to medical CT where the sample is stationary and the source and detector move around it); and secondly, industrial CT is not dose-limited and have a wide variety of choices with regards to voltages, currents and beam filtration. X-ray micro-CT systems can also vary considerably in system capabilities, from small low-cost benchtop systems to cabinet systems able to house larger samples and even as large as walk-in cabinet systems with multiple X-ray sources. Similarly, different manufacturers provide different hardware and software options. These systems should not be confused with synchrotron CT, where synchrotron radiation is the X-ray source, as opposed to laboratory generated X-ray radiation [4]. Synchrotron CT differs from laboratory CT as it has much higher brightness X-ray radiation, allowing much shorter scan times for similar or better quality, and allowing higher resolution on larger sample sizes than laboratory CT. However, synchrotron CT is not as widely available or as easily accessible as laboratory CT and such work must be planned months in advance. Since laboratory CT is available widely, in some cases in open access format, planning is limited to weeks or even days.

X-ray micro-CT has numerous applications and is useful in any scientific field where non-destructive analysis is required. The versatility of this technique is shown in the number of reviews that have been published recently, such as in food sciences [5], the geosciences [6], biology [4] as well as materials sciences [7, 8].

Despite its numerous applications and potential, the method is still underutilised as new users struggle with the mystery that surrounds the entire scanning process, including sample preparation, scanning, reconstruction and choices for analysis types. Lack of knowledge regarding the mentioned processes could result in poor scan quality, inefficient use of facilities or the inability to extract required information for the required research purpose. Some work has been published in a book chapter focusing on bone microstructural analysis, where details of the CT scanning process have been outlined to assist in de-mystifying the process, especially for new users to the technique [9]. A similar work was presented by Mizutani and Suziki [4], where the focus was on soft tissue scanning and staining methods to enhance contrast. In the present data note, we focus on new users from general biological backgrounds and present a multi-scale investigation of a three-horned chameleon specimen. We add the full data sets of the chameleon scanned at 75 µm, 30 µm, 10 µm, 4 µm and 0.95 µm, to aid new users and researchers who might not have access to funding for obtaining their own micro-CT data sets. Hopefully this work will lead to more effective use of micro-CT facilities, through an improved understanding of the capabilities and limitations of the technique.

2. Background to computed tomography

X-ray micro-CT as a technique makes use of information from projected 2D images as obtained by a X-ray source and a detector to investigate the internal structure of a sample [10]. The fundamental components of any micro-CT instrument are (1) penetrating ionising radiation, (2) a sample manipulator and (3) a detector [11] (Fig. 1). The basic principles of X-ray micro-CT are generally described in Kak and Slaney [12]. X-rays are generated using a micro-focus X-ray tube, which uses a beam of electrons accelerated by a voltage of up to 240 kV or more in a vacuum tube, and are focused onto a tungsten metal target (or other metal target material, though tungsten is most widely used). The fast moving electrons hitting a metal target material creates X-rays. In a micro-focus X-ray tube the resulting cone beam of X-rays is directed through and around a sample, before being collected on a 2D X-ray detector in the form of a “shadow image”, also called a projection image or radiograph [3]. The sample manipulator (or rotation table) positions the sample in the path of the radiation beam and rotates it through a specific angle (usually 360°). The detector converts the attenuated radiation, which passes through the sample along a straight line, into the 2D digital images, consisting of thousands of pixels. In this way, many hundreds or thousands of 2D projection images are recorded during the scan process. After scanning, these images are used to reconstruct a three dimensional data set by making use of filtered back-

projection algorithms [13]. Effectively, every volumetric pixel (or voxel) was imaged (by 2D projections) from many angles, and the sum of its view from every angle produces a good representation of the actual X-ray density and hence brightness of that voxel [3]. Following reconstruction, data visualisation and analysis is possible using a variety of software tools, some more complex than others. These steps are all described below with discussion of practical considerations.

Insert Figure 1 approximately here

3. Computed tomography (CT) basics

The steps associated with X-ray micro-CT include: set-up considerations (sample preparation and mounting, sample size vs. resolution, step positions and averaging, voltage settings, filtration of beam and detector and penetration ratio), scanning, reconstruction, visualisation, image processing and analysis, as well as further scanning at higher resolution. These steps will be explained, where applicable, by means of an example: a three-horned chameleon, the Jackson's chameleon (*Trioceros jacksonii*) (Specimen number USEC/H-2927 from the Stellenbosch University Zoology/Botany Department's collection) scanned using a Phoenix V|Tome|X L240 (General Electric Sensing and Inspection Technologies / Phoenix X-ray, Wunstorff, Germany) micro-CT system, as well as a Phoenix nanotom S (General Electric Sensing and Inspection Technologies / Phoenix X-ray, Wunstorff, Germany) nano-CT system, both located at the CT Scanner Facility of the Central Analytical Facility (CAF), Stellenbosch University, South Africa (http://blogs.sun.ac.za/ctscanner/).

3.1 Set-up considerations

A scan set-up involves a good choice of parameters based on some general guidelines, experience with different sample types and requirements of the analysis. Five general guidelines (*Guidelines I* to *V*), along with explanations will be presented in this section for careful consideration when performing micro-CT measurements of biological samples.

3.1.1 Sample preparation and mounting

Amongst analytical techniques, micro-CT requires very little, if any, sample preparation. Generally, a sample can be scanned exactly as provided, without any sample preparation. However, staining is sometimes necessary when poor material discrimination is found, i.e. biological soft tissues. X-ray specific staining (or labeling) is performed with contrast media (iodine or barium or many others) [4]. Briefly, a sample can be soaked in a contrast agent (such as a liquid solution containing iodine) for a period of time and then removed, dried and mounted for micro-CT scanning. Staining is not something that should be attempted as a first experiment, and should only be used in special cases and when all other options have failed to improve contrast.

To obtain best scan quality it is important to load the sample properly. Samples should be loaded at a slight angle to ensure that parallel surfaces to the X-ray beam are minimised. Parallel surfaces are not penetrated properly and lead to image artifacts and lack of detail in the data set in the plane of the flat surface parallel to the beam.

The mounting involves a low-density material (cardboard tubes, plastic bottles or glass rods) which holds the sample in place on a rotation stage (turntable), but separates the sample from the dense rotation stage hardware. The most important parameter in a good scan is ensuring that the sample does not move during the scan time. This is more relevant for longer scan times. If a sample suddenly moves due to mounting tape coming loose during the scan, it will result in a blurred 3D image. In the same way, a wet sample that dries out during the scan (causing shrinking) will also cause a blur. For wet samples, such as biological preserved specimens, there are different approaches that can be used to overcome this problem. One approach is to dry the sample before scanning, or to make use of freeze-drying. Another approach is to wrap the sample in a wet cloth, thereby keeping the sample wet for an adequate period. It is also possible to scan samples inside liquid filled tubes, although it can be challenging to keep the sample in place. Additionally, if it is kept in place by the edges of the container, these edges will not be separable from the sample in the image processing steps. Another approach to minimise blurring is to use very fast scan settings, although this is not always possible when highest quality and resolution is required.

Some mounting options are presented in Figs. 2a, 2b and 2c, respectively showing a specimen covered with a wet cloth, mounted upright for vertical, multiple-part scanning, and lastly, a nano-CT mounting. The wet-cloth mount is useful for keeping a sample wet during a scan, and is useful when only the internal features are of interest, since the cloth and the skin of the sample are not digitally separable in the scan data. The vertical mounting method is useful when multiple-scans at higher resolution are done of different sections along the height and then stitched together to form a higher resolution complete data set. The nano-CT mounting is also used in similar form as with micro-CT of smaller samples, where a glass rod is used and the sample is mounted on top of this rod with double sided tape, glue or using a small cube of foam stuck to the rod. Other options include rigid foam stuck to the top of a glass rod, fitted with a small cavity or slit, plastic film covering soft tissue or wet samples and soft foam for wrapping a sample placed on top of the glass rod.

Insert Figure 2 approximately here

3.1.2 Sample size vs. resolution

Micro-CT instruments can accommodate sample sizes from as large as 40 cm (or larger in some systems) to as small as several micrometers [6]. Large walk-in cabinet systems allow the detector to back up as far as 1.6 m from the X-ray source, which allows samples as large as 40 cm to be loaded and fit within a single scan volume, with the usual single scan volume covering approximately 30 cm at its furthest position. Smaller cabinet micro-CT systems allow generally a 1 m source-detector distance allowing up to 15 cm in a single scan. Benchtop instruments have further limitations on sample size and might require sectioning of samples in the preparation process, although this may not always be a drawback since benchtop instruments are lower cost and more readily available than larger systems.

The choice of resolution is the first major factor affecting a micro-CT scan. Useful guidelines *(Guideline I)* when estimating the best possible resolution for a sample of known dimensions are:

i. The best commonly used resolution is a factor 1000 smaller than the width of the sample. This means for a sample 100 mm wide, the best resolution is typically 100 µm

The above guideline is based on the standard practice of using only the central 1000 of 2000 available pixels of the detector to minimise possible artifacts from the edges. Most detectors have 2000 pixels but some systems have more, which allow improved magnification for the same sample size, but might introduce other problems such as data set size and long reconstruction times. It is in theory possible to use all 2000 available pixels in the above example, in which case up to 50 µm is possible. However, besides the potential for artifacts from the edge regions, it is practically very difficult to mount a sample perfectly central on the rotation axis in order for it not to move out of the field of view during a rotation, and this process could also be very time consuming at high resolution.

These guidelines are generally applicable to most modern micro-CT instruments, which typically have 2000 detector pixels and a graph depicting resolutions obtained vs. sample sizes is presented in Fig. 3. In general, resolutions of micro-CT scanners are in the range of 5 – 150 µm (0.005 to 0.15 mm), compared to medical CT scanners having best resolutions of 0.7 mm. Generally, nano-CT scanners have resolutions down to 0.5 µm (500 nm) in laboratory instruments (some higher resolution instruments are available, but not widely available yet).

Insert Figure 3 approximately here

3.1.3 Resolution, voxel size and X-ray spot size

The voxel size of a micro-CT image is dependent on the magnification and object size as described above. This is related to the distance of the sample from the X-ray source and the

detector [5]. Voxel size and spatial resolution are two concepts that are often confused, since the voxel size is the size of a pixel in 3D space, i.e. the width of one volumetric pixel (isotropic in 3 dimensions). This value does not consider the actual spatial resolution capability of the scan system. If the X-ray spot size becomes larger than the chosen voxel size, the spatial resolution of the system becomes poorer. Since most commercial systems limit the size of the X-ray spot to the required voxel size (or provide the user an indication of this), the actual and voxel resolution are usually the same, but this is not generally tested or reported. It is possible to use resolution standards (such as calibrated-thickness metal wires) to confirm spatial resolution and some reference standards exist although a generally accepted standard for industrial CT systems does not exist. It is therefore possible that the amount of detail that is detectable in a scan can vary considerably from system to system, or even between different scans from the same type of system. This is due to improper settings that possibly result in large X-ray spot sizes, or due to improper choice of other scan parameters.

When scanning an object at different resolution settings, the same object with a better resolution (higher magnification) will cover a larger portion of the detector resulting in more angular positions required for a good reconstruction. This affects the total scan time, making a higher resolution more time consuming. This factor is discussed in the next section in more detail.

3.1.4 Scan time, number of images and rotational options

The major consideration for scan time is the acquisition time of single projection images, which can vary from system to system due to detector sensitivity and dynamic range differences, X-ray tube brightness differences, and differences in physical distance form source to detector [3]. A typical image acquisition time in a walk-in cabinet system with a 16-bit flat-panel detector is 500 ms per image, while some benchtop systems have typical image acquisition times of 2000 ms per image. All systems have variable image acquisition times and therefore scan times can vary considerably. For highest possible quality scans, keep in mind to make full use of the dynamic range of the detector. In doing so, the image contrast is maximised by raising the image acquisition time up to near saturation of the detector for a particular X-ray setting. If the image acquisition time is too low, the resulting contrast will be poor with grainy images in extreme cases.

Some scanners involve continuous scanning (continuous rotation and image acquisition without steps), but the discussion here is limited to a stepwise rotation for simplicity. At each step position, one or more images can be acquired and averaged to provide an improved image quality (compared to a single image per position). Averaging reduces noise and therefore improves image quality, but its effect depends on the inherent noise of the detector used. For samples which may have small vibrational movements during rotational movement, e.g. leaves or hairs, it is advisable to use the skip function if available, which ignores the first image acquired at each new step

position (during which time the sample stabilises). Since this vibration is due to the stepwise process, another approach is to use continuous scanning which also reduces vibration but in that case averaging is not possible.

Generally, the number of step positions required depends on the sample size relative to the magnification. Therefore, the higher the magnification and hence the number of pixels used on the detector, the larger the number of images required for a good reconstruction.

A useful guideline in this regard *(Guideline II)* is:

i. The number of pixels covered by the sample on the detector in width multiplied by 1.6 equals the number of projection step positions required. Consequently up to a maximum of 3200 step positions are used for a typical 2000 pixel wide detector.

This important concept therefore plays a large role in scan time determination.

3.1.5 X-ray parameters: voltage, penetration values and background intensity

X-ray projection images or radiographs can be viewed live and can be used for non-destructive analysis without the use of 3D reconstruction. In fact this method of “digital radiography” is in wide use for industrial non-destructive testing purposes [14], though it is limited for complex objects and does not provide depth information, e.g. the presence of a void can be determined but not how close it is to the surface or how close to other features in the line of sight. It is sometimes useful to use the live digital X-ray projection image as a fast scout method to quickly assess the inside of an object, thereby determining if a full CT scan is necessary (to provide a more detailed 3D view). An example of an X-ray projection image of the chameleon is given in Fig. 7b.

For CT scan setup, the radiograph or projection image is used as a basis for selecting good X-ray parameters, based on the sample’s penetration values and the background intensity values. Different types of samples require different X-ray voltages for best quality and this cannot always be predicted or estimated before a scan. The best possible material discrimination is obtained by using lower voltages. However, if a dense object is present, the X-ray penetration will be insufficient (sometimes unexpectedly) causing noise and artifacts. Beam hardening is the most common CT artifact and occurs due to the fact that the X-ray beam is polychromatic, with a range of low and high energy X-rays present, and because low energy X-rays are absorbed more easily than high energy X-rays. This results, for dense objects, in differences in absorption from different angles resulting in streaky artifacts across the image in the 3D data set.

If too high voltage is used, very poor contrast is obtained between different materials. These factors are important for a proper choice of scan parameters but also for sample preparation. For example, consider the scan of a biological museum specimen (preserved small animal) with a metal name tag. It is better to scan such an object without its metal name tag, than with it. The metal is much denser for X-ray penetration than the sample itself, resulting in beam hardening artifacts at the best (low voltage) settings for the scan. By increasing the voltage, this limitation can be overcome and the metal tag can be scanned properly, but with a major reduction in quality of the image of the biological specimen. Fig. 4 shows examples of scans of the chameleon (a) with a metal tag in the scan resulting in streaky artifacts (b) at too low voltage causing some minor artifacts around dense internal features and (c) at too high voltage showing poor contrast.

Insert Figure 4 approximately here

The choice of voltage is strongly related to the sample type and the goal of the scan, but some general guidelines (*Guideline III*) are as follows:

i. biological samples: 30 to 100 kV;
ii. small rocks and light metals: 60 to 150 kV;
iii. heavy metals (e.g. steel) and larger rocks: 160 to 240 kV or more; and
iv. in general: the smaller a sample the lower the voltage that is possible.

When the voltage is increased, it is often useful to add beam filters to pre-compensate for beam hardening and effectively reduce the polychromaticity of the beam, thereby preventing streaky artifacts. Commonly used filters include from 0.1 up to 2 mm of copper and from 0.5 to 1.5 mm tin or combinations of these. Other beam filters can be used, such as aluminum that is a popular choice. The selection of beam filters is usually more relevant to very dense objects and not biological samples. In the case of most biological samples no beam filtration is required or when required, at most 0.5 mm copper or aluminum. Detector filtration (using a filter between the object and detector) can also be used in specialised cases to reduce noise, for example where the sample produces secondary X-ray emission, especially when very dense objects are scanned, or when a large amount of scattering is present.

Guidelines IV are presented for the determination of adequate penetration values:

i. A typical setup method to find best settings for a particular sample type, is to rotate the sample until its 2D X-ray projection image shows the darkest region (its longest or densest axis) and then to calculate the sample's minimum penetration ratio compared to the background X-ray intensity (using the grey value counts measured in the X-ray image).
ii. If the penetration value is less than 10%, an increased voltage or current is required.

iii. However, this might cause the detector to saturate, in which case beam filters can be applied to prevent saturation, while still increasing the penetration value.
iv. By using a beam filter, a higher voltage or current can be obtained with a reduction in the low energy X-rays such that the detector does not yet saturate.
v. Penetration values from 10 % to 90 % should result in good scan quality.
vi. In order to reduce penetration, lower voltages should be used.
vii. Since X-ray emission is typically very limited below 50 kV, longer scan times are then required to obtain large enough signals.

3.1.6 Scan quality problems and artifacts

As can be seen above from the many variations of scan settings, different quality scans can be obtained. Figs. 5 shows micro-CT slice images of the chameleon with poor contrast, double edge and slight blur, respectively. These are typical image quality problems that can occur. In particular, Fig. 5a has poor contrast, in this case due to incorrect reconstruction setting (clamping) which will be discussed in Section 3.3. The same effect will occur when scanning a sample with the metal rotation table in the scan volume, or when too low voltage is used. Fig. 5b has a double edge due to incorrect reconstruction setting (offset correction) which will also be discussed in Section 3.3. The same type of double edge will occur to a lesser degree if the sample moves during a scan. Fig. 5c shows only a slight blur on the edges, which is due in this case to hardware misalignment, where the rotation axis of the sample and the X-ray tube direction is slightly more or less than 90 degrees (referred to as the tilt axis). These images are meant to demonstrate typical image quality problems.

Insert Figure 5 approximately here

Beam hardening was already mentioned above when discussing choices of voltages, within the context of streak artifacts. However, for homogenous dense objects scanned with too low voltage, a “cupping” effect is also possible where the edge of the sample seems brighter than the inside. This artifact is present when insufficient penetration of the sample is obtained, for example due to too low voltage. Other artifacts and unwanted image effects include cone beam artifacts affecting the edges of materials near the edges of the detector, double edges due to tilt axis misalignment relative to beam axis, and blurring due to unstable rotational axis.

3.2 Scanning

Before a scan commences, the background must be normalised by removing the sample and using the X-ray beam at the chosen settings, and correct for all intensity variations across the detector (e.g. the beam is more intense in the middle than the edges of the detector, but the normalised pixel intensity must be equal for a good scan). This normalisation or correction process

can be done before every scan, but is only required if X-ray or acquisition settings change, or at the start of a day of scanning, for example. It is also necessary to run a beam centering right before scanning to ensure the electrons are well focused resulting in optimal X-ray output.

Once the sample is loaded and settings chosen, a scan can be acquired. The scanning is done automatically with no user interaction, but errors can occur during the scan, requiring frequent supervision. For example, the X-ray source could become unstable requiring warmup, the filament could burn out requiring replacement, or other errors could occur requiring attention to either continue the scan as soon as possible, or re-start the scan once the problem is solved.

Due to long scan times, the potential for low image quality or artifacts and the requirement to find optimal settings for particular sample types, or due to large numbers of samples requiring scanning, fast scans are sometimes required. Such fast scans (e.g. 15 min) are not ideal, but can be sufficient for some purposes, such as identification of relatively large features, finding regions for further analysis or for higher quality scanning, or for simple measurements. Such fast scans can also be used as a scout method, to find a region of interest for a long, high quality scan.

3.3 Reconstruction

Reconstruction entails the creation of a 3D data set from 2D image projections and different options are available, as well as different data output types, as presented in this section.

3.3.1 Reconstruction options

After a full scan, the data is reconstructed which refers to creating a discrete 3D data set from 2D image projections. Sometimes a reconstruction refers to a 3D image rendering or visualisation of a 3D data set, but we call this instead “visualisation” and describe this in the next section in more detail. The reconstruction process involves effectively the mapping of each voxel, by using projection image representations of that particular voxel from many angles. This mapping is done by a Feldkamp filtered back-projection algorithm [15]. Commercial micro-CT systems all have built-in reconstruction software packages with slightly different available settings but all based on the same algorithms. General Electric uses a software called Datos. Volume Graphics is a stand-alone software package mainly used for 3D image analysis but also offers a module for reconstruction which is very similar to Datos, and another commercial standalone software for reconstruction is offered by Inside Matters, called Octopus Reconstruction.

Reconstruction software involves a series of choices or options, which can also affect the quality of the obtained 3D data. These options will be described in general here, though not all options are available in all reconstruction software packages.

i. Firstly, the field of view can be cropped to make the total reconstructed volume smaller. This helps reducing the data set size and makes reconstruction faster since less memory is required, especially helpful when time or computing power is limited.

ii. A choice of data type can be made which is usually selected as 16-bit, but 8-bit can be selected if storage space or memory is a problem.

iii. The exact location of the rotation axis in each projection image is found by making use of an automated algorithm which finds the central pixels in all 2D X-ray images – the use of the exact rotation axis in the back-projection algorithm improves the quality of the reconstruction and is especially important at higher resolutions.

iv. This process can also be coupled with a refinement process correcting for small movement or shift of the sample or due to inaccuracy of the rotation stage. This can be done by viewing before-after projection images and correcting for any small shifts. In advanced software packages, this can be done at more than one position during the scan, correcting for changes which occur at different times during the scan. This process is limited to very small changes but does result in much improved edge clarity in the reconstructed data set.

v. Another important choice is beam hardening correction, which corrects much of the generally-occurring “cupping” effect in samples where the edges seem brighter than the middle of the scan. This can be attempted by trial and error until the best value is found (values can be chosen in a range from light to heavy implementation of the algorithm).

vi. Another option sometimes available, is when it is possible to disregard a certain % of pixels that are “outliers” in terms of strong or weak absorption compared to the rest of the data, which effectively improves the grey value contrast in the images. This is called “clamping” and can be very useful when a small quantity of bright dense phases are present but are not of interest, and are therefore all grouped as the same brightest grey value. The % of pixels that are clamped, and the clamping direction (lowest or highest grey values only, or both) can be set.

vii. It is also possible to make use of special settings to select the background detector counts in each image and normalise this across the series of images, which is useful when scattering is present resulting in brighter or darker projection images from different angles.

viii. It is possible to use special algorithms to remove ring artifacts by disregarding “dead” pixels from the 2D projection images. Ring artifacts especially near the center of rotation are also removed by making use of a detector shift process whereby the detector shifts horizontally between step positions and which are corrected in the reconstruction process resulting in a smoothing of the rotational center artifact.

ix. It is also possible in region-of-interest (ROI) scans (where the sample extends over the sides of the 2D image), to remove the bright ring which results around the outside of the scan volume and hence improve the image quality by using a special algorithm dedicated for this purpose.

Therefore, it is clear that many different possibilities exist for reconstruction and hence this process is in itself an important step, which can assist in obtaining improved image quality even when using lower cost CT scan hardware. Since the reconstruction process itself can vary significantly, it is suggested to retain X-ray projection images even when reconstruction has been completed, thereby allowing future improved reconstruction of the same data, where possible.

In medical CT, each voxel is typically associated with a calibrated Hounsfield Unit or CT number, which is regarded as the average attenuation in that section of the sample [16]. However, in micro-CT the grey values are not calibrated and depend on reconstruction settings in the software used, as well as various scan parameters. In general, therefore the reconstructed data from micro-CT scans are not calibrated for density determination.

It is also possible to use resolution reduction or oversampling in the reconstruction process, as well as filtering of the 2D projections using a variety of image filters. These are not standard and only used in specialised cases. Another seldom used option is to reconstruct scans of less than 360 degrees, when a sample is too large to rotate fully in the system used, for example. It can be mentioned here that in the case of data collection, sometimes an image could get corrupted or lost. A simple process of replacing this image with its adjacent image in the sequence, results in a good reconstruction, even though an image may be missing.

Insert Table 1 approximately here

3.3.2 Data output types

Both the X-ray projection images captured by the CT system and the reconstructed CT dataset consist of pixels or voxels which have specific shades of grey. Each of the pixels or voxels are assigned a specific value which indicates the intensity of its shade of grey. These values differ depending on the bit depth assigned to it e.g. 8 bit = 2^8 = 256 which indicates the grey value range (in integral values) is from 0 to 256 and 16 bit = 2^{16} = 65536, which indicates that the grey value range is from 0 to 65536. The advantage of the 16 bit dataset over the 8 bit dataset is that it has more capacity to differentiate between small density changes because of the larger grey value range providing a more accurate representation of the sample that was scanned. In general commercial micro-CT scanners have detectors with dynamic range of between 12 and 16 bit, so using 8 bit data sets result in loss of information. The advantage of 8 bit data types is the much smaller physical size (50% reduction) and hence easier handling for visualisation, analysis and transfer of data.

A general comment about image file types is warranted here. There are different image files types with the main difference being JPG and BMP and similar which are Windows compressed image formats and TIFF which is uncompressed. The compressed file formats are lower quality and can results in loss of information. Though the use of a compressed file format can reduce data set size, it is advised to rather use poorer resolution scans to reduce data set size. In order to achieve this, during the setup step, the sample is moved back such that it fills less of the field of view of the detector which will result in a smaller data volume (and shorter scan time). Also worth mentioning is that 16-bit TIFF files cannot be viewed properly by windows image viewer programs. High quality image viewing programs such as Irfanview are required.

Image stacks are calibrated in the sense that each isotropic voxel has a side length equal to the scan's voxel size. However, post-processing of this data and creation of images for viewing, including scale bars, do not necessarily maintain this pixel spacing, especially when creating lossy file types such as JPG. The original image stack must therefore be kept together with knowledge about the voxel size. In some scanner types this information, together with all scan settings, are kept in a file which can be read in notepad (e.g. PCA file in General Electric Phoenix systems or TXT file in Nikon systems). Saving image stacks with a scale bar from image processing software is not suggested for two reasons: firstly, these scale bars are not accurate enough (rounded off) and secondly, having a scale bar in a slice image creates a long 3D scale bar, which is not useful.

3.4 Visualisation

Typically, volume analysis and visualisation is done in a 3D data analysis software package. These differ from 3D Computed Aided Design (CAD) software in that they handle full voxel data, i.e. data exist everywhere in a 3D voxel grid, not only on surfaces of the object. In other words, CAD software packages use triangulated mesh data of surfaces only (point locations only), while full CT data comprises data at every point in 3D space (grey value at every point). Therefore, a volumetric data set is significantly larger and requires more intensive computing power, even for simple visualisation. Commonly used software available for volume rendering include Avizo, Volume Graphics VGStudio, ImageJ, Blob3D and Simpleware, whereas surface rendering software are AutoCAD, Blender, SolidWorks and Autodesk. Additionally, open source software which can be used for analysis of CT data in 2D or 3D include ImageJ, MIPAR, Blob3D, Quant3D and 3dma_rock.

3.5 Image processing and analysis

The most widely used step in 3D image processing is thresholding. This involves in the simplest case, a selection of a threshold grey value such that all grey values in the volume brighter than this value is selected for viewing or further analysis. This method uses the same threshold across the entire volume and is therefore called global thresholding. Besides this simple thresholding step,

individual features may be highlighted and analysed in detail with regards to its 3D location in the animal. Aligning the slice image to the feature allows the viewing in the correct plane, which is a useful feature for visualisation of different components from different angles in slice images. Its selection was done using a region growing tool, i.e. a voxel is selected inside this feature and all connected voxels with similar grey values assigned to this region of interest. This region can be visualised alone or its colour chosen as demonstrated here, different from the rest of the animal. It is possible to view this in 3D rendering as well by making the rest of the animal semi-transparent and the feature of interest non-transparent. A basic typical image processing procedure is described below but more details of further segmentation and image processing methods are described in Mathews & Du Plessis [17], including a step by step guide for segmentation of frog bones from whole frog scans.

During image processing, filters are initially used to smooth the images and in doing so, to reduce random noise. This is especially useful for noisy data or fast scans, but sometimes this step can smooth over fine structural details, therefore its use is dependent on the image quality and aim of the analysis. Examples of such filters, are Gaussian or Median smoothing.

Segmentation usually follows the smoothing step, where the respective voxels or regions are grouped together, creating individual regions-of-interest (ROIs) or image masks. In some cases this could include a binarisation step, where the selected voxels are given a value of 1 and the rest a value of 0 (or black and white). Thresholding itself can be done in different ways, and the available methods depend on the software used and algorithms available. One valuable method is local adaptive thresholding, whereby a global threshold or region of interest is chosen as starting contour and local thresholding is then applied. In addition to thresholding and region growing, a very useful method especially for biological sample segmentation is manual segmentation using a drawing tool, to add or remove regions with a virtual pen or brush. This method is a tedious process as it can involve colouring in regions in every slice in the CT data set, but is sometimes the best way to segment complex biological data sets. This process can be accelerated by making use of thresholding or other semi-automated segmentation tools and then refining these selections using the drawing tool (instead of making the entire selection only with the drawing tool). Another possibility is to significantly filter the data, which simplifies the automated segmentation tools, but can result in loss of quality for small features. This is useful when viewing only is required, and not complex dimensional or volumetric measurements are required.

The next step usually involves image analysis. Image analysis aims to achieve the extraction of qualitative as well as quantitative data from the images or image sequences [5]. The information from the range of 2D slices that are merged to create the 3D image allow for volumetric observations and measurements of the microstructure of the sample. Some typical tools available

for micro-CT image analysis are: morphology analysis typically used for bone structure (e.g. mean trabecular number, trabecular spacing, volume fractions), defect detection (detection and size distribution analysis of pores/voids/cracks/inclusions); nominal/actual comparison (compare geometries of two voxel data sets based on their surface information); wall thickness analysis (measure thickness variations of walls of objects or layers of a material) and orientation analysis (orientation of fibers or longitudinal features in 3D) [3]. For more details of reconstruction and image processing post-scan, a recent article describes various options in more detail [18].

Analyses vary considerably depending on the application, especially in terms of time required. For some general industrial applications, specialised software modules exist to simplify the process of such analyses and minimise the human error involved in the process. Such routine analyses are therefore not very time consuming (usually less than 1 hour for each type of analysis, sometimes much less depending on complexity of data set). Biological data sets in general tend to be more complex and require more time consuming and sometimes custom procedures to be applied. For viewing of segmented features, as shown above both slice images as well as 3D renderings can be used to allow the visualisation of selected features, for example to view connectivity or location relative to other features. Dimensional measurements can be performed in 2D slice views (linear distance measurements) or in 3D using volume or surface area measurements.

3.6 Further scanning at higher resolutions

In many cases once an analysis has been completed, it might be realised that more scanning can provide further details, especially with regards to higher resolution scanning of selected regions. One further step is therefore to scan a region at a higher resolution to get a more detailed view. The fact that the same sample can be scanned at varying resolutions can be confusing to the first-time user, especially when also considering varying image quality and scan time possibilities. As an example of the application of the guidelines described in the previous scanning setup section: a 20 mm wide sample can be scanned at best at 20 µm resolution with very good quality scan parameters in 40 min (considering 500 ms per image, average 2 images per step position, and 2000 images per one full rotation, plus rotational movement time), assuming no errors and not considering setup time or reconstruction time after the scan. Therefore, in total 1 h per scan is generally used for the micro-CT scanner. Longer scan times are possible with increased image quality, but much faster scans are also possible. What can be confusing is the fact that the same quality scan settings can be used as above for the 20 mm wide sample, at 50 µm resolution. In this case less images are required at 50 µm since less pixels of the detector are covered by the sample. In this case the scan time is roughly halved for the same signal-to-noise ratio (or improving further the signal-to-noise ratio for the same scan time by averaging more). From this it can be understood that the scan time becomes shorter as resolution gets poorer, but increases with an improved resolution when the same sample size is used. Once the best resolution for a

sample is achieved, the next step for higher resolution is to section the sample, or do a ROI scan of a section which will be described in the next section.

Other possibilities for further steps include correlation of CT and other analytical techniques, for example correlation between scanning electron microscopy and CT can hold some advantages, where scanning electron microscopy (SEM) shows detailed surface information, CT can extend this with internal connectivity information, for example of porous materials. Another possibility for a further step is to apply some process to the sample, and repeat the scan producing two or more CT data sets to monitor degradation or changes in internal features in a time lapse (termed 4D imaging).

3.7 Maintenance issues and usage schedules

Commercial X-ray micro-CT systems all require significant maintenance, which must be considered in terms of financial implications. Although benchtop systems require less maintenance than cabinet and walk-in systems, annual services and replacement parts can still be roughly 10% or more (depending on the usage) of the purchase price of the system. For this reason, the availability of local technical support from a supplier is a major consideration, along with the system type. In general, the larger the system the more maintenance is required.

In a multi-user facility where the systems are operational for long periods, streamlining the workflow and getting more scans done semi-automated is of interest. For this purpose, some suppliers offer automated sample loading (e.g. 12 samples pre-loaded in a rotating sample mount), automated choice of scan settings and even sending of emails or phone messages when a scan is completed (or failed). Automated batch reconstruction, as well as batch image processing and analysis is possible on multiple scan data sets done overnight when using macros written by the user. This only works for very simple procedures since no human interaction is involved to check the result, but can be time saving for large numbers of data sets. In practice, however, the most widely used method of getting optimal samples done in the available time span, is to load samples vertically and then scan each one sequentially with a vertical-adjusted multiple scan. In this way, approximately 3-10 samples can be scanned overnight, depending on the scan parameters and assuming no hardware failures occurs during the scan time.

Another misconception regarding micro-CT scanning is the frequency of hardware failures. Especially during continuous-rotation scans, any failure (even only of one image) causes the entire scan to fail. Stepwise rotation limits this problem as the scan is stopped until the system is stable again. However, typical usage for a large cabinet based system requires approximately 10-20 % downtime for repairs and maintenance, as well as consumable replacements (especially filaments)

that are required approximately once a week on average. These are all issues and costs not disclosed by CT companies before a purchase.

3.8 Micro-CT scanning of a three-horned chameleon – an example of data acquisition and analysis

The flow diagram depicted in Fig. 6 summarises the considerations, guidelines and options related to micro-CT scanning of biological samples and is suggested to be used as guiding principles when conducting micro-CT scans and analysis. The X-ray micro-CT data acquisition and analysis of the three-horned chameleon, shown as an example, will follow the step-wise guide as presented in Fig. 6.

Insert Figure 6 approximately here

3.8.1. CT set-up parameters:

i. The chameleon (wet with alcohol from the collection jar) was mounted on florist foam on top of a cardboard tube (Fig. 7a), after being dried out at ambient conditions for a few hours in this position. The chameleon is seen with its densest and thickest features as darker regions by looking at a digital X-ray projection image of the specimen (Fig. 7b).
ii. The chameleon's size was adequate for scanning in a cabinet system (total field of view approximately 100 mm wide x 100 mm high), but would also have been possible in a benchtop instrument.
iii. This example had 2000 pixels in total sample height, and using *Guideline I*, the best possible resolution was obtained at 75 µm. Following *Guideline II*, since the best possible magnification was at 75 µm voxel size, 3200 step positions were used. Since the sample was loaded at 45 degrees, there was a slight improvement in the best possible voxel size compared to horizontal or vertical mounting, for a single scan volume (vertical or horizontal would be limited to longest axis of the chameleon sample).
iv. Averaging was set to 2 and skipping of the first image at each new position was used.
v. As the chameleon is a biological sample, no beam filtration was required.
vi. Initially, a typical image acquisition time of 500 ms was set, with 100 kV and 100 µA as starting point, with no beam filtration. This setting showed a good penetration value, but not very high signal values on the detector, so the current was increased to 200 µA to obtain approximately 8000 counts, where 10 000 is the saturation level of the detector (*Guidelines III & IV*). In this process a compromise between scan time and image quality was found. Higher quality would have been possible with more averaging, resulting in longer scan times. Higher quality would also have been possible at lower voltage since the penetration values were quite high. When lowering the voltage, the total X-ray emission from the source reduces, which requires a longer image acquisition time to make full use of the best

possible contrast capable with the detector. This also increases scan time and additionally, lower voltages can cause unexpected artifacts as explained above.

Insert Figure 7 approximately here

3.8.2. Scanning:

i. Corrected the background;
ii. Ran a beam centering; and
iii. Loaded the sample that was mounted on florist foam and started the image acquisition process.
iv. Monitor the process to correct for any errors

3.8.3. Reconstruction settings used for the chameleon scan included:

i. cropping to remove unwanted regions around the edges;
ii. 16 bit data type selected;
iii. offset correction by using a scan optimisation process;
iv. a low beam hardening correction value; and
v. a background intensity value used to correct for variations in intensity.

3.8.4. The 3D visualisation of the chameleon is shown in Fig. 8 with:

i. a simple thresholding function allowing the visualisation of the skeleton structure which is denser than the rest of the animal;
ii. the data set was processed to virtually remove the mounting material; and
iii. smoothed to produce a clean surface rendering.

Image processing steps are described in more detail in the next section.

Insert Figure 8 approximately here

3.8.5. Image processing and analysis:

As one example, an interesting bony feature is highlighted in red in Fig. 9, this structure is a part of the very strong tongue of the animal (due to its use for catching prey). A slice image shows more detail and is shown here in the plane of the bony feature, indicating that this bony feature is extremely straight. It is possible to view this in 3D rendering as well by making the rest of the animal semi-transparent and the feature of interest non-transparent. An example of image analysis would be to measure the length and width of the bony feature of the tongue of the chameleon, as well as determine its surface area and volume.

Insert Figure 9 approximately here

3.8.6. Further scanning at higher resolutions:

Since the head of chameleon is quite striking and the horns in particular are interesting to study in more depth, a close-up scan of the head of the animal was made. This is a nice example showing how the same sample can be scanned at different resolutions without sectioning, or in multiple ways to get different types of information. In this ROI scan, the resolution was 30 µm (with a field of view of about 30 mm) compared to the full body scan of 75 µm (field of view 75 mm). The higher resolution allows smaller features to be visualised and segmented (isolated) more easily. Fig. 10a shows the 3D surface view of the head with false colour for visual appearance, Fig 10b shows the skeletal structure in white, and Fig 10c shows a cropped view of the skeleton depicting the central horn's internal microstructure. If the entire animal needs to be scanned at 30 µm, this would be possible using an automated multiple-scan process, whereby a sequence of scans are performed at different height positions across a vertically mounted sample. The multiple scans are then stitched together to form a large data set. This process takes longer and is therefore more costly, and is usually only required in specialised situations.

Insert Figure 10 approximately here

Even though X-ray tomography is generally known as a non-destructive technique, sample preparation for very high resolution scans can be somewhat destructive. This is because of practical limitations of sample size. A good example is the horn of the chameleon, which shows an interesting microstructure visible in Fig.10 at 30 µm resolution. If this microstructure needs further and more detailed investigation, scanning it at higher resolution may be necessary. Higher resolution involves moving the sample closer to the X-ray source, but the sample must still be able to make a full rotation, which is practically not possible when doing a 10 µm scan of the horn. The horn was in this example sectioned and removed from the chameleon, and mounted in florist foam on top of a glass rod (Fig. 2c). The 10 µm scan was done in a nano-CT instrument rather than the micro-CT. The reason for this is that although both systems overlap in their abilities to scan objects from 5 – 30 µm, typically any sample smaller than 5-10 mm wide, produces better image quality from the nano-CT instrument. The improvement comes mainly from the stabilisation of the air-bearing rotation stage, but in general the system is best suited for high resolution objects and produces sharper images for fine structures. It is similar to most benchtop micro-CT systems, which is well known to biologists in general due to their wide availability. The result of this horn-only scan is shown in Fig. 11 in a side-slice image. This clearly shows an increased image quality and resolution allowing a detailed analysis of the microstructure of the entire horn. Such analysis is not attempted in the scope of this paper, but the data is included for interested researchers to make use of.

Insert Figure 11 approximately here

Although Fig. 11 shows the horn's internal bony microstructure in great detail, an even higher resolution scan of the tip of this structure was done at 4 µm as a demonstration of the capabilities of the technique, and in such a way that the width of the sample fits the detector. The resulting CT slice image in Fig. 12a shows great detail with some indication of black spots in the dense bony structure, indicating the presence of osteocytes. The structure is visualised in 3D as well in Fig. 12b.

Insert Figure 12 approximately here

Since the detail of Fig. 12a shows signs of osteocytes within the bony structure, a sub-micron scan was attempted of this material. This required further sectioning, to allow a full field of view of approximately 1.5 mm only. The resulting scan at 950 nm (0.95 µm) is shown in Fig. 13a in a 2D slice image which clearly resolves the osteocytes and in Fig. 13b showing the osteocytes in 3D with a colour-coded volume analysis. This sequence of scans indicates the capabilities of multiple resolution scanning, providing different types of information and allowing different types of image analysis. Data sets are included and researchers are urged to make use of these.

Insert Figure 13 approximately here

4. Summary

3D laboratory X-ray micro-CT is a fast growing non-destructive testing and analysis method in scientific research applications. The increasing accessibility of such instruments will lead to an increasing number of new discoveries in scientific research applications in biological sciences in particular. The aim of this paper was to provide a focused "how-to" guide for new potential users to better plan their work, and understand how to best make use of this technology. A specific new case study was used as demonstration – the Jackson's three-horned chameleon specimen which was scanned at different settings, and the full data sets provided as supplementary information. These data sets are meant to be used to gain a better understanding for viewing and handling typical 3D data sets from the technique. An interesting feature related to the tongue of the animal was segmented in 3D and found to be extremely straight, and the skeletal structure was demonstrated by simple thresholding. Scans up to submicron resolution resolved osteocyte structure in the horn of the chameleon. These observations demonstrate a typical multiscale investigation by X-ray micro- and nano-CT, which usually lead to exciting new observations in three dimensions.

5. Acknowledgements

The Botany and Zoology Department at Stellenbosch University is thanked for lending the chameleon specimen used in this study.

6. References

1. Kalender WA. X-ray computed tomography. Physics in Medicine and Biology. 2006;51(13):29-43

2. Brunke O. High-resolution CT-based defect analysis and dimensional measurement. Insight-Non-Destructive Testing and Condition Monitoring. 2010;52(2):91-3

3. Singhal A, Grande JC, Zhou Z. Micro/Nano CT for visualization of internal structures. Microscopy Today. 2013;21(02):16-22

4. Mizutani R, Suzuki Y. X-ray microtomography in biology. Micron. 2012;43(2):104-15

5. Schoeman L, Williams P, du Plessis A, Manley M. X-ray micro-computed tomography (μCT) for non-destructive characterisation of food microstructure. Trends in Food Science & Technology. 2016;47:10-24

6. Cnudde V, Boone MN. High-resolution X-ray computed tomography in geosciences: A review of the current technology and applications. Earth-Science Reviews. 2013;123:1-17

7. Maire E, Withers PJ. Quantitative X-ray tomography. International Materials Reviews. 2014;59(1):1-43

8. Landis EN, Keane DT. X-ray microtomography. Materials characterization. 2010;61(12):1305-16

9. Stauber M, Müller R. Micro-computed tomography: a method for the non-destructive evaluation of the three-dimensional structure of biological specimens. Osteoporosis: Methods and Protocols. 2008:273-92

10. Kotwaliwale N, Singh K, Kalne A, Jha SN, Seth N, Kar A. X-ray imaging methods for internal quality evaluation of agricultural produce. Journal of Food Science and Technology. 2011;51(1):1-15

11. Duliu OG. Computer axial tomography in geosciences: an overview. Earth-Science Reviews. 1999;48(4):265-81

12. Kak AC, Slaney M. Principles of computerized tomographic imaging New York: IEEE Press; 1988.

13. Lin C, Miller J. Cone beam X-ray microtomography-a new facility for three-dimensional analysis of multiphase materials. Minerals and Metallurgical Processing. 2002;19(2):65-71

14. Hanke R, Fuchs T, Uhlmann N. X-ray based methods for non-destructive testing and material characterization. Nuclear Instruments and Methods in Physics Research Section A: Accelerators, Spectrometers, Detectors and Associated Equipment. 2008;591(1):14-8

15. Feldkamp L, Davis L, Kress J. Practical cone-beam algorithm. JOSA A. 1984;1(6):612-9

16. Donis-González IR, Guyer DE, Pease A, Barthel F. Internal characterisation of fresh agricultural products using traditional and ultrafast electron beam X-ray computed tomography imaging. Biosystems Engineering. 2014;117:104-13

17. Matthews T, Du Plessis A. Using X-ray computed tomography analysis tools to compare the skeletal element morphology of fossil and modern frog (Anura) species. Palaeontologia Electronica 2016;19(1): 1-46

18. Liu Y, Kiss AM, Larsson DH, Yang F, Pianetta P. To get the most out of high resolution X-ray tomography: A review of the post-reconstruction analysis. Spectrochimica Acta Part B: Atomic Spectroscopy. 2016;(117):29-41

Tables:

Table 1. Summary of faulty or problematic CT scans as discussed throughout this paper, stating problems, causes and possible solutions, respectively.

Problem	Cause	Solution
Grainy image	Image acquisition time too low	Increase image acquisition time
Streaky artifacts	Differences in absorption from different angles; X-ray penetration is insufficient	Increase voltage
Poor contrast	Too high voltage is used	Reduce voltage
Blurred image	Improper sample mounting; allowing sample to move during scanning	Proper mounting to ensure no movement during scanning
Stitching artifacts / vertical or horizontal line	Sample too wide for a single scan	Make sub-sections of sample; use a smaller sample or less magnification
Beam hardening / cupping effect	Edge of sample seems brighter than the inside of the sample due to insufficient penetration of the sample	Reconstruction: use beam hardening correction option, or scan with higher voltage and more beam filters
Small movement or shift (double edge)	Inaccuracy of rotation stage or movement of sample	Reconstruction: do an offset correction; or rescan if offset cannot be corrected. Reset stages. Hardware could be faulty, e.g. tilt axis alignment
The histogram is shifted strongly to one side	Small quantity of bright dense phase are present, but irrelevant	Reconstruction: make use of the clamping option
Scattering	Causes brighter or darker projection images from different angles	Reconstruction: select background detector counts in each image and normalise across the series of images
Ring artifacts	Bright rings are visible in the top slice view	Reconstruction: make use of ring artifact reduction by disregarding 'dead' pixels from the projection image (or disregard pixels in the acquisition process)

Central rotation artifact	The center of rotation is visible as a line in a side slice view, or a dot with concentric rings from the top view.	Make use of detector shift option in acquisition, which smooths out the artifact.
Bright ring around outside of scan volume, resulting in poor image quality	In ROI scans where the sample extends over the side of the 2D image	Use special reconstruction algorithm which corrects for this, or crop the ROI further in reconstruction
Cone beam artifacts	Affecting the edges of materials near the edges of the detector	Use less magnification to fill less pixels on detector

Figure captions:

Figure 1. The fundamental components of a micro-CT instrument.

Figure 2. Different mounting styles are depicted in (a) where the whole chameleon is wrapped in cloth for wet scanning, (b) the whole chameleon is mounted upright in coke bottle for vertical multiple scanning and (c) a nano-CT sample mount of a 1.5 mm section of the chameleon's horn.

Figure 3. A graph of the typical resolution obtained when working with different sample sizes.

Figure 4. Micro-CT slice images of the chameleon, where (a) a metal tag is included in the scan volume, resulting in streaky artifacts, (b) too low voltage was used and therefore image artifacts are found around dense parts of chameleon and (c) too high voltage was used, showing poor contrast.

Figure 5. Micro-CT slice images of the chameleon, showing poor images caused by (a) reconstruction clamping set too high, resulting in poor contrast, (b) double edges due to incorrect offset calculations during reconstruction, and (c) slight blur due to tilt-axis misalignment.

Figure 6. A step-wise guide for micro-CT scanning and data analysis, featuring settings, considerations, guidelines and options related to micro-CT imaging and analysis of biological sample.

Figure 7. Images demonstrating the mounting of the three-horned chameleon with (a) showing the florist foam mounting material that forms the basis on which the specimen is placed, and (b) the X-ray projection image showing the very low density of the mounting material.

Figure 8. 3D reconstructions of the three-horned chameleon with a (a) surface view and (b) a semi-transparent view showing the dense skeleton structure in yellow.

Figure 9. (a) An isolated bony feature related to the tongue of the animal is shown in red in a 3D view, with (b) showing this feature in a CT slice image.

Figure 10. A high resolution ROI scan (30 µm) of the head of the three-horned chameleon with (a) a 3D surface view, (b) a skeleton view and (c) a cropped skeleton view.

Figure 11. Nano-CT slice image of the horn of the chameleon at 10 µm, illustrating sectioning.

Figure 12. (a) Nano-CT slice image showing in great detail (4 µm) the dense structure of the bone inside the chameleon horn, with (b) visualizing the bone structure in 3D.

Figure 13. (a) Nano-CT scan revealing osteocyte distribution in the tip of the chameleon horn's internal bony microstructure at sub-micron resolution of 0.95 µm, with (b) showing the osteocytes in 3D along with a colour-coded volume analysis.

Figure

Click here to download Figure Fig 1.pdf

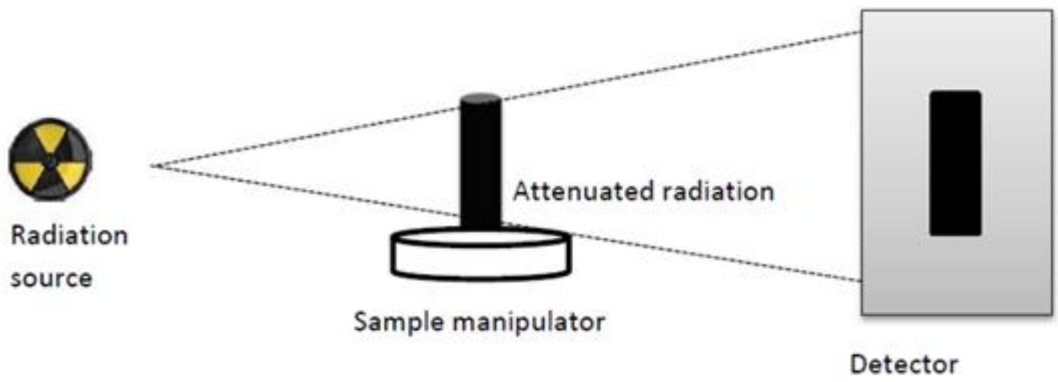

Figure
Click here to download Figure Fig 2.pdf

(a)

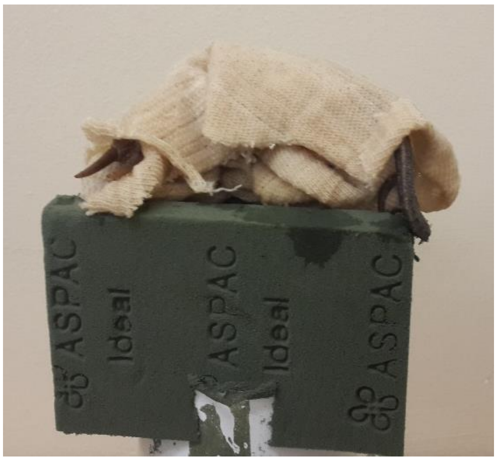

(b)

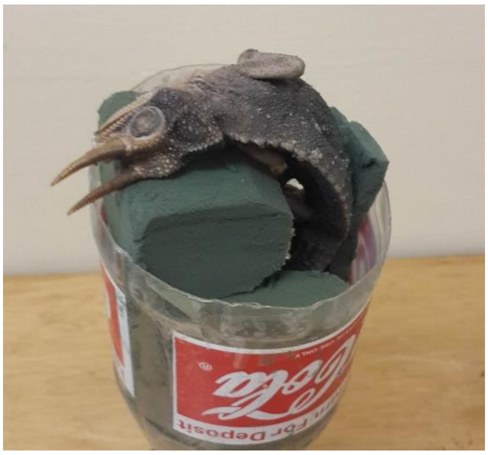

(c)

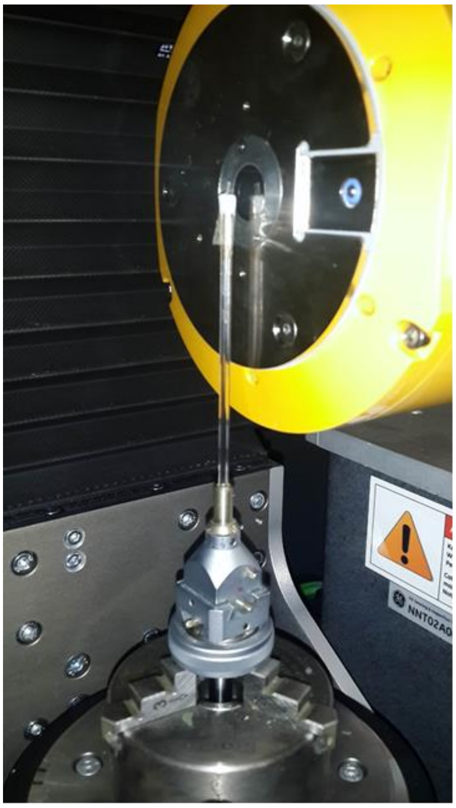

Figure Click here to download Figure Fig 3.pdf

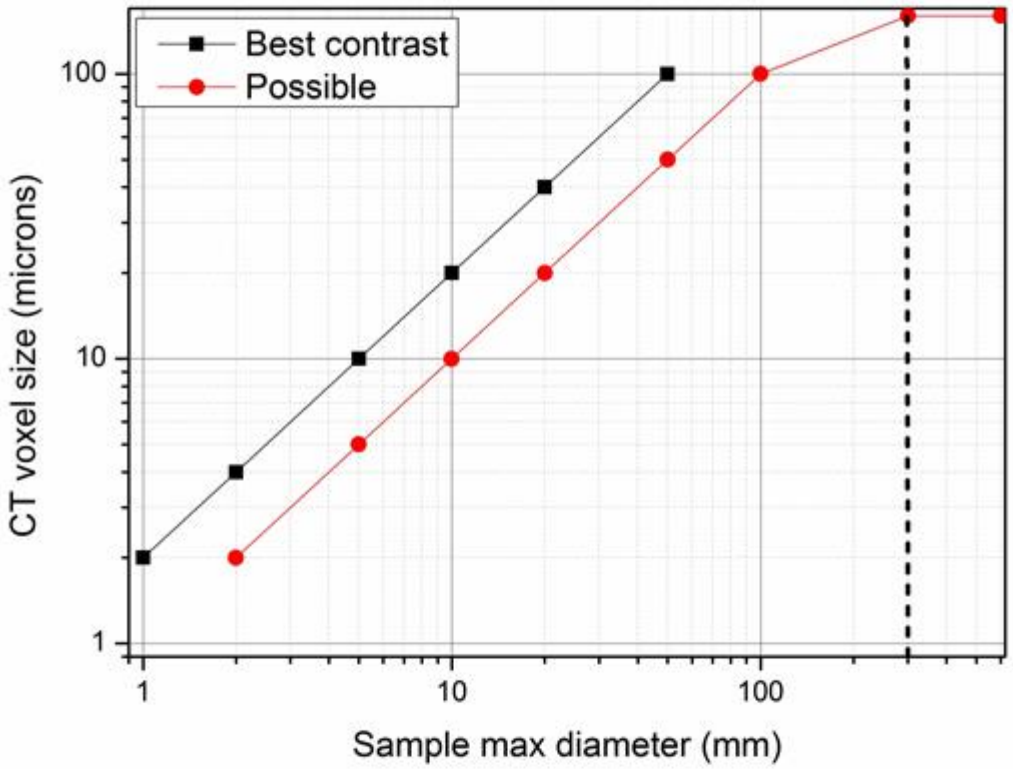

Figure
Click here to download Figure Fig 4.pdf

(a)

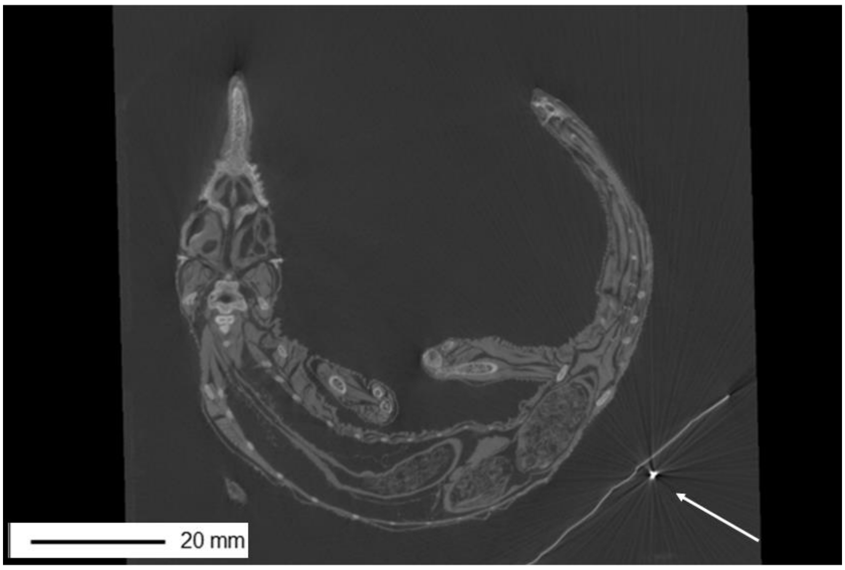

(b)

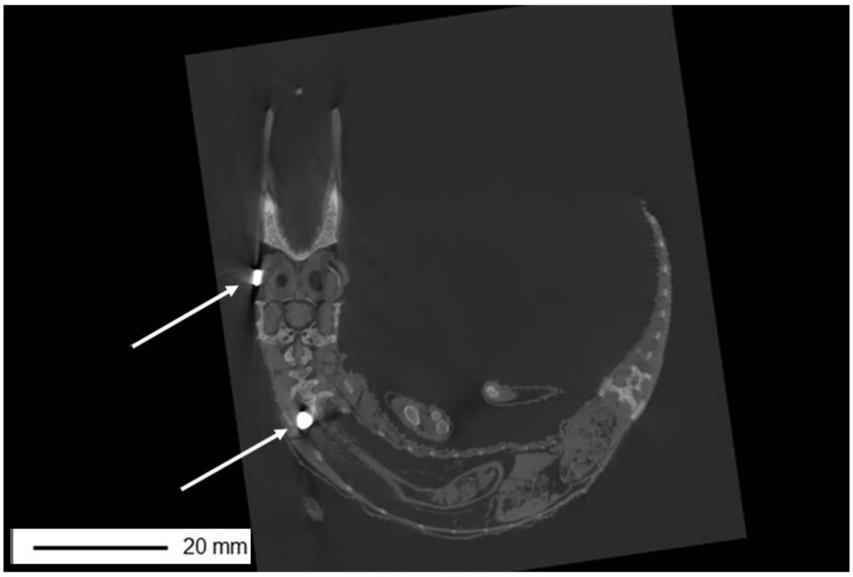

(c)

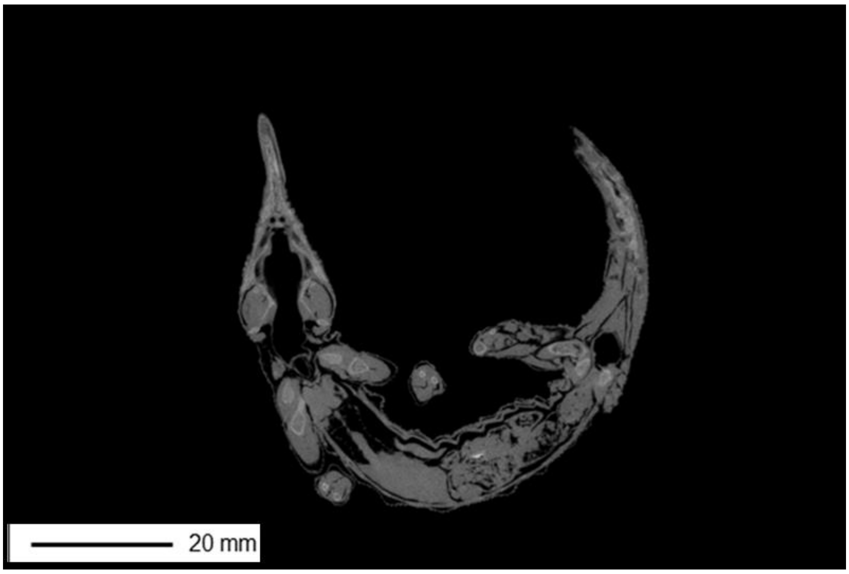

Figure Click here to download Figure Fig 5.pdf

(a)

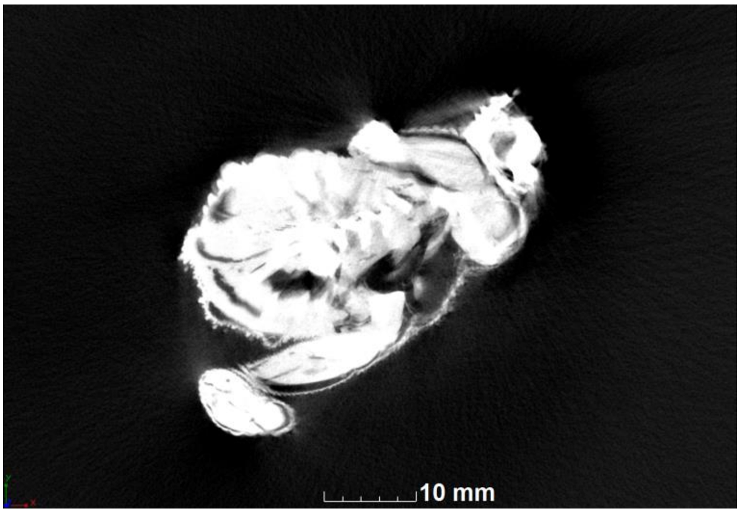

(b)

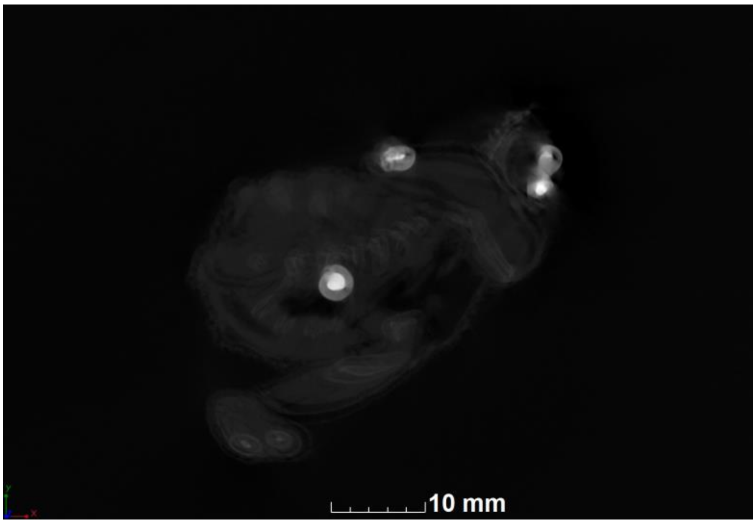

(c)

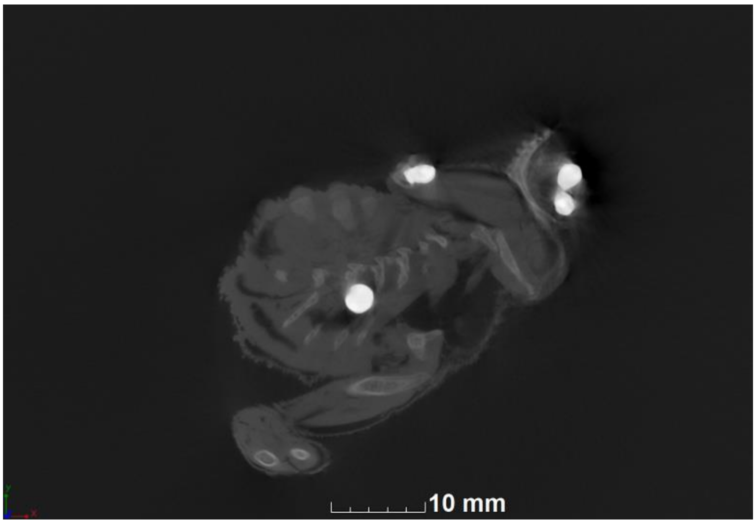

Figure Click here to download Figure Fig 6.pdf

1. CT Set-up parameters

- Sample preparation and mounting (Figure 7)
- Considering size for correct resolution setting: *Guideline I*
- Chosing step positions and averaging: *Guideline II*
- Chosing voltage settings: *Guideline III*
- Choice of filtration of beam and detector
- Calculating penetration ratio: *Guideline IV*

2. Scanning

- Normalising / correcting the back ground
- Run a beam centering
- Load sample and commence scanning
- Monitor the process

3. Reconstruction

- Cropping the field of view
- Choice of data type
- Offset correction
- Beam hardening correction
- Correct for variation in intensity - normalisation step

4. Visualisation (Figure 8)

- Thresholding function
- Removal of mounting material
- Smoothing
- Surface rendering

5. Image processing and analysis

- Segmentation: Thresholding, region growing, drawing tool (Figure 9)
- Smoothing using filters
- Image analysis: length & width measurements, surface area analysis, volume analysis

6. Further scanning at higher resolution

- Close-up scans:
  - head: 30 µm ROI scan (Figure 10)
    - horn: 10 µm sectioning scan (Figure 11)
      - tip of horn: 4 µm sectioning scan (Figure 12)
        - tip of horn: 0.95 µm sectioning scan (Figure 13)

 Click here to download Figure Fig 7.pdf

(a)

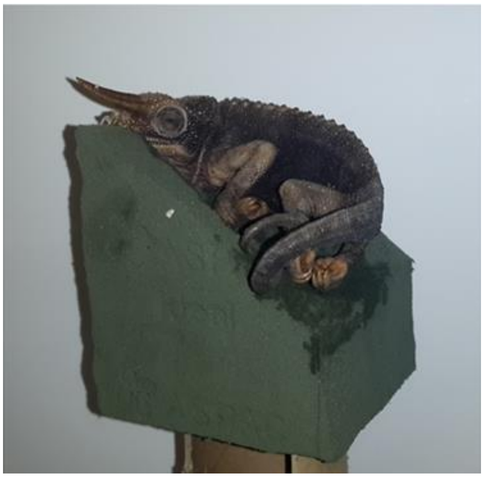

(b)

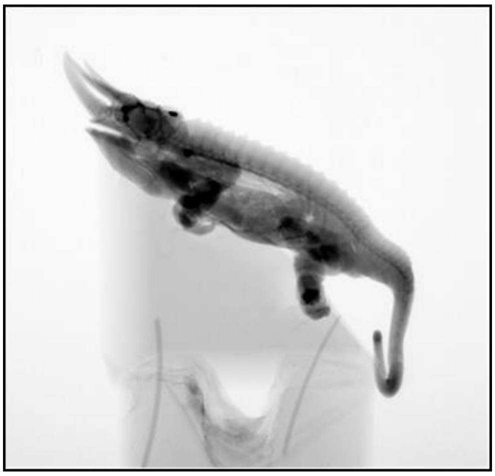

Figure Click here to download Figure Fig 8.pdf

(a)

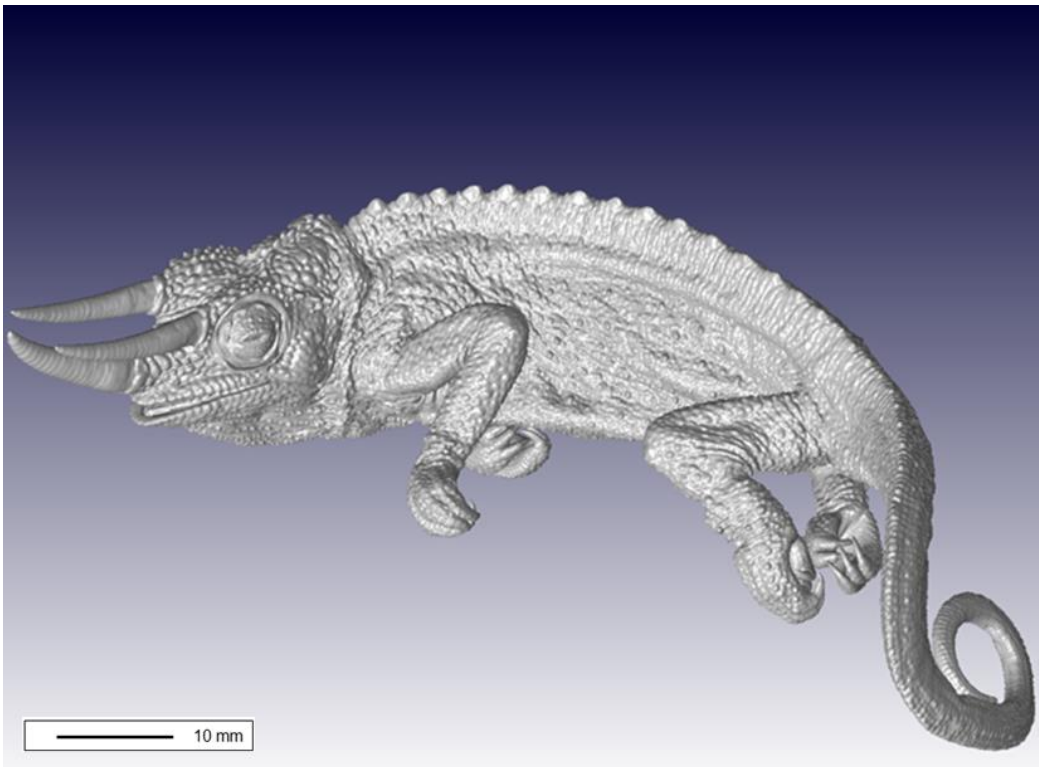

(b)

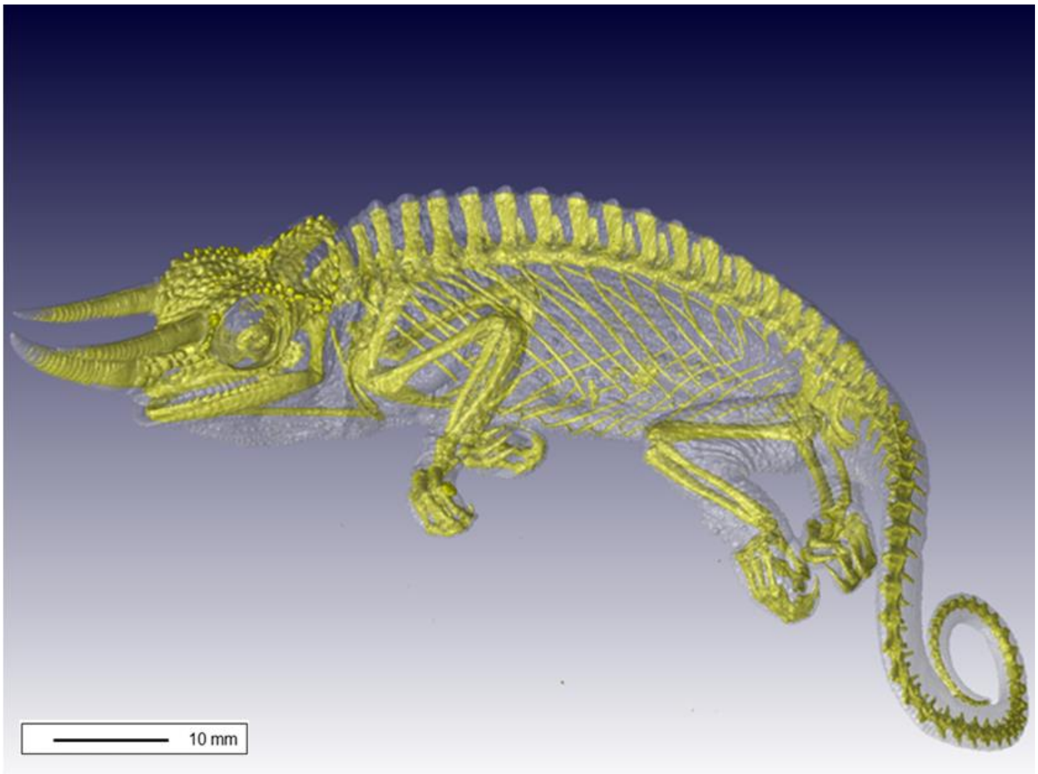

 Click here to download Figure Fig 9.pdf

(a)

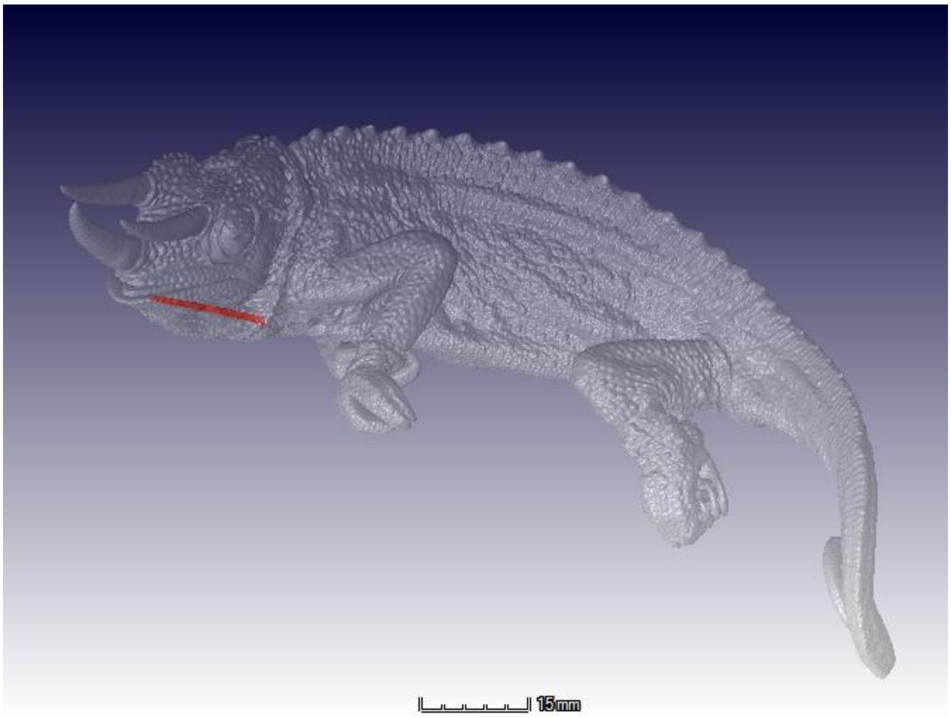

(b)

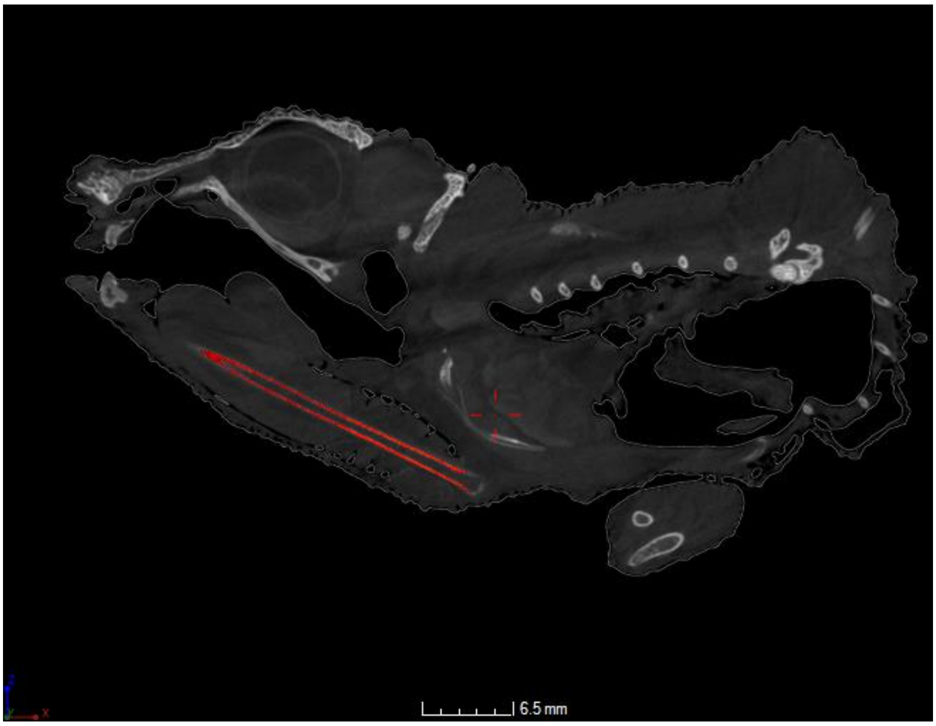

Figure Click here to download Figure Fig 10.pdf

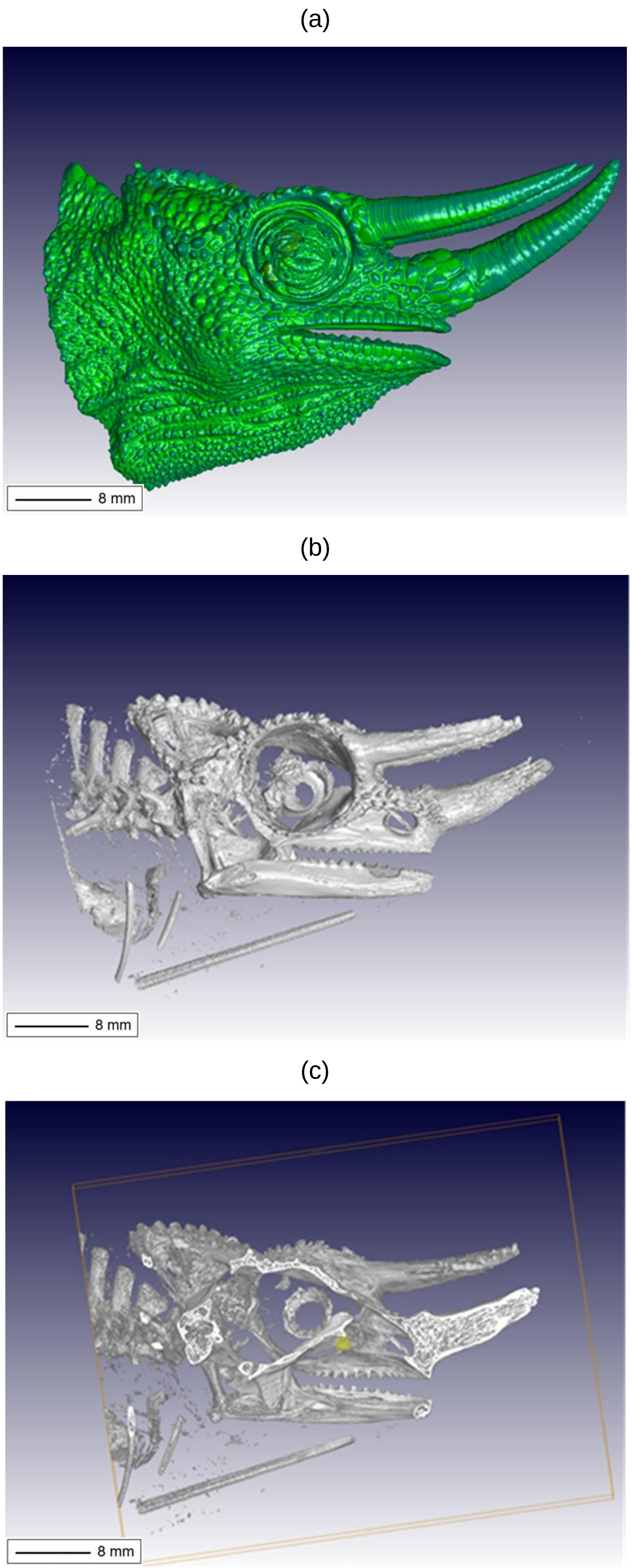

 Click here to download Figure Fig 11.pdf

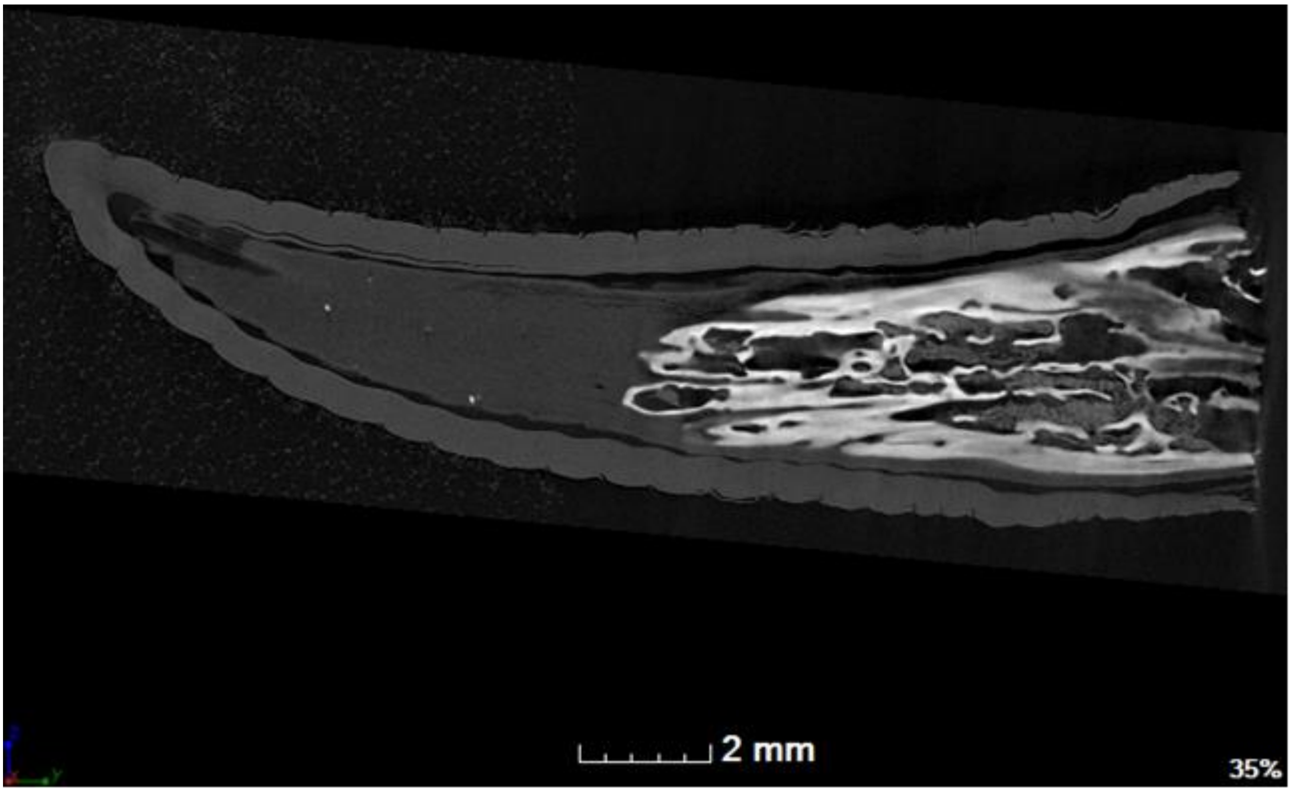

 Click here to download Figure Fig 12.pdf

(a)

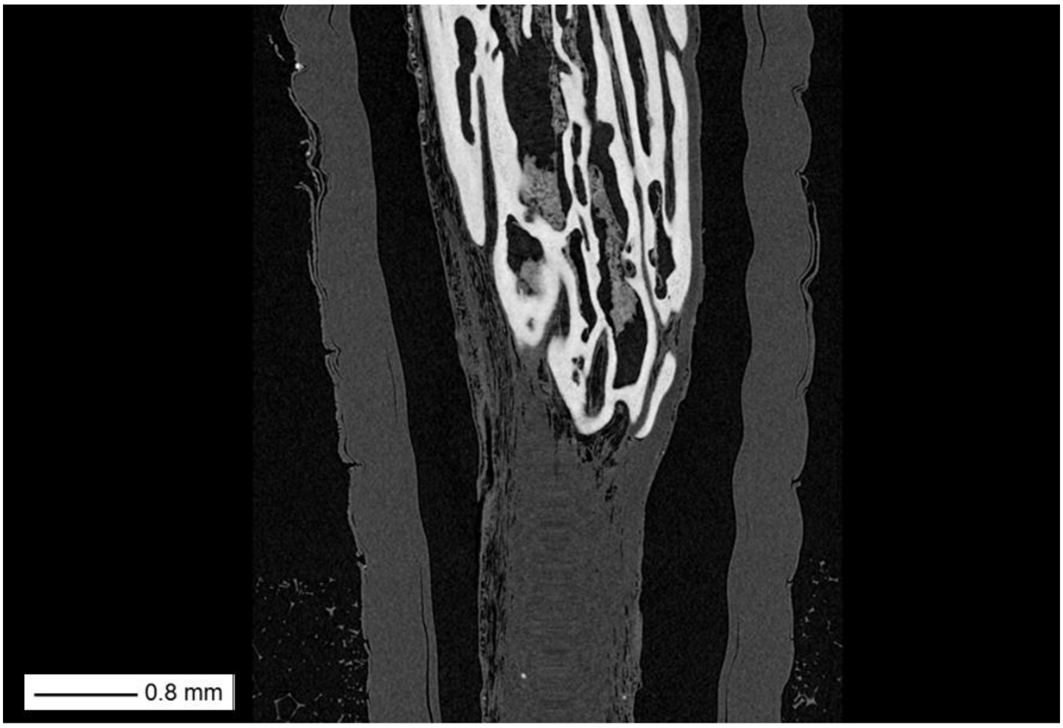

(b)

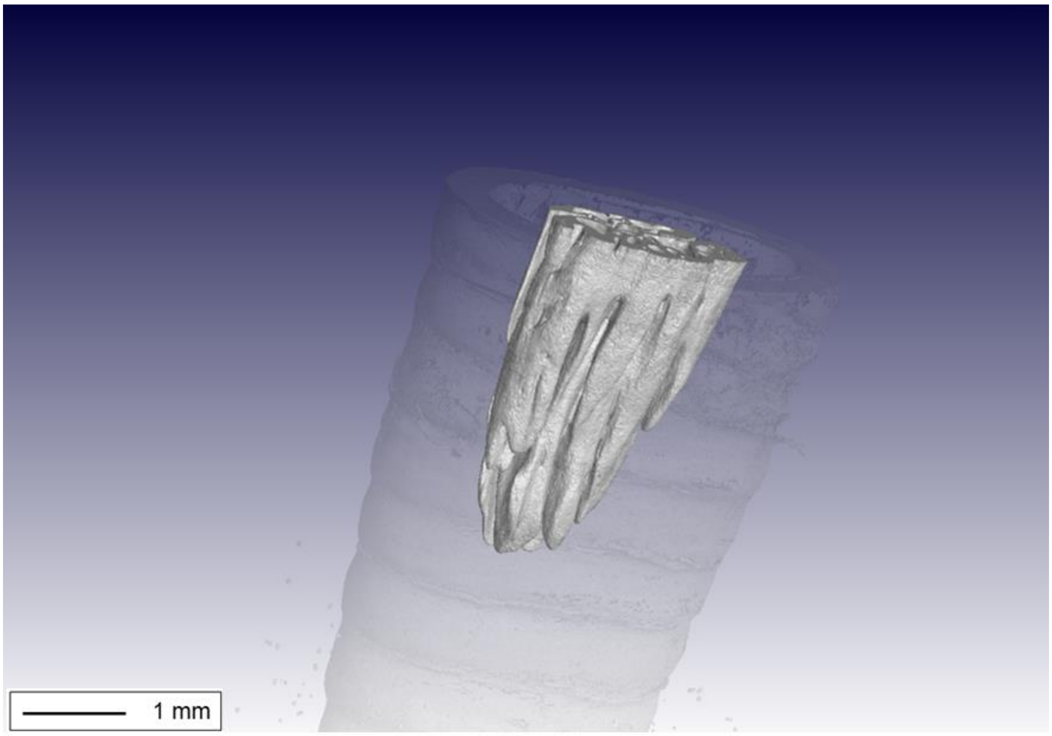

Figure

Click here to download Figure Fig 13.pdf

(a)

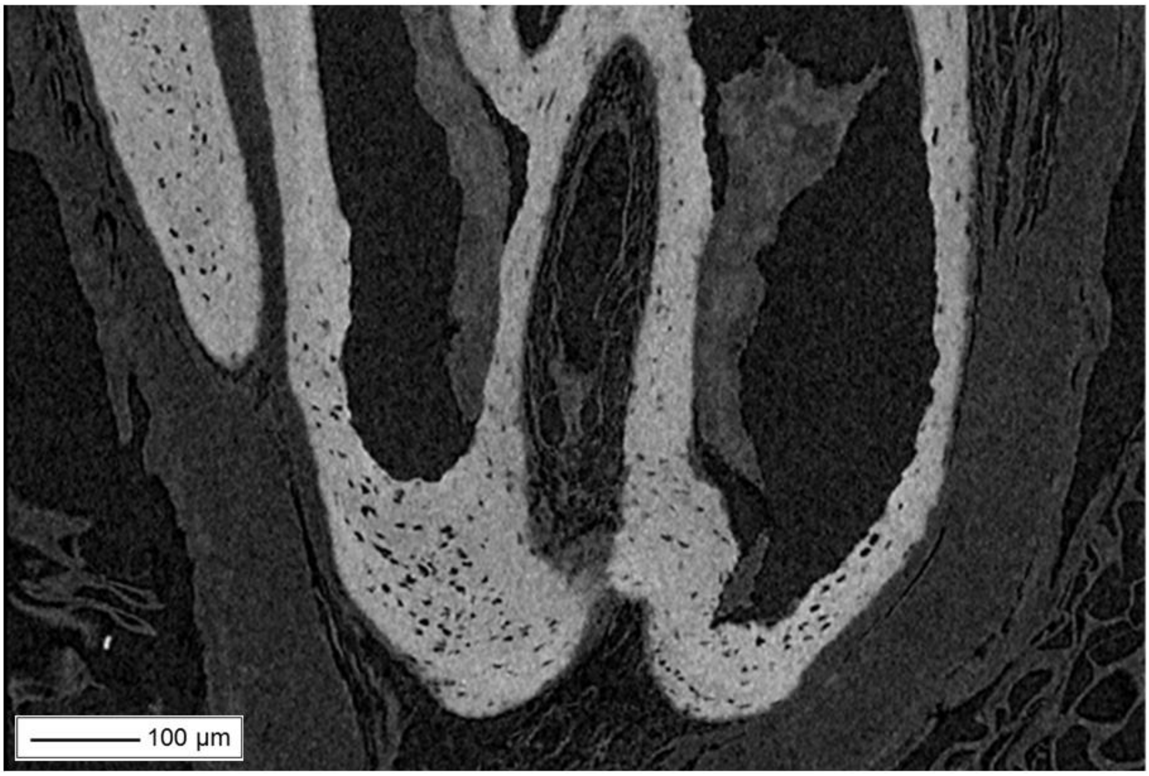

(b)

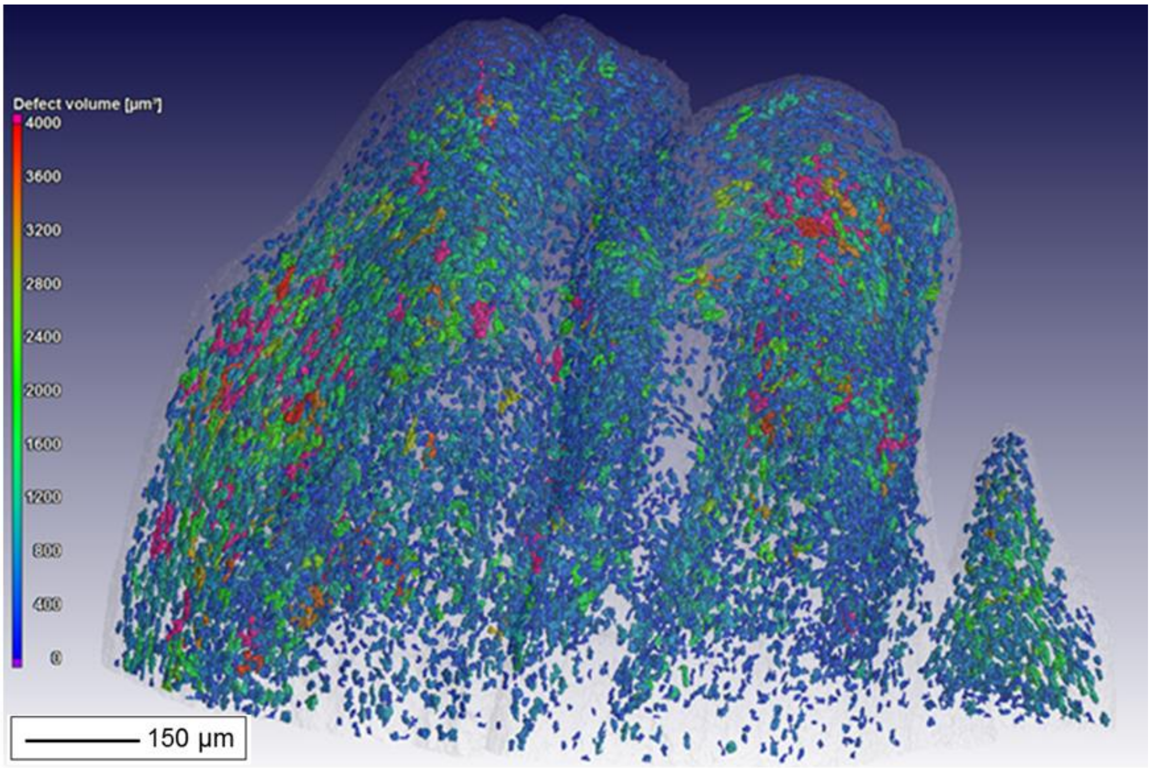

Click here to download Personal Cover Cover Letter_GigaScience.doc.docx

7 June 2016

Dr Scott Edmunds

Editor

GigaScience

Dear Dr Edmunds

Re: Resubmission of manuscript GIGA-D-16-00019 for GigaScience

We hereby re-submit our manuscript titled: **Laboratory X-ray micro-computed tomography: a generalised approach for biological samples using a three-horned chameleon as example** (previous title: X-ray microtomography at Stellenbosch University: a new users guide). This new submission has been considerably reworked thanks to valuable comments from the reviewers. We added an author who assisted significantly in the rework process.

We restructured the manuscript to remove all reference to our facility and specific manufacturers in order to generalise the guidelines provided. We used the chameleon as example to walk the reader through the entire process, demonstrating the application of guidelines provided. Our focus was on providing solutions to possible problems and included examples to demonstrate some of these. We hope this now caters for non-experts and experienced users alike. We also added new data sets of higher resolution scans of the horn of the chameleon to demonstrate the capabilities of multi-scale CT and nano-CT. More details of changes made during the rework process is described point-by-point in response to reviewer comments below.

Reviewer #1:

1. The authors removed any information specific to the Stellenbosch CT facility and focussed on micro-CT facilities in general.

   Specifically:

   - we added information on exposure time, filters, samples size and material and other factors related (see Sections 3.1.2 to 3.1.6 (pp. 5 – 10) in the revised manuscript). We added examples of good / bad projection images throughout the revised manuscript, adding figures (refer to Figs 4 and 5 in the revised manuscript) and also summarised these examples in a Table (see Table 1 (pp.

2 – 25) in revised manuscript), including the causes and problem solving options.

- p.4, line 1 (now p. 7, line 251) was changed to include the words “if available” and now reads “…it is advisable to use the skip function, if available, which ignores the first image acquired at each new step…”
- All topics covering resolution, sample size, detector size etc. were changed to supply general descriptions that can be useful to any user at any facility (see Section 3.1 (pp. 4 – 10) in the revised manuscript).
- P. 5, line 30 has been changed and now covers two sub-sections (see Section 3.1.3 (pp. 6 – 7, lines 211 – 231) and Section 3.1.5 (pp. 8 – 10, lines 268 – 334), in particular lines 305 – 308 in the revised manuscript) with more general explanations and guidelines. With regards to the comments about the filters and their placement, please see Section 3.1.5 p. 9, lines 310 – 319 in the revised manuscript that addresses this.
- P. 5, line 47 and following has been changed and now covers a section dedicated to the relationship between voltage and current. Please see Section 3.1.5 (pp. 8 – 10, lines 268 – 335) in the revised manuscript.
- P. 6, paragraph 2.3 has been rewritten and is now presented as Section 3.1.1 (pp. 4 – 5) with additional figures (Figs. 2a, b and c) in the revised manuscript, covering aspects of mounting. Artifacts related to sample mounting is presented in Table 1 (pp. 23 -24), although other artifacts are covered in Section 3.1.6 (p.10, lines 337 – 359) in the revised manuscript.
- P. 6, paragraph 2.4 was revised in that any information relating to the Stellenbosch facility was removed, and a new section was added (see Section 3.3.2 Data output styles (pp. 13 – 14, lines 463 – 495) in the revised manuscript), covering different output types.
- P. 6, paragraph 2.5 was changed in order not to convey any lab-specific information, but rather incorporate a list of commonly used software packages. Please refer to Section 3.4 Visualisation (p. 14, lines 497 – 508) in the revised manuscript. It was also attempted to explain volume rendering, surface modelling, as well as CAD, also giving the full wording for the acronym CAD – please see Section 3.4 Visualisation (p. 14, lines 497 – 508) in the revised manuscript.
- P. 7, paragraph starting with line 17 has been changed completely and is now presented as Section 3.5 Image processing and analysis (pp. 14 – 16, lines

510 – 570) in the new manuscript. References (Matthews & Du Plessis, Singhal et al., Schoeman et al. and Liu et al.) has been added.

- P. 7, line 39 has been removed.
- P. 7, section 3 was removed from the manuscript.
- P. 8, section 4 has been changed following the first approach as suggested by the reviewer. The chameleon example has been used to demonstrate all the relevant steps that needs to be followed when doing micro-CT scanning. A guide has also been presented in the form of a flow diagram. Please refer to Section 3.8 Micro-CT scanning of a three-horned chameleon – an example of data acquisition and analysis (pp. 18 – 21, lines 632 – 761), Fig. 6 (flow diagram) and figures relating to the scans (Figs. 7 – 13) in the revised manuscript. Additional multi-scale X-ray analysis were added of the structure of the chameleon's horn.
- Section 5 was removed from the manuscript.
- P.16, line 11 and following was removed from the manuscript.

2. As suggested by the reviewer, schematic illustrations of concepts, illustrations of scanned images showing the effect of various settings and flow diagrams were included in the manuscript.
    - These are found as Fig. 1, a schematic illustration of the fundamental components of a micro-CT instrument, Fig. 2 illustrating different mounting styles, Figs. 4 and 5 illustrating examples of poor images and relevant artifacts, and Fig. 6 being a flow diagram or a step-wise guide for micro-CT scanning, applied on the chameleon example. Fig. 3 has also been included in the revised manuscript and is a graph of the typical resolution obtained when working with different sample sizes, thereby explaining a rather complex concept, visually.
    - A comment was raised about figure legends that should describe whether volume or surface rendering was used. As only surface rendering was used in all the images, the authors did not include this into the legends.
    - Everything regarding 3D printing was removed from the manuscript.

3. To address this comment of the reviewer, the authors decided that the paper should be accessible to established as well as new CT users. More detail was thus added on all relevant topics, including scan works, reconstruction, image processing and analysis.

4. Acronyms were all given in full before being abbreviate.

5. The species name was corrected (See p. 4, line 116 in the revised manuscript).

Reviewer #2:

General comments:

- All referencing towards the CT facility at Stellenbosch University was removed.
- The paper was revised to focus on micro-CT facilities in general and also solely on the analysis of biological samples.
- Soft tissue staining was addressed in Section 3.1.1 p. 4, lines 132 – 138 in the revised manuscript.
- Synchrotron CT differences were addressed on p. 2, lines 56 – 61 in the revised manuscript.
- The question relating voxel and spatial resolution was addressed in the newly added Section 3.1.3 pp. 6 – 7, lines 211 - 226 in the revised manuscript.
- A section on artifacts was provided in Section 3.1.6, p. 10, lines 336 – 358, also included in a table, Table 1 and figures, Figs. 4 and 5 in the revised manuscript.
- Detector size was addressed in the newly added section Section 3.1.4, pp. 6 – 7, lines 211 – 232 in the revise manuscript.
- Maintenance issues were addressed in Section 3.7, pp. 17 – 18, lines 603 – 631 in the revised manuscript.
- Overnight scanning were also addressed in Section 3.7, pp. 17 – 18, lines 603 – 631 in the revised manuscript.
- Fast scout scans were addressed on p. 11, lines 376 – 381, as well as p. 15, lines 528 – 530 in the revised manuscript.
- Filters were include in the newly added Section 3.1.5 p. 9, lines 310 – 319 in the revised manuscript.
- Numerous relations with regards to resolution was included in Sections 3.1.2 and 3.1.3 (pp. 5 – 7, lines 211 – 232) in the revised manuscript.
- An overview on mounting techniques were added in Section 3.1.1 Sample preparation and mounting, pp. 4 – 5, lines 130 – 174, including Figs. 2a, b and c in the revised manuscript.
- Sample preparation was addressed in Section 3.1.1 Sample preparation and mounting, pp. 4 – 5, lines 130 – 174, in the revised manuscript.
- Signal-to-noise ratio was further explained in terms of resolution in Section 3.6, pp. 16 - 17, lines 573 – 593 in the revised manuscript.

- Datasets were further explained in Section 3.3.2, p. 13 – 14, lines 463 – 495 in the revised manuscript, covering explanations on data volume.
- Data formats were also covered in the newly added Section 3.3.2, p. 13 – 14, lines 463 – 495, in the revised manuscript.
- The addition of scales to stacks and the calibration thereof has been covered in Section 3.3.2, p.14, lines 487 – 495, in the revised manuscript.
- Open source software packages, amongst others, were included in Section 3.4, p. 14, lines 497 – 508 in the revised manuscript.
- The chameleon example was used for both micro-CT and nano-CT scans and illustrated the differences between the two systems. Please refer to Section 3.8 Micro-CT scanning of a three-horned chameleon – an example of data acquisition and analysis, pp. 18 – 21, lines 632 – 761 in the revised manuscript.
- The differences between 8 and 16-bit files were explained in Section 3.3.2 Data output types, p.13, lines 463 – 475 in the revised manuscript.
- The calibrated status of image stacks has been addressed in Section 3.3.1, p. 14, lines 487 – 495 in the revised manuscript.
- The line that mentions “can take up to 4 weeks” were deleted from the manuscript.
- The reviewer are thanked for introducing the authors to Stauber & Muller. This reference was added to line 75, p. 3.

Specific comments:

- Section 3, as well as Table 1 was removed from the manuscript.
- Sections 5.2, 5.5, 5.6 and 5.10 were removed from the manuscript.
- A figure (Fig. 7) was added to illustrate the mounting of the chameleon.
- Collection number of the chameleon was added on p.4, line 116 in the revised manuscript.
- Proper figure plates were created for all the figures in the paper
- All the references were revised and improper updates (DOI) were removed.
- All unintroduced abbreviations have been addressed by stating what the abbreviation or acronym stands for. CAT scan has been properly introduced on p. 2, line 30 in the revised manuscript.
- All references to TIFF have been corrected.
- The species name has been corrected as can be seen on p. 4, line 116 in the revised manuscript.

I would like to confirm that all co-authors have reviewed this latest manuscript and that they approve submission of this manuscript to GigaScience in its current format. This manuscript has not being submitted elsewhere.

I trust that you would find the manuscript in order and I look forward to hearing from you soon.

Kind regards
Anton du Plessis
