# Supplementary material for: Laboratory x-ray micro-computed tomography: a user guideline for biological samples
Source: Gigascience. 2017 Apr 17;6(6):1–11. doi: 10.1093/gigascience/gix027 (PMC5449646; doi:10.1093/gigascience/gix027)
Supplement: GIGA-D-16-00031_Revision_1.pdf [file gix027_GIGA-D-16-00031_Revision_1.pdf]

Manuscript

Click here to view linked References

Click here to download Manuscript Manuscript for submission 22 July.doc

Laboratory X-ray micro-computed tomography: a user guideline for biological samples

Anton du Plessis[1,2, a *], Anina Guelpa[1,b] & Stephan Gerhard le Roux[1,c]

[1] CT Scanner Facility, Central Analytical Facilities, Stellenbosch University, Stellenbosch, South Africa

[2] Physics Department, Stellenbosch University, Stellenbosch, South Africa

[a] anton2@sun.ac.za , [b]aninag@sun.ac.za, [c] lerouxsg@sun.ac.za

* Corresponding author

Abstract

This paper provides a detailed "how-to guide", describing many important concepts for users of laboratory X-ray micro-computed tomography (micro-CT) facilities. An introduction to micro-CT is given, followed by a background to computed tomography (CT), as well as relevant set-up, scanning, reconstructing and visualisation considerations. A three-horned Jackson's chameleon is presented as a biological sample, scanned with both a micro-CT, as well as a nano-CT scanner. Full data sets (projections, together with image stacks) that accompany the descriptive analysis are given as supplementary information, as the aim of this paper is to provide a platform for new users to gain experience in working with typical micro-CT data. Furthermore, the technical detail and discussion is relevant to all commercial types of micro-CT instruments.

Keywords: X-ray tomography, micro-computed tomography, nano-computed tomography, 3D imaging, non-destructive analysis

1. Introduction

The ability to perform non-invasive analysis is often of prime concern when working with biological samples. Therefore, much attention has been given to finding techniques for the inspection of internal properties or the evaluation of quality attributes from biological samples, non-destructively. One such technique, computerised axial tomography (CAT) or computed tomography (CT), is widely used for non-destructive imaging of the internal organs and structures of the human body [1]. This method involves the recording of two-dimensional (2D) X-ray images from various angles around an object, followed by digital three-dimensional (3D) reconstruction. Thereby, the data from CT results in a virtual rendering of the object under investigation. This allows one to virtually travel through the volume in any direction and angle, view selected internal features, make dimensional, volumetric or other more advanced three dimensional measurements [2, 3].

Industrial X-ray computed tomography is a specialised form of CT scanning, meant specifically for non-medical applications (hence the term industrial) and frequently involves resolutions in the micro-meter scale. The method is therefore termed micro-computed tomography (micro-CT) and in the case of sub-micron resolution, such methods are termed nano-CT or sometimes X-ray microscopy, as the resolution is similar to optical microscopes. Other terms used are µ (XCT), industrial CT, µCT and laboratory X-ray tomography. Industrial CT differs from medical CT in a couple of ways: the resolution is potentially better in micro-CT systems, as well as having the source and detector fixed and stable with the sample rotating (in contrast to medical CT where the sample is stationary and the source and detector move around it) and industrial CT vary greatly with regards to voltages, currents and beam filtration. Micro-CT systems can also vary considerably in system capabilities, from small low-cost benchtop systems to cabinet systems able to house larger samples and even as large as walk-in cabinet systems with multiple X-ray sources. Also, different manufacturers provide different hardware and software options. These systems should not be confused with synchrotron CT, where synchrotron radiation is the X-ray source (producing mono-energetic X-rays), as opposed to broad spectrum energies as produced by laboratory generated X-ray radiation [4, 5]. However, synchrotron CT is not as widely available or as easily accessible as laboratory CT and such work must be planned months in advance [5]. Since laboratory CT is available widely, in some cases in open access format, planning is limited to weeks or even days.

Micro-CT has numerous applications and is useful in any scientific field where non-destructive analysis is required. The versatility of this technique is shown in the number of reviews that have been published recently, such as in food sciences [6], the geosciences [7], biology [4] as well as materials sciences [8, 9]. Its potential as a tool in taxonomy has been recently demonstrated [10]. Another two good examples of micro-CT data sets are those of earthworms [11] and brittle stars [12].

Despite its numerous applications and potential, the method is still underutilised as new users struggle with the scanning process, including sample preparation, scanning, reconstruction and choices for analysis types. Lack of knowledge regarding the mentioned processes could result in poor scan quality, inefficient use of facilities or the inability to extract required information for the required research purpose. Some work has been published in a book chapter focusing on bone microstructural analysis, where details of the CT scanning process have been outlined to assist in the process, especially for new users to the technique [13]. A similar work was presented by Mizutani and Suziki [4], where the focus was on soft tissue scanning and staining methods to enhance contrast. Additionally, Metscher's [14] and Pauwel et al.'s [15] works are considered very helpful on staining biological samples. In the present data note, we focus on new users from general biological backgrounds and present a multi-scale investigation of a three-horned chameleon specimen. We add the full data sets of the chameleon scanned at 75 µm, 30 µm, 10 µm, 4 µm and 0.95 µm, to aid new users and researchers who might not have access to funding for obtaining their own micro-CT data sets. Expectantly, this work will lead to more effective use of micro-CT facilities, through an improved understanding of the capabilities and limitations of the technique.

2. Background to computed tomography

Micro-CT as a technique makes use of information from projected 2D images as obtained by a X-ray source and a detector to investigate the internal structure of a sample [16]. The fundamental components of any micro-CT instrument are (1) penetrating ionising radiation, (2) a sample manipulator and (3) a detector [17] (Fig. 1). The basic principles of micro-CT are described in Kak and Slaney [18]. X-rays are generated using a micro-focus X-ray tube, which uses a beam of electrons accelerated by a voltage of up to 240 kV or more in a vacuum tube, and are focused onto a tungsten metal target (or other metal target material, though tungsten is most widely used). The fast moving electrons hitting a metal target material create X-rays. In a micro-focus X-ray tube the X-rays are directed through and around a sample, before being collected on a 2D X-ray detector in the form of a "shadow image", also called a projection image or radiograph [3]. The sample manipulator (or rotation table) positions the sample in the path of the radiation beam and rotates it through a specific angle (usually 180 or 360°). The detector converts the attenuated radiation, which passes through the sample along a straight line, into the 2D digital images, consisting of thousands of pixels. In this way, many hundreds or thousands of 2D projection images are recorded during the scan process. After scanning, these images are used to reconstruct a three dimensional data set by making use of filtered back-projection algorithms [19]. Effectively, every volumetric pixel (or voxel) was imaged (by 2D projections) from many angles, and the sum of its view from every angle produces a good representation of the actual X-ray density and hence brightness of that voxel [3]. Following reconstruction, data visualisation and

analysis is possible using a variety of software tools, some more complex than others. These steps are all described below with a discussion of practical considerations.

Insert Figure 1 approximately here

3. Computed tomography basics

The steps associated with micro-CT include: set-up considerations (sample preparation and mounting, sample size vs. resolution, step positions and averaging, voltage settings, filtration of beam and detector and penetration ratio), scanning, reconstruction, visualisation, image processing and analysis, as well as further scanning at higher resolution. These steps will be explained, where applicable, by means of an example: a three-horned chameleon, the Jackson's chameleon (*Trioceros jacksonii*) (Specimen number USEC/H-2927 from the Stellenbosch University Zoology/Botany Department's collection) scanned using a Phoenix V|Tome|X L240 (General Electric Sensing and Inspection Technologies / Phoenix X-ray, Wunstorff, Germany) micro-CT system, as well as a Phoenix nanotom S (General Electric Sensing and Inspection Technologies / Phoenix X-ray, Wunstorff, Germany) nano-CT system, both located at the CT Scanner Facility of the Central Analytical Facility (CAF), Stellenbosch University, South Africa.

3.1 Set-up considerations

A scan set-up involves a good choice of parameters based on some general guidelines, experience with different sample types and requirements of the analysis. Five general guidelines (Guidelines I to V), along with explanations will be presented in this section for careful consideration when performing micro-CT measurements of biological samples.

<u>3.1.1 Sample preparation and mounting</u>

Amongst analytical techniques, micro-CT requires very little, if any, sample preparation. A sample can usually be scanned exactly as provided, without any sample preparation. To obtain best scan quality it is important to load the sample properly. Samples should be loaded at a slight angle to ensure that parallel surfaces to the X-ray beam are minimised. Parallel surfaces are not penetrated properly and lead to image artifacts and lack of detail in the data set in the plane of the flat surface parallel to the beam.

The mounting involves the use of a low-density material (cardboard tubes, plastic bottles or glass rods) which holds the sample in place on a rotation stage (turntable), but separates the sample from the dense rotation stage hardware. The most important parameter for obtaining a good scan is to ensure that the sample does not move during the scan time. This is more relevant for longer scan times. If a sample suddenly moves (for example due to the mounting tape coming loose during the scan) it will result in a blurred 3D image. In the same way, a wet sample that dries out

during the scan (causing shrinking) will also cause a blur. For wet samples, such as biological preserved specimens, there are different approaches that can be used to overcome this problem. One approach is to dry the sample before scanning, or to make use of freeze-drying. Another approach is to wrap the sample in a wet cloth, thereby keeping the sample wet for an adequate period. It should, however, be noted that in particular cases samples need to remain in their ethanol filled jars or be transferred to plastic container completely filled with ethanol when being scanned. In such cases, too much damage will be inflicted on the samples during manual handling. In these cases, staining should be considered as it increases the contrast of the specimen compared to the surrounding medium [4, 15, 14]. It is also possible to scan samples inside liquid filled tubes, although it can be challenging to keep the sample in place. Additionally, if it is kept in place by the edges of the container, these edges will not be separable from the sample in the image processing steps. Another approach to minimise blurring is to use very fast scan settings, although this is not always possible when highest quality and resolution is required.

The vertical mounting method is useful when multiple-scans at higher resolution are done of different sections along the height and then stitched together to form a higher resolution complete data set. The nano-CT mounting is also used in similar form as with micro-CT of smaller samples, where a glass rod is used and the sample is mounted on top of this rod with double sided tape, glue or using a small cube of foam stuck to the rod. Other options include rigid foam stuck to the top of a glass rod, fitted with a small cavity or slit, plastic film covering soft tissue or wet samples and soft foam for wrapping a sample placed on top of the glass rod.

3.1.2 Sample size vs. resolution

Micro-CT instruments can accommodate sample sizes from as large as 40 cm (or larger in some systems) to as small as several micrometers [7]. Large walk-in cabinet systems allow the detector to back up as far as 1.6 m from the X-ray source, which allows samples as large as 40 cm to be loaded and fit within a single scan volume, with the usual single scan volume covering approximately 30 cm at its furthest position. Smaller cabinet micro-CT systems allow generally a 1 m source-detector distance, allowing up to 15 cm in a single scan. Benchtop instruments have further limitations on sample size and might require sectioning of samples in the preparation process, although this may not always be a drawback since benchtop instruments are lower cost and more readily available than larger systems.

The choice of resolution is the first major factor affecting a micro-CT scan. A useful guideline (Guideline I) when estimating the best possible resolution for a sample of known dimensions is:

i. The best commonly used resolution is a factor 1000 smaller than the width of the sample. This means for a sample 100 mm wide, the best resolution is typically 100 µm

The above guideline is based on the standard practice of using only the central 1000 of 2000 available pixels of the detector to minimise possible artifacts from the edges. This is due to two reasons: firstly, the cone beam has reduced intensity near the edges and secondly, the cone beam geometry results in non-ideal reconstruction away from the central slice. For both these reasons, it is best to use the middle of the detector to minimise artifacts and reduced contrast near the edge as compared to the middle of the detector. Most detectors have 2000 pixels, but some systems have more, which allow improved magnification for the same sample size, but might introduce other problems such as data set size and long reconstruction times. It is in theory possible to use all 2000 available pixels in the above example, in which case up to 50 µm is possible. However, besides the potential for artifacts from the edge regions, it is practically very difficult to mount a sample perfectly central on the rotation axis in order for it not to move out of the field of view during a rotation, and this process could also be very time consuming at high resolution.

This guideline is applicable to most modern micro-CT instruments, which typically have 2000 detector pixels. Resolutions of micro-CT scanners are generally in the range of 5 – 150 µm, compared to medical CT scanners having best resolutions of 70 µm. Whereas nano-CT scanners have resolutions down to 0.5 µm in laboratory instruments (some higher resolution instruments are available, but not widely available yet). But it should be noted that some instruments may have overlapping resolutions and that the terms (micro and nano) are not fixed and could differ depending on model types. Best voxel sizes for the General Electric Phoenix nanotom is 0.5 µm, the Zeiss-Xradia have models Versa (0.5 µm) and Ultra (0.05 µm) and the Bruker SkyScan 2211 is 0.1 µm. Actual resolution depends on sample size and in scan conditions, and few direct comparisons have been made between these instruments.

3.1.3 Resolution, voxel size and X-ray spot size

The voxel size of a micro-CT image is dependent on the magnification and object size as described above. This is related to the distance of the sample from the X-ray source and the detector [6]. Voxel size and spatial resolution are two concepts that are often confused, since the voxel size is the size of a pixel in 3D space, i.e. the width of one volumetric pixel (isotropic in 3 dimensions). This value does not consider the actual spatial resolution capability of the scan system. For example, if the X-ray spot size (focused X-ray spot from the source) becomes larger than the chosen voxel size, the spatial resolution of the system becomes poorer. That means less details are detectable, despite a good voxel size, due to the actual resolution which is not optimal. Since most commercial systems limit the size of the X-ray spot to the required voxel size (or provide the user an indication of this), the actual and voxel resolution are usually the same, but this is not regularly tested or reported. It is possible to use resolution standards (such as

calibrated-thickness metal wires) to confirm spatial resolution and some reference standards exist, although a generally accepted standard for industrial CT systems does not exist. It is therefore possible that the amount of detail that is detectable in a scan can vary considerably from system to system, or even between different scans from the same type of system. The quality differences are either due to improper settings that possibly result in large X-ray spot sizes, or to improper choice of other scan parameters. The only way of testing the scan quality is to image a small feature of known dimensions and ensure the feature is visible in the CT slice image.

3.1.4 Scan time, number of images and rotational options

The major consideration for scan time is the acquisition time of single projection images, which can vary from system to system due to detector sensitivity and dynamic range differences, X-ray tube brightness differences, and differences in physical distance form source to detector [3]. A typical image acquisition time in a walk-in cabinet system with a 16-bit flat-panel detector is 500 ms per image, while some benchtop systems may have image acquisition times from a few hundred ms to up to several seconds per image. All systems have variable image acquisition times and therefore scan times can vary considerably. For highest possible quality scans, keep in mind to make full use of the dynamic range of the detector. In doing so, the image contrast is maximised by raising the image acquisition time up to near saturation of the detector for a particular X-ray setting. If the image acquisition time is too low, the resulting contrast will be poor with grainy images in extreme cases.

Some scanners involve continuous scanning (continuous rotation and image acquisition without steps), but the discussion here is limited to a stepwise rotation for simplicity. At each step position, one or more images can be acquired and averaged to provide an improved image quality (compared to a single image per position). This averaging reduces noise and therefore improves image quality, but its effect depends on the inherent noise of the detector used. For samples which may have small vibrational movements during rotational movement, e.g. leaves or hairs, it is advisable to use the skip function if available, which ignores the first image acquired at each new step position (during which time the sample stabilises). Since this vibration is due to the stepwise process, another approach is to use continuous scanning which also reduces vibration, but in that case averaging is not possible.

The number of step positions required depends on the sample size relative to the magnification. Therefore, the higher the magnification and hence the number of pixels used on the detector, the larger the number of images required for a good reconstruction.

A useful guideline in this regard (Guideline II) is:

i. The number of pixels covered by the sample on the detector in width (pixels) multiplied by 1.6 equals the number of projection step positions required. Consequently, up to a maximum of 3200 step positions are used for a typical 2000 pixel wide detector.

3.1.5 X-ray parameters: voltage, penetration values, background intensity and filters

X-ray projection images or radiographs can be viewed live and can be used for non-destructive analysis without the use of 3D reconstruction. In fact, this method of "digital radiography" is in wide use for industrial non-destructive testing purposes [20], though it is limited for complex objects and does not provide depth information, e.g. the presence of a void can be determined, but not how close it is to the surface or how close to other features in the line of sight. It is sometimes useful to use the live digital X-ray projection image as a fast scout method to quickly assess the inside of an object, thereby determining if a full CT scan is necessary (to provide a more detailed 3D view). An example of an X-ray projection image of the chameleon is given in Fig. 3 (b).

For CT scan setup, the radiograph or projection image is used as a basis for selecting good X-ray parameters, seen by the sample's penetration values and the background intensity values. Different types of samples require different X-ray voltages for best quality and this cannot always be predicted or estimated before a scan. The best possible material discrimination is obtained by using lower voltages. However, if a dense object is present, the X-ray penetration will be insufficient (sometimes unexpectedly) causing noise and artifacts. Beam hardening is the most common CT artifact, causing noise and artifacts (see section 3.1.6 for a detailed explanation). This penetration value is a percentage of detector counts around and through the sample.

To illustrate the negative effect of using too high voltage during scanning, an example of a biological museum specimen fitted with a metal name tag, can be used. Subsequently, very poor contrast will be obtained between the different materials and it would be advised to remove the metal name tag before scanning as the metal is much denser for X-ray to penetrate than that of the sample itself.

The choice of voltage is strongly related to the sample type and the goal of the scan, but some general guidelines (Guideline III) are as follows:

i. biological samples: 30 to 100 kV;
ii. small rocks and light metals: 60 to 150 kV;
iii. heavy metals (e.g. steel) and larger rocks: 160 to 240 kV or more; and
iv. in general: small samples require low voltage.

Commonly, there are two setups of filters, i.e. where the filter is placed between the source and the object, or between the object and the detector. The first setup, called beam filtering, is useful when the voltage is increased and a beam filter is added to pre-compensate for expected beam hardening. The filter effectively reduce the polychromaticity of the beam, thereby preventing streaky artifacts. Frequently used beam filters include 0.1 to 2 mm of copper and 0.5 to 1.5 mm tin or combinations of these, as well as aluminum, all used for beam filtration. Detector filtration, the second type of filter setup, can also be used to reduce noise if, due to the denseness of the object, secondary X-ray emission is produced. This may happen when a dense material strongly absorbs X-rays and re-emits lower energy X-rays by fluorescence, or when a large amount of scattering is present from nanostructured samples, causing X-ray scattering (deflection or diffusion of X-ray particles). In both cases, using a filter after the sample and before the detector shields the detector from low energy X-ray emission and scattering, limiting noise.

Guidelines IV are presented for the determination of adequate penetration values:

i. A typical setup method to find best settings for a particular sample type, is to rotate the sample until its 2D X-ray projection image shows the darkest region (its longest or densest axis) and then the user can calculate the sample's minimum penetration ratio compared to the background X-ray intensity (using the grey value counts measured in the X-ray image).
ii. If the penetration value is less than 10%, an increased voltage or current is required, whereas, if it is above 90% the voltage or current should be lowered.
iii. However, this might cause the detector to saturate, in which case beam filters can be applied to prevent saturation, while still increasing the penetration value.
iv. By using a beam filter, a higher voltage or current can be obtained with a reduction in the low energy X-rays such that the detector does not yet saturate.
v. Penetration values from 10 % to 90 % should result in good scan quality.
vi. In order to reduce penetration, lower voltages should be used.
vii. Since X-ray emission is typically very limited below 50 kV, longer scan times are then required to obtain large enough signals.

3.1.6 Scan quality problems and artifacts

As can be seen above from the many variations of scan settings, different quality scans can be obtained. Figs. 2 (a) to (c) shows micro-CT slice images of the chameleon with poor contrast, double edge and slight blur, respectively. Artifacts are also present in Figs. 2 (a) and (b). These are typical image quality problems that can occur. In particular, Fig. 2 (d) has poor contrast, in this case due to incorrect reconstruction setting (clamping) which will be discussed in Section 3.3. The same effect will occur when scanning a sample with the metal rotation table in the scan volume, or when too low voltage is used. Fig. 2 (e) has a double edge due to incorrect reconstruction setting (offset correction) which will also be discussed in Section 3.3. The same type of double edge will

occur to a lesser degree if the sample moves during a scan. Fig. 2 (f) shows only a slight blur on the edges, which is due to hardware misalignment, where the rotation axis of the sample and the X-ray tube direction is slightly more or less than 90 degrees (referred to as the tilt axis). This misalignment causes a reconstruction error since the reconstruction process assumes the rotation axis to be perpendicular to the beam direction. These images are meant to demonstrate typical image quality problems.

Insert Figure 2 approximately here

Beam hardening was already mentioned above when discussing choices of voltages, within the context of streak artifacts. However, for homogenous dense objects scanned with too low voltage, a “cupping” effect is also possible where the edge of the sample seems brighter than the inside. This artifact is present when insufficient penetration of the sample is obtained, for example due to too low voltage. Other artifacts and unwanted image effects include cone beam artifacts affecting the edges of materials near the edges of the detector, double edges due to tilt axis misalignment relative to beam axis, and blurring due to unstable rotational axis. The reader is also referred to relevant publications on CT artifacts by Barrett and Keat [21], as well as Boas and Fleischmann [22]. Additionally, Table 1 summarises problematic micro-CT scans as discussed in this paper, providing the cause(s) and a possible solution(s) to the problem.

Insert Table 1 approximately here

3.2 Scanning

Before a scan commences, the background must be normalised. This is done by removing the sample and using the X-ray beam at the chosen settings to correct for all intensity variations across the detector (e.g. the beam is more intense in the middle than the edges of the detector, but the normalised pixel intensity must be equal for a good scan). This normalisation or correction process can be done before every scan, but is only required if X-ray or acquisition settings change, or at the start of a day of scanning. It is also necessary to run a beam centering right before scanning to ensure the electrons are well focused for a optimal X-ray output. This is an automated process in most commercial systems.

Once the sample is loaded and settings chosen, a scan can be acquired. The scanning is done automatically with no user interaction, but errors can occur during the scan, requiring frequent supervision. For example, the X-ray source could become unstable and require a warmup, the filament could burn out and should be replaced.

Fast scans are sometimes required as long scan times have the potential to result in low image quality and artifacts and also to find optimal settings for particular sample types. Faster scans are achieved by using less images, no averaging and shorter exposure times. Such fast scans (e.g. 15 min) are not ideal, but can be sufficient for some purposes, such as identification of relatively large features, finding regions for further analysis or for higher quality scanning, even for simple measurements. Such fast scans can also be used as a scout method, to find a region of interest for a long, high quality scan.

3.3 Reconstruction

Reconstruction entails the creation of a 3D data set from 2D image projections and different options are available, as well as different data output types, as presented in this section.

3.3.1 Reconstruction options

After a full scan, the data is reconstructed, which refers to creating a discrete 3D data set from 2D image projections. Sometimes a reconstruction refers to a 3D image rendering or visualisation of a 3D data set, but we call this “visualisation” which will be describe this in the next section. The reconstruction process involves the mapping of each voxel, by using projection image representations of that particular voxel from many angles. This mapping is done by a Feldkamp filtered back-projection algorithm [23]. Commercial micro-CT systems all have built-in reconstruction software packages with slightly different available settings, but all based on the same algorithms. Volume Graphics (http://www.volumegraphics.com/) is a stand-alone software package mainly used for 3D image analysis, but also offers a module for reconstruction, and another commercial standalone software for reconstruction is offered by Inside Matters (https://insidematters.eu/), called Octopus Reconstruction.

Reconstruction software involves a series of choices or options, which can also affect the quality of the obtained 3D data. These options will be described in general here, though not all options are available in all reconstruction software packages. Firstly, the field of view can be cropped to make the total reconstructed volume smaller. This helps reducing the data set size and makes reconstruction faster since less memory is required, especially helpful when time or computing power is limited. Secondly, a choice of data type for the output can be made, which is usually selected as 16-bit, but 8-bit can be selected if storage space or memory is a problem. Then, the exact location of the rotation axis in each projection image is found by making use of an automated algorithm, which finds the central pixels in all 2D X-ray images – the use of the exact rotation axis in the back-projection algorithm improves the quality of the reconstruction and is especially important at higher resolutions. This process can also be coupled with a refinement process, correcting for small movement or shift of the sample or due to inaccuracy of the rotation stage. By viewing before-after projection images and correcting for any small shifts will seem

helpful. In advanced software packages, viewing can be done at more than one position during the scan, correcting for changes which occur at different times during the scan, although limited to very small changes. The result is, however, much improved edge clarity in the reconstructed data set. Another important consideration, is beam hardening correction, which corrects much of the generally-occurring “cupping” effect in samples where the edges seem brighter than the middle of the scan. By attempting (through trial and error) until the best value is found (values can be chosen in a range from light to heavy implementation of the algorithm), the correction can be achieved. Another option called clamping, involves the disregarding of a certain percentage of pixels that are “outliers” in terms of strong or weak absorption compared to the rest of the data, which effectively improves the grey value contrast in the images. Clamping can be very useful when a small quantity of bright dense phases are present, but are not of interest, and are therefore all grouped as the same brightest grey value. The % of pixels that are clamped, and the clamping direction (lowest or highest grey values only, or both) can be set. Furthermore, it may also be possible to make use of special settings to select the background detector counts in each image and normalise this across the series of images, which is useful when scattering is present, resulting in brighter or darker projection images from different angles. It is possible to use special algorithms to remove ring artifacts by disregarding “dead” pixels from the 2D projection images. Ring artifacts especially near the center of rotation are also removed by making use of a detector shift process, whereby the detector shifts horizontally between step positions and which are corrected in the reconstruction process resulting in a smoothing of the rotational center artifact. Lastly, for some scanners, it is also possible in region-of-interest (ROI) scans (where the sample extends over the sides of the 2D image), to remove the bright ring, which results around the outside of the scan volume, and hence improve the image quality by using a special algorithm dedicated for this purpose.

Therefore, it is clear that many different possibilities exist for reconstruction and hence this process is in itself an important step, which can assist in obtaining improved image quality even when using lower cost CT scan hardware. Since the reconstruction process itself can vary significantly, it is suggested to retain X-ray projection images even when reconstruction has been completed, thereby allowing future improved reconstruction of the same data, where possible.

It is also possible to use resolution reduction or oversampling in the reconstruction process, as well as filtering of the 2D projections using a variety of image filters. These are not standard and only used in specialised cases. Another option is to reconstruct scans of less than 360 degrees (usually 180˚), when a sample is too large to rotate fully in the system used, for example. During data collection, images may become corrupted or lost. A simple process of replacing this image with its adjacent image in the sequence, results in a good reconstruction, even though an image may be missing.

In medical CT, each voxel is typically associated with a calibrated Hounsfield Unit (HU) or CT number, which is regarded as the average attenuation in that section of the sample [24]. Some scanners allow calibration to HU by using calibrated specimens. However, in micro-CT the grey values are not calibrated and depend on reconstruction settings in the software used, as well as various scan parameters. In general, therefore the reconstructed data from micro-CT scans are not calibrated for density determination.

3.3.2 Data output types

Both the X-ray projection images captured by the CT system and the reconstructed CT dataset consist of pixels or voxels which have specific shades of grey. Each of the pixels or voxels are assigned a specific value which indicates the intensity of its shade of grey. These values differ depending on the bit depth assigned to it e.g. 8 bit = 2^8 = 256, which indicates the grey value range (in integral values) is from 0 to 255 and 16 bit = 2^{16} = 65536, which indicates that the grey value range is from 0 to 65,535. The advantage of the 16 bit dataset over the 8 bit dataset is that it has more capacity to differentiate between small density changes because of the larger grey value range providing a more accurate representation of the sample that was scanned. In general commercial micro-CT scanners have detectors with dynamic range of between 12 and 16 bit, so using 8 bit data sets result in loss of information. The advantage of 8 bit data types is the much smaller physical size (50% reduction) and hence easier handling for visualisation, analysis and transfer of data.

A general comment about image file types is warranted here. There are different image files types with the main difference being JPG and BMP and similar, which are Windows compressed image formats and TIFF which is uncompressed. The compressed file formats are lower quality and can results in loss of information. Though the use of a compressed file format can reduce data set size, it is advised to rather use poorer resolution scans to reduce data set size. In order to achieve this, during the setup step, the sample is moved back such that it fills less of the field of view of the detector which will result in a smaller data volume (and shorter scan time). Also worth mentioning is that 16-bit TIFF files cannot be viewed properly by windows image viewer programs. High quality image viewing programs such as Irfanview (http://www.irfanview.com/) and Adobe Photoshop (http://www.adobe.com/) are required.

Image stacks are calibrated in the sense that each isotropic voxel has a side length equal to the scan's voxel size and each slice image is spaced by the voxel size (evenly distributed). However, post-processing of this data and creation of images for viewing, including scale bars, do not necessarily maintain this pixel spacing, especially when creating lossy file types such as JPG. The original image stack must therefore be kept together with knowledge about the voxel size. In some

scanner types this information, together with all scan settings, are kept in a file which can be read in notepad (e.g. PCA file in General Electric Phoenix systems or TXT file in Nikon systems). Saving image stacks with a scale bar from image processing software is not suggested for two reasons: firstly, these scale bars are not accurate enough (rounded off) and secondly, having a scale bar in a slice image creates a long 3D scale bar, which is not useful.

3.4 Visualisation

Micro-CT data can be visualised in two different ways, i.e. volume rendering, as well as surface rendering. Volume rendering is typically done in a 3D data analysis software package and involves iso-surface views using a user-defined threshold value, or a user-defined greyscale gradient for more advanced 3D rendering algorithms. These differ from 3D Computed Aided Design (CAD) software in that they handle full voxel data, i.e. data exist everywhere in a 3D voxel grid, not only on surfaces of the object. In other words, CAD software packages use triangulated mesh data of surfaces only (point locations only), while full CT data comprises data at every point in 3D space (grey value at every point). Therefore, a volumetric data set is significantly larger and requires more intensive computing power, even for simple visualisation. Commonly used commercial software available for volume rendering include Avizo (https://www.fei.com/software/avizo3d/), Volume Graphics VGStudio (http://www.volumegraphics.com/en/products/vgstudio-max/), Amira (https://www.fei.com/software/amira-3d-for-life-sciences/) and Simpleware (https://www.simpleware.com/), whereas surface rendering software are Blender (https://www.reddit.com/r/blender/), SolidWorks (http://www.solidworks.com/) and Autodesk (http://www.autodesk.com/). Additionally, freeware (or open source) software, which can be used for analysis of CT data in 2D or 3D, include ImageJ (http://imagej.net/), MIPAR (http://www.mipar.us/), Blob3D (http://www.ctlab.geo.utexas.edu/software/blob3d/), Quant3D (http://www.ctlab.geo.utexas.edu/software/quant3d/), Blender (https://www.reddit.com/r/blender/) and 3dma_rock (http://www.ams.sunysb.edu/~lindquis/3dma/3dma_rock/3dma_rock.html). The reader is referred to the paper by Walter al. [25] for additional information regarding software options that allow visualisation of micro-CT data.

3.5 Image processing and analysis

The most widely used step in 3D image processing or segmentation is thresholding. This involves, in the simplest case, that a grey value is selected that will allow the viewing of all the brighter grey values in the respective volume when performing analysis. Also referred to as global thresholding, the same threshold will be used across the entire volume. Besides this simple thresholding step, individual features may be highlighted and analysed in detail with regards to its 3D location in the sample. Thresholding itself can be done in different ways, and the available methods depend on the software used and algorithms available. One valuable method is local adaptive thresholding, whereby a global threshold or region of interest is chosen as starting contour and local

thresholding is then applied. In addition to thresholding and region growing, a very useful method especially for biological sample segmentation is manual segmentation using a drawing tool, to add or remove regions with a virtual pen or brush. This method is a tedious process as it can involve selecting in regions in every slice in the CT data set, but is sometimes the best way to segment complex biological data sets. This process can be accelerated by making use of thresholding or other semi-automated segmentation tools and then refining these selections using the drawing tool (instead of making the entire selection only with the drawing tool). Another possibility is to strongly filter the data, which simplifies the automated segmentation tools, but can result in loss of quality for small features. This is useful when only viewing is required, and not complex dimensional or volumetric measurements. A basic typical image processing procedure is described below, but more details of further segmentation and image processing methods are described in Mathews & Du Plessis [26], including a step-by-step guide for the segmentation of frog bones taken from whole frog scans.

During image processing (prior segmentation), filters can be used to smooth the images and in doing so, to reduce random noise. This is especially useful for noisy data or fast scans, but sometimes this step can smooth over fine structural details, therefore its use is dependent on the image quality and aim of the analysis. Examples of such filters, are Gaussian or Median smoothing.

Segmentation usually follows the smoothing step, where some regions are selected using a variety of tools, and these selected voxels or regions are grouped together, creating individual regions-of-interest (ROIs) or image masks. This involves a binarisation step, where the selected voxels are given a value of 1 and the rest a value of 0 (or black and white).

The next step usually involves image analysis, which aims to extract both qualitative, as well as quantitative data from the images or image sequences [6]. The information from the range of 2D slices that are merged to create the 3D image allow for volumetric observations and measurements of the microstructure of the sample. Some tools available for micro-CT image analysis are: morphology analysis (used for bone structure i.e. mean trabecular number, trabecular spacing, volume fractions), defect detection (detection and size distribution analysis of pores / voids / cracks / inclusions), nominal / actual comparison (compare geometries of two voxel data sets based on their surface information), wall thickness analysis (measure thickness variations of walls of objects or layers of a material) and orientation analysis (orientation of fibers or longitudinal features in 3D) [3]. For more details of reconstruction and image processing post-scan, a recent article describes various options in more detail [27].

Analyses vary considerably depending on the application, especially in terms of time required. For some general industrial applications, specialised software modules exist to simplify the process of such analyses and minimise the human error involved in the process. Such routine analyses are therefore not very time consuming (usually less than 1 hour for each type of analysis, sometimes much less depending on complexity of data set). Biological data sets tend to be more complex and time consuming. Dimensional measurements can be performed in 2D slice views (linear distance measurements) or in 3D using volume or surface area measurements.

3.6 Further scanning at higher resolutions

In order to capture maximum detail it could be considered selecting only a specific (smaller) region of a sample, opposed to scanning the entire sample, consequently achieving a higher resolution (and more detail). The sample is thus sectioned and also referred to as a ROI scan of a section of the sample, which will be described in the 3.8.6.

3.7 Maintenance issues and usage schedules

Although some X-ray systems can be relatively maintenance-free (usually those with sealed X-ray sources), most commercial micro-CT systems require significant maintenance, which must be considered in terms of financial implications. Benchtop systems require less maintenance than cabinet and walk-in systems, with annual services and replacement parts contributing roughly 10% (depending on the usage) to the purchase price of the system. Additionally, typical usage for a large cabinet based system requires approximately 10 to 20% down-time for repairs and maintenance, as well as consumable replacements. It should be noted that filament replacement is required approximately once a week.

Another consideration regarding micro-CT scanning is the frequency of hardware failures. Such failures are common when performing continuous-rotation scans. Consequently, failure of any kind (even of only one image) causes the entire scan to fail and therefore using stepwise rotation is advised. For these reasons, the availability of local technical support from a supplier is a major consideration, along with the system type. In general, the larger the system the more maintenance is required.

In a multi-user facility the systems are operational for long periods, and there is a need to streamline and automate the workflow. For this purpose, some suppliers offer automated sample loading (e.g. 12 samples pre-loaded in a rotating sample mount), automated choice of scan settings and even sending of emails or phone messages when a scan is completed (or failed). Automated batch reconstruction, as well as batch image processing and analysis is possible on multiple scan data sets done overnight, when using macros written by the user. A limitation is, however, that the procedure should be very simple as there is nobody to check the result. None

the less, the most widely used method of getting optimal samples done in the available time span, is to load samples vertically and then scan each one sequentially. In this way, approximately 3-10 samples can be scanned overnight, depending on the scan parameters and assuming no hardware failures occurs during the scan time.

3.8 Micro-CT scanning of a three-horned chameleon – an example of data acquisition and analysis

The considerations, guidelines and options related to micro-CT scanning of biological samples are presented here and could be used as guiding principles when conducting micro-CT scans and analysis. The three-horned chameleon is used as an example and will follow the step-wise guidelines as presented in this paper.

3.8.1. CT set-up parameters:

i. The chameleon (wet with alcohol from the collection jar) was mounted on florist foam on top of a cardboard tube (Fig. 3 (a)), after being dried out at ambient conditions for a few hours in this position. Caution should, however, be taken that the sample is fit for drying as some samples will change morphologically during drying. The chameleon is seen with its densest and thickest features as darker regions by looking at a digital X-ray projection image of the specimen (Fig. 3 (b)).
ii. The chameleon's size was adequate for scanning in a cabinet system (total field of view approximately 100 mm wide x 100 mm high), but would also have been possible in a benchtop instrument.
iii. This example had 2000 pixels in total sample height, and using Guideline I, the best possible resolution was obtained at 75 µm. Following Guideline II, since the best possible magnification was at 75 µm voxel size, 3200 step positions were used. Since the sample was loaded at 45 degrees, there was a slight improvement in the best possible voxel size compared to horizontal or vertical mounting, for a single scan volume (vertical or horizontal would be limited to the longest axis of the chameleon sample). A further improvement could be obtained by loading the sample vertically and allowing it to rotate within the edges of the detector, but this requires multiple scans. Therefore, a single scan optimal resolution is found for an elongated sample at 45 degrees.
iv. Averaging was set to 2 and skipping of the first image at each new position was used.
v. The combination of instrument, target, energy and characteristics of the sample did not require beam filtration.
vi. Initially, a typical image acquisition time of 500 ms was set, with 100 kV and 100 µA as starting point, with no beam filtration. This setting showed a good penetration value, but not very high signal values on the detector, so the current was increased to 200 µA to obtain approximately 8000 counts, where 10 000 is the saturation level of the detector (Guidelines III & IV). In this process a compromise between scan time and image quality was found.

Higher quality would have been possible with more averaging, resulting in longer scan times. Higher quality would also have been possible at lower voltage since the penetration values were quite high. When lowering the voltage, the total X-ray emission from the source reduces, which requires a longer image acquisition time to make full use of the best possible contrast capable with the detector. This also increases scan time and additionally, lower voltages can cause unexpected artifacts as explained above.

Insert Figure 3 approximately here

3.8.2. Scanning: Corrected the background by taking the sample out and creating a smooth background image, ran a beam centering and loaded the sample that was mounted on florist foam and started the image acquisition process. The process was monitored to correct for any errors.

3.8.3. Reconstruction settings used for the chameleon scan included: cropping to remove unwanted regions around the edges using the manual crop editor, selecting the 16 bit data type, correcting for offset by using a scan optimisation process. Additionally, a a low beam hardening correction value and a background intensity value was used to correct for variations in intensity.

3.8.4. The 3D visualisation of the chameleon is shown in Figs. 4 (a) and (b), using a simple thresholding function allowing the visualisation of the skeleton structure which is denser than the rest of the animal. The data set was processed to virtually remove the mounting material and smoothed to produce a clean surface rendering using adaptive Gauss default image filtering. Image processing steps are described in more detail in the next section.

3.8.5. Image processing and analysis:

As one example, an interesting bony feature is highlighted in red in Fig. 4 (c). This structure is a part of the very strong tongue of the animal. A slice image shows more detail and is shown here in the plane of the bony feature, indicating that this bony feature is extremely straight. It is possible to view this in 3D rendering as well by making the rest of the animal semi-transparent and the feature of interest non-transparent. An example of image analysis would be to measure the length and width of the bony feature of the tongue of the chameleon, as well as determine its surface area and volume.

Insert Figure 4 approximately here

3.8.6. Further scanning at higher resolutions:

Without the need for physical sectioning, the head of the animal was scanned at a higher resolution of 30 µm (with a field of view of about 30 mm) compared to the full body scan of 75 µm

(field of view 75 mm). The higher resolution allows smaller features (i.e. skeleton structures (Fig. (5)) to be visualised. To maintain a high resolution of 30 µm and scan the entire sample, an automated multiple-scan process could have been used. This entails a sequence of scans that are performed at different height positions across a vertically mounted sample. The multiple scans are then stitched together to form a large data set and it should be noted that it is a lengthy process.

Insert Figure 5 approximately here

To obtain sub-micron resolution of the horn of the chameleon, physical sectioning thereof was needed. Images depicting the improvement in resolution (from 10 µm to 0.95 µm) are depicted in Figs. 6 (a) to (e). The 10 µm scan was done in a nano-CT instrument rather than the micro-CT as better image quality was expected, although most micro-CT models can also scan at a 10 µm resolution.

Insert Figure 6 approximately here

4. Summary

3D laboratory micro-CT is a fast growing non-destructive testing and analysis method in scientific research applications. The increasing accessibility of such instruments will lead to an increasing number of new discoveries in scientific research applications. The aim of this paper was to provide a focused "how-to guide" for new potential users to better plan their work, and understand how to best make use of this technology. A specific new case study was used as demonstration – the Jackson's three-horned chameleon specimen, which was scanned at different settings, and the full data sets provided as supplementary information. These data sets are meant to be used to gain a better understanding for viewing and handling typical 3D data sets from the technique. Scans up to sub-micron resolution resolved osteocyte structure in the horn of the chameleon. These observations demonstrate a typical multiscale investigation by X-ray micro- and nano-CT.

5. Acknowledgements

The Botany and Zoology Department at Stellenbosch University is thanked for lending the chameleon specimen used in this study.

6. References

1. Kalender WA. X-ray computed tomography. Physics in Medicine and Biology. 2006;51(13):29-43

2. Brunke O. High-resolution CT-based defect analysis and dimensional measurement. Insight-Non-Destructive Testing and Condition Monitoring. 2010;52(2):91-3

3. Singhal A, Grande JC, Zhou Z. Micro/Nano CT for visualization of internal structures. Microscopy Today. 2013;21(02):16-22

4. Mizutani R, Suzuki Y. X-ray microtomography in biology. Micron. 2012;43(2):104-15

5. Betz O, Wegst U, Weide D, Heethoff M, Helfen L, LEE WK. Imaging applications of synchrotron X-ray phase-contrast microtomography in biological morphology and biomaterials science. I. General aspects of the technique and its advantages in the analysis of millimetre-sized arthropod structure. Journal of Microscopy. 2007;227(1):51-71

6. Schoeman L, Williams P, du Plessis A, Manley M. X-ray micro-computed tomography (μCT) for non-destructive characterisation of food microstructure. Trends in Food Science and Technology. 2016;47:10-24

7. Cnudde V, Boone MN. High-resolution X-ray computed tomography in geosciences: A review of the current technology and applications. Earth-Science Reviews. 2013;123:1-17

8. Maire E, Withers PJ. Quantitative X-ray tomography. International Materials Reviews. 2014;59(1):1-43

9. Landis EN, Keane DT. X-ray microtomography. Materials Characterization. 2010;61(12):1305-16

10. Faulwetter S, Vasileiadou A, Kouratoras M, Dailianis T, Arvanitidis C. Micro-computed tomography: Introducing new dimensions to taxonomy. ZooKeys. 2013;263:1

11. Lenihan J, Kvist S, Fernández R, Giribet G, Ziegler A. A dataset comprising four micro-computed tomography scans of freshly fixed and museum earthworm specimens. GigaScience. 2014;3(1):1

12. Landschoff J, Plessis A, Griffiths CL. A dataset describing brooding in three species of South African brittle stars, comprising seven high-resolution, micro X-ray computed tomography scans. GigaScience. 2015;4(1):1

13. Stauber M, Müller R. Micro-computed tomography: a method for the non-destructive evaluation of the three-dimensional structure of biological specimens. Osteoporosis: Methods and Protocols. 2008:273-92

14. Metscher BD. MicroCT for developmental biology: A versatile tool for high-contrast 3D imaging at histological resolutions. Developmental Dynamics. 2009;238(3):632-40

15. Pauwels E, Van Loo D, Cornillie P, Brabant L, Van Hoorebeke L. An exploratory study of contrast agents for soft tissue visualization by means of high resolution X-ray computed tomography imaging. Journal of Microscopy. 2013;250(1):21-31

16. Kotwaliwale N, Singh K, Kalne A, Jha SN, Seth N, Kar A. X-ray imaging methods for internal quality evaluation of agricultural produce. Journal of Food Science and Technology. 2011;51(1):1-15

17. Duliu OG. Computer axial tomography in geosciences: an overview. Earth-Science Reviews. 1999;48(4):265-81

18. Kak AC, Slaney M. Principles of computerized tomographic imaging New York: IEEE Press; 1988.
19. Lin C, Miller J. Cone beam X-ray microtomography-a new facility for three-dimensional analysis of multiphase materials. Minerals and Metallurgical Processing. 2002;19(2):65-71
20. Hanke R, Fuchs T, Uhlmann N. X-ray based methods for non-destructive testing and material characterization. Nuclear Instruments and Methods in Physics Research Section A: Accelerators, Spectrometers, Detectors and Associated Equipment. 2008;591(1):14-8
21. Barrett JF, Keat N. Artifacts in CT: recognition and avoidance 1. Radiographics. 2004;24(6):1679-91
22. Boas FE, Fleischmann D. CT artifacts: causes and reduction techniques. Imaging in Medicine. 2012;4(2):229-40
23. Feldkamp L, Davis L, Kress J. Practical cone-beam algorithm. Journal of the Optical Society of America. 1984;1(6):612-9
24. Donis-González IR, Guyer DE, Pease A, Barthel F. Internal characterisation of fresh agricultural products using traditional and ultrafast electron beam X-ray computed tomography imaging. Biosystems Engineering. 2014;117:104-13
25. Walter T, Shattuck DW, Baldock R, Bastin ME, Carpenter AE, Duce S et al. Visualization of image data from cells to organisms. Nature Methods. 2010;7:S26-S41
26. Matthews T, Du Plessis A. Using X-ray computed tomography analysis tools to compare the skeletal element morphology of fossil and modern frog (Anura) species. Palaeontologia Electronica 2016;19(1): 1-46
27. Liu Y, Kiss AM, Larsson DH, Yang F, Pianetta P. To get the most out of high resolution X-ray tomography: A review of the post-reconstruction analysis. Spectrochimica Acta Part B: Atomic Spectroscopy. 2016;(117):29-41

Figure captions:

Figure 1. The fundamental components of a micro-CT instrument.

Figure 2. Micro-CT slice images of the chameleon, where (a) a metal tag is included in the scan volume, resulting in streaky artifacts, (b) too low voltage was used and therefore image artifacts are found around dense parts of chameleon, (c) too high voltage was used, showing poor contrast, (d) poor images are caused by reconstruction clamping set too high, (e) double edges are present due to incorrect offset calculations during reconstruction, and (f) slight blur due to tilt-axis misalignment. The arrows point to artifacts.

Figure 3. Images demonstrating the mounting of the three-horned chameleon with (a) showing the florist foam mounting material that forms the basis on which the specimen is placed, and (b) the X-ray projection image showing the very low density of the mounting material.

Figure 4. 3D reconstructions of the three-horned chameleon with a (a) surface view, (b) semi-transparent view showing the dense skeleton structure in yellow and (c) an isolated bony feature related to the tongue of the animal is shown in red in a 3D view.

Figure 5. A high resolution ROI scan (30 µm) of the head of the three-horned chameleon with showing the skeleton view.

Figure 6. Nano-CT slice images of (a) the horn of the chameleon at 10 µm, (b) the dense structure of the bone inside the chameleon horn (4 µm), with (c) visualising the bone structure in 3D, (d) revealing osteocyte distribution in the tip of the chameleon horn's internal bony microstructure at sub-micron resolution of 0.95 µm, with (e) showing the osteocytes in 3D along with a colour-coded volume analysis (termed defect analysis though these are in fact osteoctytes).

Tables:

Table 1. Summary of faulty or problematic CT scans as discussed throughout this paper, stating problems, causes and possible solutions, respectively.

Problem	Cause	Solution
Grainy image	Image acquisition time too low	Increase image acquisition time
Streaky artifacts	Differences in absorption from different angles; X-ray penetration is insufficient	Increase voltage
Poor contrast	Too high voltage is used	Reduce voltage
Blurred image	Improper sample mounting; allowing sample to move during scanning	Proper mounting to ensure no movement during scanning
Stitching artifacts / vertical or horizontal line	Reconstruction algorithms when stitching sample that is too wide for a single scan	Make sub-sections of sample; use a smaller sample or less magnification
Beam hardening / cupping effect	Insufficient penetration of the sample	Reconstruction: use beam hardening correction option, or scan with higher voltage and more beam filters
Small movement or shift (double edge)	Inaccuracy of rotation stage or movement of sample	Reconstruction: do an offset correction; or rescan if offset cannot be corrected. Reset stages. Hardware could be faulty, e.g. tilt axis alignment
The image is very dark on materials of interest, with bright spots in places	Small quantity of bright dense phase are present, but irrelevant	Reconstruction: make use of the clamping option
Scattering	Causes brighter or darker projection images from different angles	Reconstruction: select background detector counts in each image and normalise across the series of images
Ring artifacts	Bright rings are visible in the top slice view	Reconstruction: make use of ring artifact reduction by disregarding 'dead' pixels from the projection image (or disregard pixels in the

		acquisition process)
Central rotation artifact	The center of rotation is visible as a line in a side slice view, or a dot with concentric rings from the top view.	Make use of detector shift option in acquisition, which smooths out the artifact.
Bright ring around outside of scan volume, resulting in poor image quality	In ROI scans where the sample extends over the side of the 2D image	Use special reconstruction algorithm which corrects for this, or crop the ROI further in reconstruction
Cone beam artifacts	Affecting the edges of materials near the edges of the detector	Use less magnification to fill less pixels on detector

 Click here to download Figure Fig 1.tif

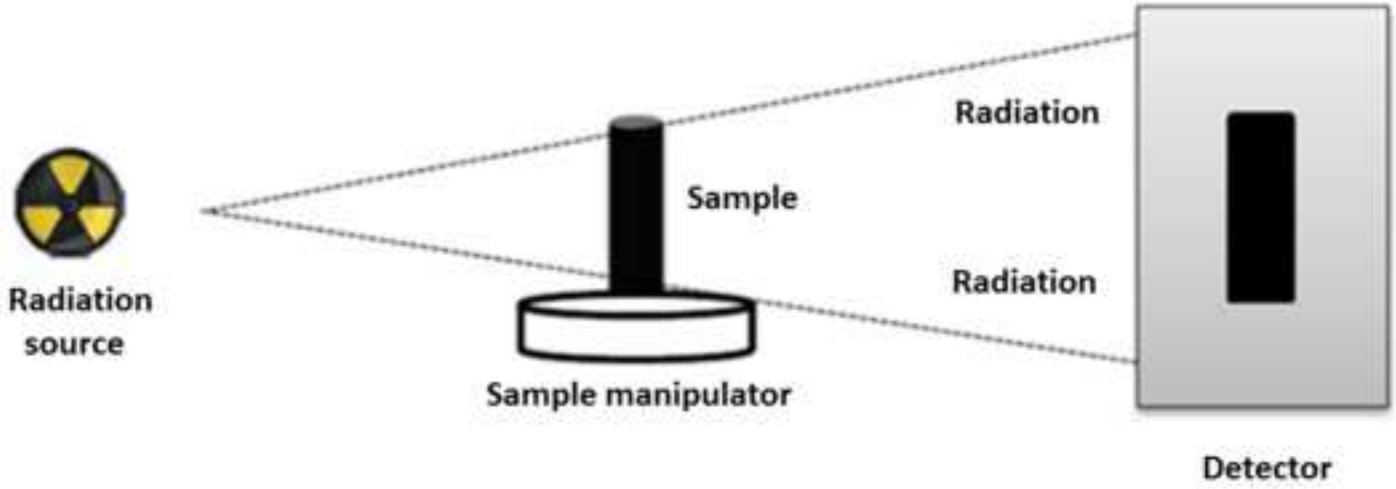

Figure

Click here to download Figure Fig 2.tif

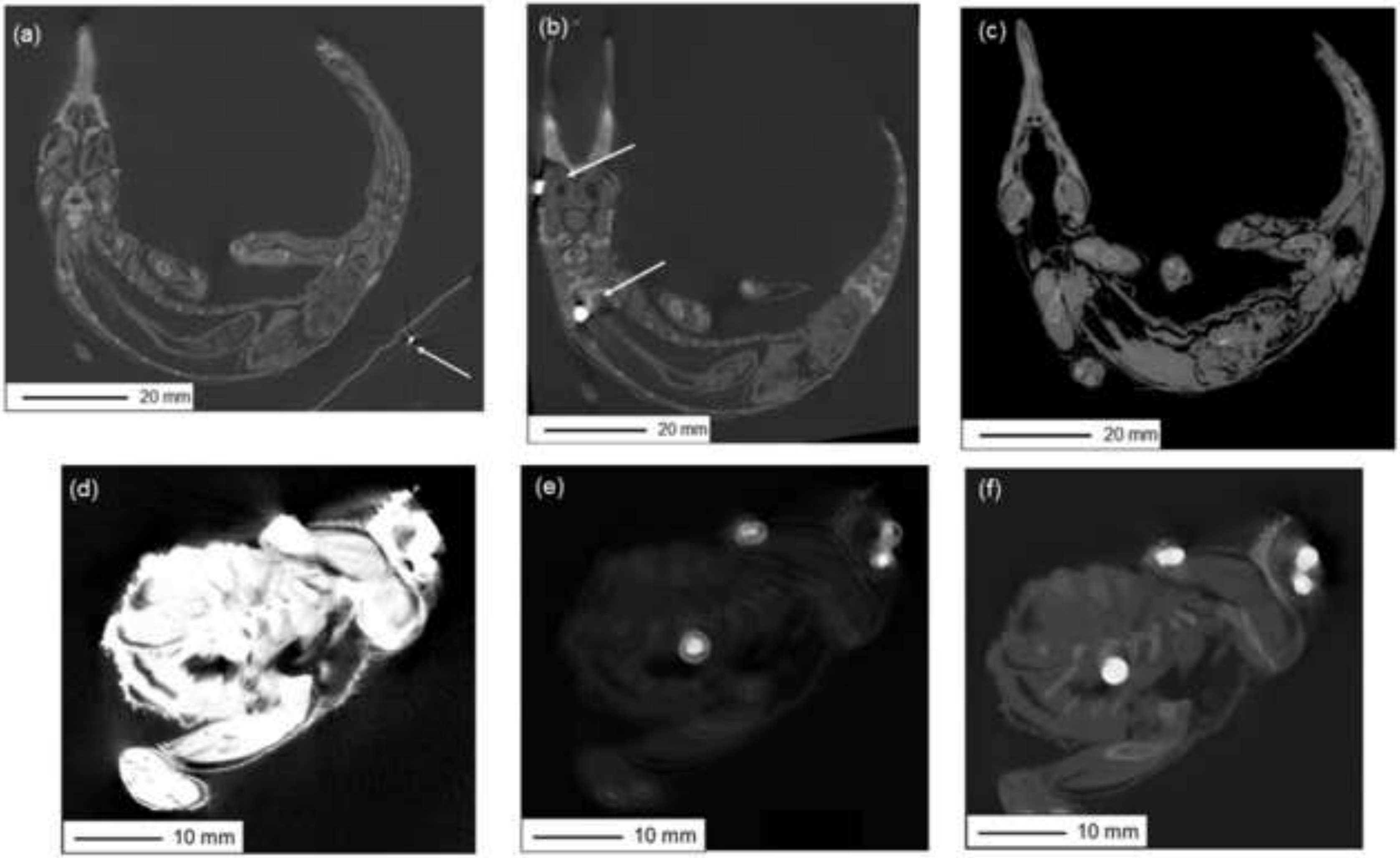

 Click here to download Figure Fig 3.tif

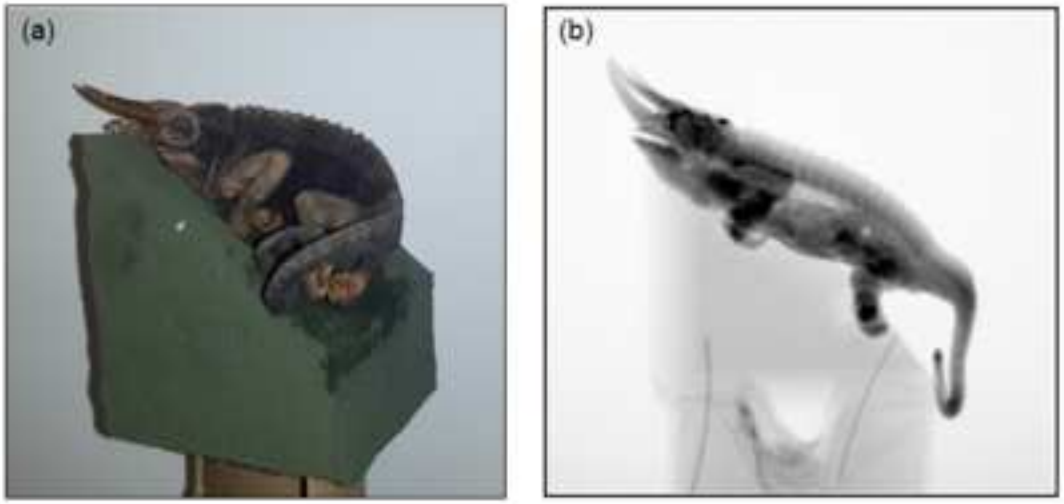

 Click here to download Figure Fig 4.tif

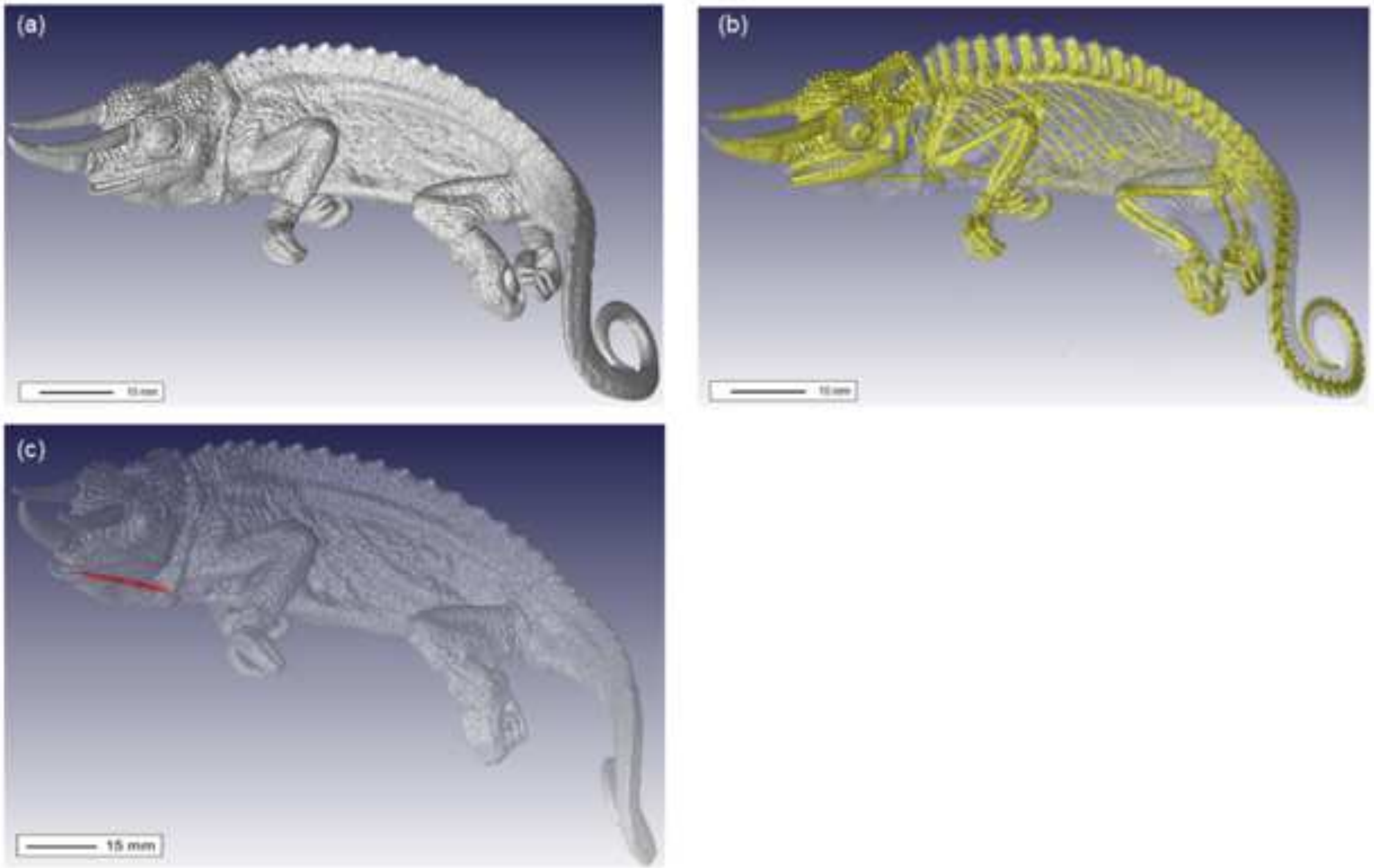

Figure Click here to download Figure Fig 5.tif

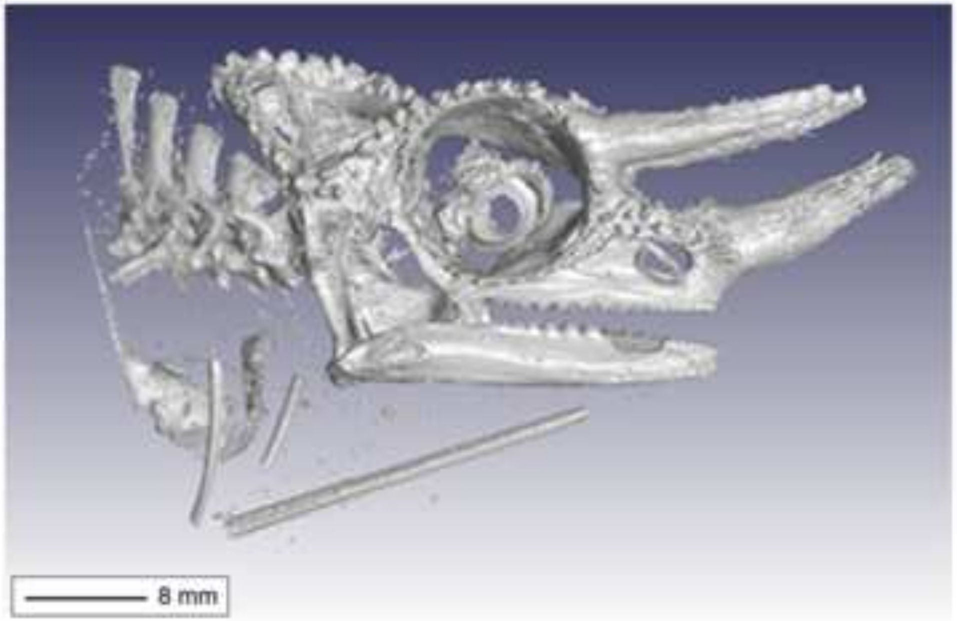

Figure

Click here to download Figure Fig 6.tif

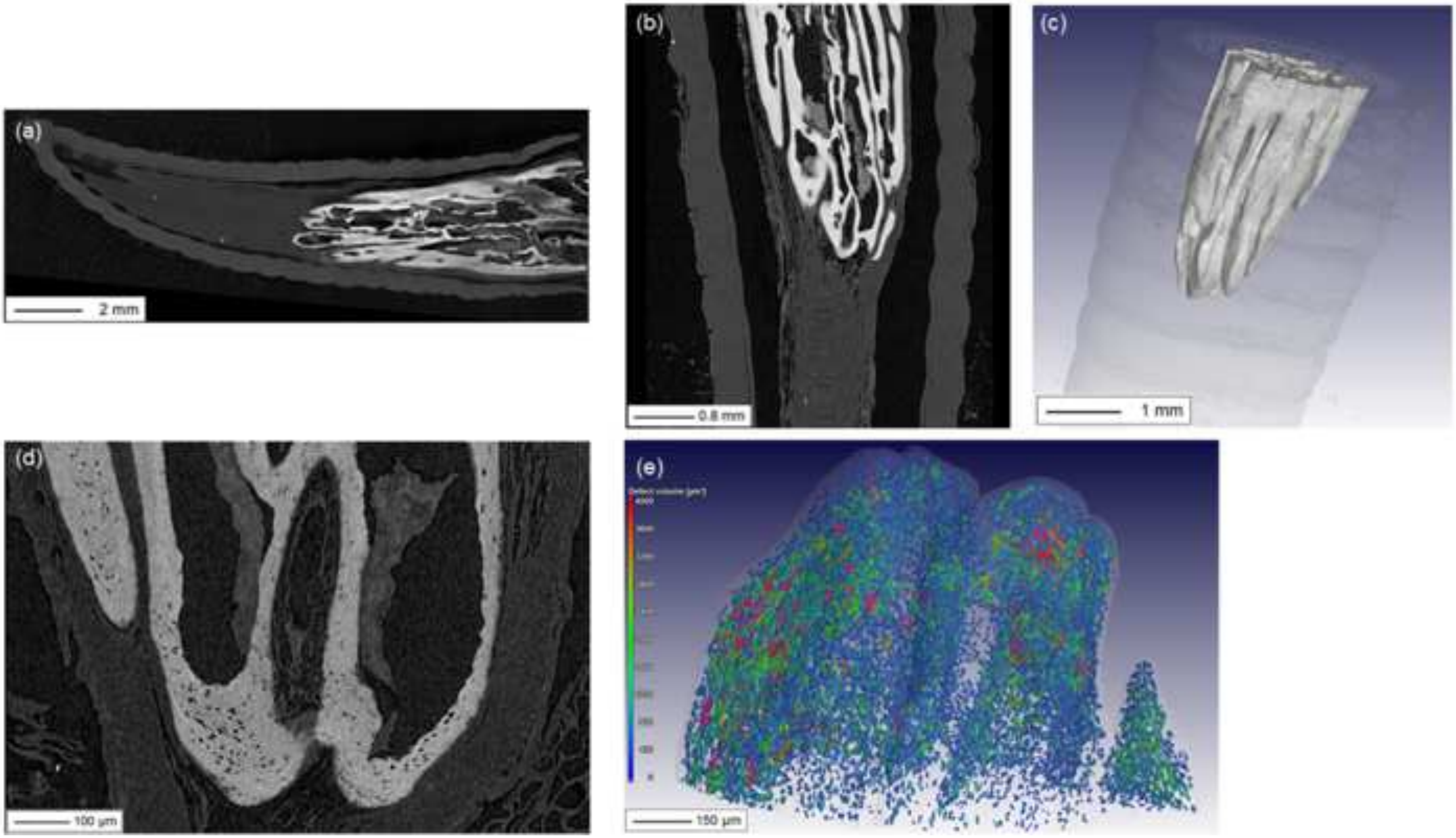

Click here to download Personal Cover Cover Letter_GigaScience REBUTTAL 22 July.doc

22 July 2016

Dr Scott Edmunds
Editor
GigaScience

Dear Dr Edmunds

Re: Resubmission of reviewed manuscript (GIGA-D-16-00031) for GigaScience

We hereby re-submit our reviewed manuscript titled: **Laboratory X-ray micro-computed tomography: a user guideline for biological samples** (previous title: Laboratory X-ray micro-computed tomography: a generalised approach for biological samples using a three-horned chameleon as example). In this new submission the points raised by the reviewers have been addressed and, once again, the reviewers are thanked for their thorough and valuable contributions towards improving this manuscript.

The changes made to this manuscript are described point-by-point (in red) in response to reviewer comments below.

Reviewer #1:

General comments:

- You have chosen an inappropriate way to conduct this type of analysis using a large zoological wet sample. Every curator that knows what she or he is doing would not permit this kind of semi-destructive analysis using an ethanol-preserved specimen. In your particular case, the animal should either have been scanned in its original jar, in a closed plastic jar filled with a little ethanol or would have best been transferred to a plastic container completely filled with ethanol. Obviously, you then end up with the problem of even less tissue contrast and might - depending on what kind of tissue you need to visualize - have to stain the specimen, something that might be problematic using type material in particular. The approach you have chosen is OK if you just want to see mineralized parts (e.g. a vertebrate's skeleton), but in many, many zoological samples there are either no mineralized parts or they are of little interest. Your analysis therefore only applies to a very small range of potential zoological samples (mostly vertebrates), thus rendering your guideline inappropriate for the remaining zoological species. Try to modify your text according to this issue.

Under section 3.1.1 the following sentences were added:

"However, some soft-tissue samples are preserved in a liquid and will damage if removed, therefore requiring scanning in the liquid as is."

and

"It should, however, be noted that in particular cases samples need to remain in their ethanol filled jars or be transferred to plastic container completely filled with ethanol when being scanned. In such cases, too much damage will be inflicted on the samples during manual handling."

- The article is too long for being a "how-to guide" - I therefore believe that the manuscript will profit significantly from a reduction of the text to about 60% of its current length. There is no specific paragraph or section that should be removed, but rather the authors should try to condense the text by removing overly generalized sentences and redundant information

An effort was made in this respect as redundant information was removed and paragraphs were condensed. Many figures were also removed, shortening the manuscript.

- The article is at current a mix between a "Protocols" type of article (bullet points) and a "Methods" paper (running text). I would suggest to restructure most sections into running text were bullet points are currently used (e.g. 3.3.1. i. - ix. or 3.8.1. i.-vi., and especially 3.8.2.-3.8.4.). Usage of bullet points should be restricted to the five guidelines

Sections 3.3.1, as well as 3.8.2 – 3.8.4 were changed into running text, but 3.8.1 was left as bullet points. The authors reason that the bullet points are necessary as it structures the text with respect to the guidelines that has to be linked with the chameleon example.

- Given the complexity of the structure of the text (number of headings and subheadings), please consider adding a "Content" section - if the journal style permits - at the beginning of your article

Unfortunately the journal style does not permit a Content section.

- Language/grammar: please check your article carefully for spelling and grammar mistakes - I am not a native English speaker, but I spotted a number of mistakes throughout the text, figure legends, and tables

The language and grammar of the manuscript was thoroughly checked and corrected where applicable.

- Figures I: there are too many: remove 3 (not very informative), fuse 4 & 5, remove 6 (redundant with text/confusing), fuse 8 & 9, fuse 11 & 12 & 13

Figs. 2, 3 & 6 were removed, whereas Figs. 4 & 5 were fused, as well as 8 & 9 and also 11 - 13.

- Figures II: with your article you are targeting a readership that is accustomed to concise yet appealing figures. At present, most of your figures do not adhere to the basic standards in zoological articles and should therefore be amended: 1. Create proper figure plates with minimum spacing between individual images, 2. Use one type of standardized scale in all figures, 3. Label structures - what are we looking at? Consult with a vertebrate zoologist, 4. Crop images and remove all uninformative space (e.g. in Fig. 4 roughly 50% of each image are not needed), 5. Place image designators (e.g. (a), (b)) in the upper left corner of each image, 6. Restructure figure plates in such a manner that the final figure is broader than long (e.g. Fig. 10 should be composed of four images arranged in a rectangle rather than three images in a row)

All these comments were addressed in the new figures.

- Please remove the line numbers in your original document file, the submission system is adding new numbers on top of your own line numbers

Line numbers were removed

- I would recommend that, as a test prior to resubmission, you ask a zoologist that has never been in contact with a µCT scanner to read your manuscript to see if he or she can grasp what you want to communicate. I have the impression that the terminology in some parts might be too specific for a newcomer

A zoologist read the manuscript and relevant comments were addressed.

Specific comments

- Keywords: some are redundant with the title - please remove

“Industrial CT” and “three-horned chameleon” were deleted; “testing” was replaced with “analysis”

- Title is too long: please remove "using a three-horned chameleon as example". Your intention is to provide a general guideline, the example is irrelevant in this context

The title was shortened to: “Laboratory X-ray micro-computed tomography: a user guideline for biological samples”

- Your line 18: "fascinating" - such terminology should be removed from the entire article. This is your personal opinion

“fascinating” and other likewise words were removed from the manuscript

- Your line 30: remove "medical", the abbreviation CT stands for "computed tomography", not "medical computed tomography"

“medical” was removed

- Your line 45: add "µCT"

"µCT" was added

- Your line 60: add reference for SRµCT, e.g. Betz et al. 2007 J Microscopy 227:51-71

Betz et al. 2007 was added as a reference for SRµCT

- Your line 96: should read "usually 180° or 360°", I know of many colleagues that in order to reduce scan time no longer are scanning at 360°

“180° or” was added to the sentence

- Your line 110: remove abbreviation "(CT)" from title

“CT” was removed

- Your line 122: remove URL

The URL was removed

- Your line 127: guidelines - please think of another way to distinguish the five guidelines from the remainder of the text. Italics look inappropriate to me, use regular font

Regular font instead of italics was used for the five guidelines

- Your line 136: the statements made here on staining specimens are too generalized, I recommend consulting literature currently not cited in your paper, e.g. Metscher 2009 BMC Physiology, Gignac et al. 2016 J Anat doi: 10.1111/joa.12449

In the last paragraph of the Introduction two papers on staining was additionally cited. Section 3.1.1 has been changed accordingly and now reads: “However, some soft-tissue samples are preserved in a liquid and will damage if removed, therefore requiring scanning in the liquid as is. In these cases, staining increases the contrast of the specimen compared to the surrounding medium [4, 12, 11].”

- Your line 163: "cloth + skin" - this is indeed a very bad approach, please see my general comments. In order to circumvent the issues you are mentioning I have previously placed objects in ethanol-filled plastic jars and then have used very thin plastic balloons (the squeezing end of a plastic pipette) to position the animal at the center of the jar. Very tedious process, but it works. Although it usually means that the researcher could be experimenting around for an hour or more in order to find the best way to position his/her specimen in the container and then in the µCT scanner

This has been addressed in the general comments.

- Your line 205 + 206: just use one scale for all values here: µm

Changes were made in order to use only one scale (µm)

- Your line 441: because of the points mentioned here, please also provide the raw data (i.e., the projections) of each scan together with the image stacks. This aspect has been discussed in Lenihan et al. 2014 GigaScience 3:6

The projections will also be provided along with the image stacks

- Your line 468: should read "0 to 255"

The line was changed and now reads: "0 to 255"

- Your line 469: should read "0 to 65,535"

The line was changed and now reads: "0 to 65,535"

- Your lines 504-508: this selection appears random and does not specify whether the software is free or commercial. Please look for articles that provide such information, e.g. Walter et al. 2010 Nat Meth Suppl 7:S26-S41 or better more recent papers

Section 3.4 was adjusted to incorporate the different free or commercial sofwares and the last paragraph now reads: “Commonly used commercial software available for volume rendering include Avizo, Volume Graphics VGStudio, ImageJ, Blob3D, Amira and Simpleware, whereas surface rendering software are AutoCAD, Blender, SolidWorks and Autodesk. Additionally, freeware (or open source) software which can be used for analysis of CT data in 2D or 3D include

ImageJ, MIPAR, Blob3D, Quant3D, Blender and 3dma_rock. The reader is referred to the paper by Walter et al. [22] for additional information regarding software options that allow visualisation of micro-CT data."

- Your line 604: there are also systems on the market that are almost maintenance-free, in particular those with a sealed X-ray source

To incorporate this comment, Section 3.7 first paragraph was changed and now reads: "Although some X-ray systems can be relatively maintenance-free (usually those with sealed X-ray sources), most commercial X-ray micro-CT systems require significant maintenance, which must be considered in terms of financial implications."

- Your line 696: remove "(due to its use for catching prey)"

"(due to its use for catching prey)" was removed.

- Your line 717: why would it be "more costly" if the scans are for free?

This sentence has been changed and now reads: " The multiple scans are then stitched together to form a large data set and it should be noted that it is a lengthy process". No more mention is made of the price.

- Your line 735: remove the entire sentence starting with "It is similar to most..." - you are addressing newcomers to µCT, so how can they "well know" benchtop µCT systems?

This sentence has been removed.

- Your line 759: remove sentence starting with "Data sets are included..." - redundant content and inappropriate terminology

This sentence has been removed.

- Your line 772: this is text that should be written by a zoologist - either modify and correct it or remove it

This text has been removed.

- Your line 755: remove second half of sentence - speculative and exaggerated

This text has been removed.

- Table 1: it would be nice to have visual examples for all artifacts/issues listed here - see, e.g. Fernandez et al. 2014 PLoS ONE 9:96617

Unfortunately, the authors were not able to provide visual examples of all the artifacts listed in Table 1, therefore the table was left without adding any figures.

- Deposited data: please rename your individual stacks in a consistent manner, e.g. Full body stack 75 µm, Head stack 30 µm etc. Add the specimen ID for future reference

The deposited data was renamed according to the reviewer's suggested names and will be uploaded for the final version of the manuscript.

The specimen ID is in the manuscript and since there is only one specimen, this should be clear enough.

- Figures: please see my comments in the general comments section above

- Figure 1: improve by adding more labeling, e.g. sample, radiation (as opposed to attenuated radiation) and by centering/improving the labels in the figure

These comments were all addressed in the new Figure 1

- Figure 2: (b) is shown from an oblique angle - inappropriate to illustrate what you want to convey; a + b vs. c are extremely different images - the context should be made clearer by showing step-wise 1. mounting of the animal and then 2. positioning it in the scanner

Figure 2 was rather removed.

- Figure 3: remove

Figure 3 was removed.

- Figure 4: these images should be taken at the same level for direct comparison

These specific levels show / illustrate the artifacts best, and was therefore not changed.

- Figure 6: remove

Figure 6 was removed.
- Figure 9: remove the red cross marks from the image - artificial structure
This specific figure was removed.
- Figure 10: (a) why is the head surface-rendered in green when it is grey in Fig. 8? Check all figures for consistency (same applies to skeletal parts...yellow, grey). Remove the cropping box from the image (c) - you might then need to choose another angle to illustrate what you want to show
Figure 10 (now Figure 5) has been reduced to only one figure, removing (a) and (c) and only showing (b), the skeleton view.
- Figure 11: remove "35%" - what does this mean?
"35%" was removed.
- Figure 12: orient images in same way - why is (b) shown oblique?
The orientation of the images were kept as they were. Fig. 6 (c) is presented tilted to illustrate the internal structures at best.
- Figure 13: as in Fig. 12 - why are they oriented differently? Also, remove the labeling "Defect volume" - an osteocyte is not a defect
The different orientations were chosen to view the detail as best as possible.
The caption for Fig. 12 (now Fig. 6) makes no reference to “defect volume” any more.

Reviewer #2

General comments:
- In parts, the language and style of the ms is rather narrative and could be more parsimonous and concise, containing many empty phrases such as "it is important to note", "generally", "more generally", etc. which can often be omitted. In addition, phrases such as "being a fascinating test sample", "the mystery that surrounds the scanning process" are, in my opinion, not an appropriate scientific writing style and sound rather strange. I may refer to text sections that could be improved in style in my more specific comments below, but as I won't be able to mention them all, the paper may benefit from an overall linguistic editing to improve the style. Several small grammatical and typographical errors will also need to be seen to.
The manuscript was revised with the aim of improving the writing style, as well as to correct the grammatical and spelling mistakes.
- A number of important publications in the field of biological micro-CT imaging are not mentioned in the ms and should be included, as they provide very valuable further reading for users new to micro-CT. Many of the points explained by the authors have been described in slightly different ways (or with additional information) by other authors, and this publication would benefit enormously from a comprehensive literature overview as it would then serve as a good starting point for new micro-CT users. A few missing literature references will be listed below in the specific comment section, but only those where it was immediately obvious that certain references are missing. More thorough work is required by the authors.
The authors have added the suggested references.
- In parts, the manuscript still mentions complex physical/engineering terms and concepts without further explanation. Being an experienced micro-CT user myself, but with a biology background, I still had difficulties understanding several more technical text sections. The text should guide the user through these more technical parts carefully. My advice here would be to ask a colleague with little or no expertise in micro-CT to go through the text and identify those difficult sections or terms.
A zoologist read the manuscript and relevant comments were addressed.
- Much weight is given to the example (the three-horned chameleon) in several sections (title, abstract, keywords, and discussion of the example). In my opinion, while it is useful to have an example, it is just this - an example for imaging to illustrate the actual purpose of the paper, and

could have been done with any object. The aim of the paper is not to conduct a biologically meaningful analysis of a specific organism, so the (understandable) enthusiasm over the object which can be discerned throughout the text should be reduced (see suggestions in specific comments) to focus on the actual aim of the paper

Relevant changes were made to the manuscript in order to remove the focus from the chameleon, but rather shift it to the principals and workings of CT.

- Use one of the available terms (e.g. micro-CT, X-ray micro-CT, laboratory X-ray CT) throughout the text.

The necessary changes were made to keep the term "micro-CT" was consist throughout the text.

Specific comments:

Title:

- I would remove the part "using a three-horned chameleon as an example" from the title for reasons outlined above

The title was changed to: "Laboratory X-ray micro-computed tomography: a user guideline for biological samples"

- The previous version had the phrase "user guide" in the title - which I find more useful and catchy as "a generalized approach". This is a kind of user manual, after all, and mentioning it in the title will attract more readers.

The title was changed to: "Laboratory X-ray micro-computed tomography: a user guideline for biological samples"

Abstract:

- The abstract is rather narrative and should be more concise and avoid phrases like "fascinating", "It is important to note". As this is a user guide, a more detailed description of the contents could be given, e.g. mention that an introduction is given, scanning setup, reconstruction and visualization options are presented and their background explained, etc etc, and that finally an example is given. This example should not be mentioned in more than one sentence - it is an example to explain / guide the user through a scan and in the context of this paper does not convey any biological meaning. Overall, the abstract will need to be re-written to reflect the contents of the paper more precisely.

The abstract was revised and now reads: "This paper provides a detailed "how-to guide", describing many important concepts for users of laboratory X-ray micro-computed tomography (micro-CT) facilities. An introduction to micro-CT is given, followed by a background to computed tomography (CT), as well as relevant set-up, scanning, reconstructing and visualisation considerations. A three-horned Jackson's chameleon is presented as a biological sample, scanned with both a micro-CT, as well as a nano-CT scanner. Full data sets (projections, together with image stacks) that accompany the descriptive analysis are given as supplementary information, as the aim of this paper is to provide a platform for new users to gain experience in working with typical micro-CT data. Furthermore, the technical detail and discussion is relevant to all commercial types of micro-CT instruments."

Keywords:

- Industrial CT is probably not a keyword many users will use, especially not biologists (or those focusing on scanning biological samples). Consider replacing it or removing it.

"Industrial CT" was removed as a keyword.

- Remove "three-horned chameleon" or replace with the scientific name.

"Three-horned chameleon" was removed as a keyword.

- "non-destructive testing" should be "non-destructive imaging" or "non-destructive analysis"? Although the term exists, it refers to mechanical / engineering applications. Users with a biology background would much more likely search for "imaging" or "analysis" than "testing".
“non-destructive testing ”was changed to “non-destructive analysis”.
- consider adding "user guide" or something similar to the keywords, unless you decide to include it in the title.
We decided to include “user guide” in the title.

1. Introduction:
- Line 28: "...has been given to find techniques..." -> "...has been given to finding techniques..."
This was accordingly changed.
- Lines 32- 36 basically say the same thing twice - can be shortened.
The paragraph was shortened and now reads: “This method involves the recording of two-dimensional (2D) X-ray images from various angles around an object, followed by digital three-dimensional (3D) reconstruction. Thereby, the data from CT results in a virtual rendering of the object under investigation. This allows one to virtually travel through the volume in any direction and angle, view selected internal features, or make dimensional or volumetric or other more advanced three dimensional measurements [2, 3]. “
- Line 44: I agree with the definition of nano-CT for imaging at sub-micron resolution. This is repeated (in part) in the paragraph lines 202-207, however, later in the paper (lines 730 and following) you refer to resolutions around 5-10µm as nano-CT (or being scanned with nano-CT / overlap between nano-CT and micro-CT). This may be specific to your scanner models with are termed as such? I am familiar with scanners that routinely scan at 1-10µm resolution and are termed micro-CT. This should be clarified - maybe just by explaining (possibly in the paragraph lines 202-207) that terms are not as fixed as they may seem and that they may depend on the specific model of the scanner?
A sentence was added to the last paragraph of Section 3.1.2, reading: ‘But it should be noted that some instruments may have overlapping resolutions and that the terms (micro and nano) are not fixed and could differ depending on model types.”
- Line 47 and following: "potentially much improved and variable" - sounds strange, rephrase. Also, it does not become clear how the revolving sources vs. the revolving sample influence resolution. I suggest removing it, or rephrasing. The revolving source/sample could also be mentioned as another difference between systems.
The second paragraph of the Introduction now reads: “Other terms in general use are X-ray CT (XCT), industrial CT, µCT and laboratory X-ray tomography. Industrial CT differs from medical CT in a couple of ways: the resolution is potentially better in micro-CT systems, as well as having the source and detector fixed and stable with the sample rotating (in contrast to medical CT where the sample is stationary and the source and detector move around it); and lastly, industrial CT vary greatly with regards to voltages, currents and beam filtration.”
- Line 50: "dose-limited" and "beam filtration" are not easily understood for users not familiar with the topic. Maybe omit or rephrase keep more general here (don't go into detail though).
These changes are also included in the second paragraph of the Introduction as shown above.

- Line 54 and following: the explanation of the differences between synchotron and lab-Xray are not helpful for the reader - it would be more useful to explain that the one produces monoenergetic x-rays, whereas common targets (such as tungsten) in micro-CT instruments typically produce a broad spectrum of x-ray energies, as this is the basis for the use of different energies for different samples and the need for filters. The term "X-ray brightness" is not understandable to a non-expert here.

This section has been changed and now reads: “These systems should not be confused with synchrotron CT, where synchrotron radiation is the X-ray source (producing mono-energetic X-rays), as opposed to broad spectrum energies as produced by laboratory generated X-ray radiation [4, 5].”

- Lines 64 and following: Here you could add a few more references to important or exemplar papers, especially to the field of biology, as the mentioned review is from 2012. As far as I know, no newer review is available for all biology, but some comprehensive papers have been published in both zoology and botany using micro-CT.

More recent references have been added and the paragraph now reads: “Its potential as a tool in taxonomy has been recently demonstrated [10]. Another two good examples of micro-CT data sets are those of earthworms [11] and brittle stars [12].”

- Lines 68 and following. Remove/replace "mystery" and "de-mystifying" (non-scientific language). In addition, I think that the "mystery" of micro-CT is not one of the main reasons for underutilization, it's the lack of access to facilities and /or lack of funding (since often micro-CT services are costly, especially since biodiversity researchers/taxonomists) do often not have funds available to either buy and maintain a scanner or outsource the analyses. You may want to re-consider the first sentence of the paragraph. In addition, if you mention staining, you should cite Brian Metscher's works as these are considered the most comprehensive and very useful papers on staining biological samples, and there is a comprehensive work on staining by Pauwels et al (doi: 10.1111/jmi.12013). Similar "how to" guides exist also for plant studies (e.g. Staedler et al 10.1371/journal.pone.0075295).

“Mystery” and “de-mystifying” was removed.

The following sentence was added to incorporate the references on staining: “Additionally, Metscher’s [11] and Pauwel et al.’s [12] works are considered very helpful on staining biological samples.”

<u>2. Background to CT:</u>

- Line 92: "The fast moving electrons hitting a metal target material creates X-rays" → "...create X-rays"

This was accordingly changed.

- Line 93: "the resulting cone beam" - for a non-expert, this may need explanation. Either remove the term or explain it (either here or in conjunction with explaining edge artefacts / cone beam artefacts or with why to choose only central part of detector).

The term was removed.

- Line 105: "...with discussion" → ".. with a discussion"

This was accordingly changed.

<u>3. Computed tomography basics:</u>

- Line 135: Please cite here also other works on staining, e.g. Metscher, Pauwels, others - for many biological samples staining is crucial to obtain satisfactory results, and there is excellent literature on this.

Metscher and Pauwels were added as references to this section.

- Lines 136 and following: The common reason for staining is to be able to scan it in a liquid, as many soft-tissue samples in a liquid preservation medium cannot be dried, as this would destroy the sample and distort their morphology. Chemical, freeze drying or critical point drying may be used for those specimens in some cases, but may make the specimen brittle and fragile, again risking damage during handling. In these cases, staining increases the contrast of the specimen compared to the surrounding medium and the scanning container, so even if the specimen is held in place by the walls of the container (see line 156), both liquid and container can usually be

rendered transparent in the final visualisation. Please remove - or better - explain the drying step from your explanations and explain the reasons for staining in more detail. I also think that the statement that staining should only be used if "all other options have failed" is misleading. There are specific reasons for staining, and the ideal contrast method - staining or drying or none - depends on a variety of factors, including the characteristics of the specimen and the scope of the study, thus staining is not a "last resort" method.

The first paragraph of section 3.1.1 was revised and now reads: “Amongst analytical techniques, micro-CT requires very little, if any, sample preparation. Generally, a sample can be scanned exactly as provided, without any sample preparation. However, some soft-tissue samples are preserved in a liquid and will damage if removed, therefore requiring scanning in the liquid as such. In these cases, staining increases the contrast of the specimen compared to the surrounding medium [4, 12, 11].”

A further section was also added in this regards: “It should be noted that in particular cases samples need to remain in their ethanol filled jars or be transferred to plastic container completely filled with ethanol when being scanned. In such cases, too much damage will be inflicted on the samples if handled and wrapped in clothes.”

- Lines: 193/194: Explain the reason behind artefacts from the edges.

The following sentences were added to explain the resons behind the artifacts: “This is due to two reasons: firstly, the cone beam has reduced intensity near the edges and secondly, the cone beam geometry results in non-ideal reconstruction away from the central slice. For both these reasons, it is best to use the middle of the detector to minimise artifacts and reduced contrast near the edge as compared to the middle of the detector.”

-Lines 202-207: The lower limit of 500 nm for the nano CT may be instrument-specific to the Phoenix Nanotom. Several other nano-CT models (Xradia, SkyScan) have a higher resolution (around 200-50nm), and those maximal resolutions should be mentioned, as they may be of interest for people. In this context, it would be also beneficial to explain the differences between voxel size and resolution (e.g. looking at specifications of instruments on websites you may find both information on voxel/pixel size as well as on resolution). For a new user, or someone who needs to decide which instrument to buy, this is important information. The information in paragraph 3.1.3 is not too helpful in this context.

The following text was added in order to provide more useful information: “Best voxel sizes for the General Electric Phoenix Nanotom is 0.5 µm, the Zeiss-Xradia have models Versa (0.5 µm) and Ultra (0.05 µm) and the Bruker SkyScan 2211 is 0.1 µm. Actual resolution depends on sample size and in scan conditions, and few direct comparisons have been made between these instruments.”

- Line 216: "This value does not consider the actual spatial resolution capability..." and "X-ray spot size" Explain what "spatial resolution capability" and "X-ray spot size" mean and how they are related, so that a user can judge (e.g. when buying an instrument, or when deciding whether certain small features in the sample will be displayed satisfactorily) whether the instrument's capabilities are suitable. These are complex facts and difficult to explain - maybe an example or a small graph may help? This paragraph is important, and good explanation are rare in literature, so should be explained well and will be of help to many users.

More explanation was added as the new sentences to the paragraph reads: “This value does not consider the actual spatial resolution capability of the scan system. For example, if the X-ray spot size (focused X-ray spot from the source) becomes larger than the chosen voxel size, the spatial resolution of the system becomes poorer. That means less details are detectable, despite a good voxel size, due to the actual resolution which is not optimal.”

As well as adding this sentence to the end of the paragraph: “The only way of testing this is to image a small feature of known dimensions and ensure the feature is visible in the CT slice image.”

- Line 238: " have typical image acquisition times" → " may have image acquisition times from a few hundred ms to up to several seconds" (the exposure time really varies a lot depending on scanner model, voltage and filter used, so I think raw numbers are not helpful here.).
This was done accordingly.
- Line 248: "Averaging reduces noise" → " This averaging reduces nose" (term "averaging" has not been mentioned as such to explain the previous sentence, so add "this just for clarity)
This was done accordingly.
- Line 262: "on the detector in width multiplied" → add a unit to width (pixels, I guess)
“pixels” was added as a unit to width.
- Line 262 - Guideline: I have seen the formula N = width * π (thus, 3.14, not 1.6) to calculate the number of projection images. However, I am not familiar with the background to this, so I don't know whether one or the other can be used under certain circumstances (e.g. full vs. half rotation). Please just double-check that this is correct.
Has been checked and confirmed as being correct.
- Line 263: add comma after consequently
This was done
- Line 270: add comma after In fact
This was done
- Line 283: Remove the explanation about beam hardening here (line 283 until end of page) and just refer to the section on artefacts (i.e. ".... causing noise and artefacts (see section 3.1.6 for detailed explanations)")
This paragraph was changed accordingly and now reads: “Beam hardening is the most common CT artifact, causing noise and artifacts (see section 3.1.6 for a detailed explanation).”
- Line 290 and following: very narrative, verbose style. Rephrase or remove (remove parts on beam hardening certainly, as these should be treated in the section on artefacts).
The third paragraph of section 3.1.5 was rephrased and changed according to reviewer’s comments and now reads: “To illustrate the negative effect of using too high voltage during scanning, an example of a biological museum specimen fitted with a metal name tag, can be used. Subsequently, very poor contrast will be obtained between the different materials and it would be advised to remove the metal name tag before scanning as the metal is much denser for X-ray to penetrate than that of the sample itself.”
- Line 308: weird phrasing "the smaller the sample the lower the voltage that is possible". Rephrase to be more concise
The line was changed accordingly, and now reads: “in general: small samples require low voltage.
- Line 312 and following: Explain which filters are used for what - just explaining that aluminium is a "popular choice" is not very helpful.
The line was changed accordingly, and now reads: “Frequently used beam filters include 0.1 to 2 mm of copper and 0.5 to 1.5 mm tin or combinations of these, as well as aluminum, all used for beam filtration.”
- Line 315: "relevant to very dense objects and not biological samples" → biological samples can be dense, too (bones, calcified tissues) and may require up to 100kV, where a filter should - at least in certain scanners - certainly be used! Please remove / rephrase the sentence or put it into context - at the moment it sounds as if filters are not needed for biological samples.
This sentence was removed.
- Line 316: "Detector filtering": you did not mention /explain the opposite (beam filtering), it is only assumed to be what you describe in general. Maybe start the paragraph with describing the general two setups of filters: either filter between source and object, or between object and detector (and use the correct terms for each, as you refer to them later again) and what are the differences between them, and the effects each may create. "Secondary X-ray emission" and

"scattering" are not explained here and not understandable to new users, so please try to explain better.

This section was changed and now reads: “Detector filtration, the second type of filter setup, can also be used to reduce noise if, due to the denseness of the object, secondary X-ray emission is produced. This may happen when a dense material strongly absorbs X-rays and re-emits lower energy X-rays by fluorescence, or when a large amount of scattering is present from nanostructured samples causing X-ray scattering. In both cases using a filter after the sample and before the detector shields the detector from low energy X-ray emission and scattering, limiting noise.”

- Line 324: "Calculate the sample's minimum penetration..." → Explain how this is done: automatically by the machine? by the user? Should the user refer to the manual to check how the scanner model does this? As the sentence stands, a new user will not understand what needs to be done and thus this advice is not helpful.

The words “the user” was added to the sentence for clarity, and now reads: “i. A typical setup method to find best settings for a particular sample type, is to rotate the sample until its 2D X-ray projection image shows the darkest region (its longest or densest axis) and then the user can calculate the sample’s minimum penetration ratio compared to the background X-ray intensity (using the grey value counts measured in the X-ray image).”

- Guidelines IV: You mention minimum 10% explicitly in ii, then repeat in iv again the limits - maybe follow same style: if minimum is below 10%, raise voltage, if above 90%, lower voltage. Otherwise there is partial overlap.

Point ii”, after adding “whereas, if it is above 90% the voltage or current should be lowered” now reads: “If the penetration value is less than 10%, an increased voltage or current is required, whereas, if it is above 90% the voltage or current should be lowered.”

- Lines 345 and following: The part on hardware misalignment is not well explained here - why does this lead to the specific artefact?

The following sentence was added for clarity: “This misalignment causes a reconstruction error since the reconstruction process assumes the rotation axis to be perpendicular to the beam direction.”

- Paragraph on artefacts: There are publications on CT artefacts, you may want to cite them (e.g. Barret and Keat, "Artifacts in CT: Recognition and Avoidance", or Boas and Fleischmann, "CT artifacts: causes and reduction techniques").

The authors included the recommended references to the respective paragraph: “The reader is also referred to relevant publications on CT artifacts by Barrett and Keat [18], as well as Boas and Fleischmann [19]. “

- Line 366: Beam centering is not a (manual) option in every scanner model (or maybe named differently). Rephrase accordingly, or refer user to manual, or mention that this may be done automatically.

A sentence was added to stipulate that beam centering is usually an automated procedure and the paragraph now reads: “It is also necessary to run a beam centering right before scanning to ensure the electrons are well focused resulting in optimal X-ray output. This is an automated process in most commercial systems.”

- Lines 375 and following: It may be helpful to explain how these fast scans can be achieved. Which parameters should be changed to achieve a faster scan?

The following sentence was added as explanation about fast scan parameters: “Faster scans are achieved by using less images, no averaging and shorter exposure times.”

- Lines 394 and following: Please remove mention of scanner-specific built-in software (not useful for users with another scanner) and provide URLs for the independent software so users can obtain additional information

Reference to scanner-specific software was removed and URLs for independent softwares were added. This paragraph now reads: “Commercial micro-CT systems all have built-in reconstruction software packages with slightly different available settings, but all based on the same algorithms. Volume Graphics (http://www.volumegraphics.com/) is a stand-alone software package mainly used for 3D image analysis, but also offers a module for reconstruction, and another commercial standalone software for reconstruction is offered by Inside Matters (https://insidematters.eu/), called Octopus Reconstruction.”

- Line 405: "A choice of data type can be made" → "A choice of data type for the output"

This was changed accordingly.

- Lines 407 and following: This option is not really clear to me. Could you rephrase?

Small changes were made to the sentences, which now reads: “Then, the exact location of the rotation axis in each projection image is found by making use of an automated algorithm, which finds the central pixels in all 2D X-ray images – the use of the exact rotation axis in the back-projection algorithm improves the quality of the reconstruction and is especially important at higher resolutions.”

- Lines 428 and following: Option is not available in all scanners, rephrase to "it may also be possible" or something similar. Please explain scattering (if you have not done so before in the text, in the revised version). Add comma after "scattering is present".

The respective sentence was rephrased to “it may also be possible”.

Scattering was explained under section 3.1.5: “causing X-ray scattering (deflection or diffusion of X-ray particles).”

A comma was added after “scattering is present”.

- Lines 436: I have never seen this "bright ring", so it may be scanner-dependent. Please rephrase accordingly

The words “for some scanners” were included in the sentence.

- Exchange order of last two paragraphs in section 3.3.1 ("In medical CT..." and "It is also possible...").

The order of the two paragraphs was exchanged.

- Add to the calibration section that some scanners allow calibration to HU units by using calibrated specimens.

A sentence was added to the paragraph to incorporate this information: “In medical CT, each voxel is typically associated with a calibrated Hounsfield Unit (HU) or CT number, which is regarded as the average attenuation in that section of the sample [21]. Some scanners allow calibration to HU by using calibrated specimens.”

- Line 455: Using 180° scans is not a "seldom used option". In fact, I know labs where 180° is the common setting, and 360° is only used if extra detail is needed or if the sample is dense. You may want to explain in which cases either option can be useful.

This section was rephrased and now reads: “Another option is to reconstruct scans of less than 360 degrees (usually 180˚), when a sample is too large to rotate fully in the system used, for example.”

- Lines 456: Example for a redundant phrase "it can be mentioned here that in the case of data collection, images may become corrupted...". Please check the ms for such styles and rephrase them, as they are tiresome to read and blow up the text unnecessarily.

This sentence was corrected and now reads: “During data collection, images may become corrupted or lost. A simple process...”

Other similar phrases were also corrected.

- Line 461: Table 1 may be better placed after the artifacts section, not after the reconstruction section.

Table 1 was moved after the artifacts section.

- Line 485: maybe add another example besides IrfanView, and give URLs
Adobe Photoshop was added, along with URLs and the sentence now reads: “High quality image viewing programs such as Irfanview (http://www.irfanview.com/) and Adobe Photoshop (http://www.adobe.com/) are required.”
- Lines 487 and following: First few sentences are unclear to me. I believe they are meant to explain the section on scale bars, but they seem a little confusing to me. Maybe rephrase to express the main point more clearly?
“and each slice image is spaced by the voxel size (evenly distributed)” was added to this sentence in order to clarify the meaning thereof.
- Line 498 and following: Start by explaining that CT data can be 3D- visualized in two different ways: volume rendering and surface rendering, explain the one and then the other, not by stating what volume rendering is *not*, introducing a term (CAD data) which the user is not likely to know either. In the list of softwares there are some double mentions. Add Amira, which is a very commonly used software. Blender is open source, too. You may want to differentiate between freeware and commercial, not necessarily open source (users may be more interested in the costs). Give URLs for all packages.
This section was changed and now reads: “Micro-CT data can be visualised in two different ways, i.e. volume rendering, as well as surface rendering. Volume rendering is typically done in a 3D data analysis software package and involves iso-surface views using a user-defined threshold value, or a user-defined greyscale gradient for more advanced 3D rendering algorithms. These differ from 3D Computed Aided Design (CAD) software in that they handle full voxel data, i.e. data exist everywhere in a 3D voxel grid, not only on surfaces of the object. In other words, CAD software packages use triangulated mesh data of surfaces only (point locations only), while full CT data comprises data at every point in 3D space (grey value at every point). Therefore, a volumetric data set is significantly larger and requires more intensive computing power, even for simple visualisation. Commonly used commercial software available for volume rendering include Avizo (https://www.fei.com/software/avizo3d/), Volume Graphics VGStudio (http://www.volumegraphics.com/en/products/vgstudio-max/), Amira (https://www.fei.com/software/amira-3d-for-life-sciences/) and Simpleware (https://www.simpleware.com/), whereas surface rendering software are Blender (https://www.reddit.com/r/blender/), SolidWorks (http://www.solidworks.com/) and Autodesk (http://www.autodesk.com/). Additionally, freeware (or open source) software, which can be used for analysis of CT data in 2D or 3D, include ImageJ (http://imagej.net/), MIPAR (http://www.mipar.us/), Blob3D (http://www.ctlab.geo.utexas.edu/software/blob3d/), Quant3D (http://www.ctlab.geo.utexas.edu/software/quant3d/), Blender (https://www.reddit.com/r/blender/) and 3dma_rock (http://www.ams.sunysb.edu/~lindquis/3dma/3dma_rock/3dma_rock.html). The reader is referred to the paper by Walter al. [22] for additional information regarding software options that allow visualisation of micro-CT data.”
- Section 3.5. needs a general restructuring. It jumps back and forth between concepts and does not follow a clear line of thought. More specifically some points below:
Line 511 and following: It does not become clear towards what aim thresholding should be used. What is "image processing" in this context? Segmentation? Analysis? Visualization? To me, the paragraph reads more like a guide to applying transfer function to a volume-rendered object. In this case, the information should go under the previous paragraph "visualisation".
- Line 516: Replace "animal" with "object"/"sample"(also further down in paragraph)
- Line 516: Sentence "Aligning the slice image to the feature allows ..." is not understandable.
- Line 517: "Its selection was done..": What is "it" and use past tense? Are you referring to a specific example here? Rephrase or remove the whole following paragraph, looks as if it is misplaced, referring to "here", "animal", …

- Line 518: Region growing should be better placed under the segmentation options
- Line 527: "During image processing": image processing is a very general term. Which step are you referring to here? Replace "are initially used" to "can be used".
- Line 232: What are "the respective voxels"?
- Line 533: What is the binarization step here? Thresholding and segmentation is always binarization, creating selected and non-selected regions.
- Line 535: "Thresholding can be done in different ways" → merge with paragraphs above.
- Line 541: replace "colouring" with "selecting"
- Line 545: "significantly filter the data" is unclear

The above comments were all addressed in the paragraph that now reads: “The most widely used step in 3D image processing or segmentation is thresholding. This involves, in the simplest case, that a grey value is selected that will allow the viewing of all the brighter grey values in the respective volume when performing analysis. Also referred to as global thresholding, the same threshold will be used across the entire volume. Besides this simple thresholding step, individual features may be highlighted and analysed in detail with regards to its 3D location in the sample. Thresholding itself can be done in different ways, and the available methods depend on the software used and algorithms available. One valuable method is local adaptive thresholding, whereby a global threshold or region of interest is chosen as starting contour and local thresholding is then applied. In addition to thresholding and region growing, a very useful method especially for biological sample segmentation is manual segmentation using a drawing tool, to add or remove regions with a virtual pen or brush. This method is a tedious process as it can involve selecting in regions in every slice in the CT data set, but is sometimes the best way to segment complex biological data sets. This process can be accelerated by making use of thresholding or other semi-automated segmentation tools and then refining these selections using the drawing tool (instead of making the entire selection only with the drawing tool). Another possibility is to strongly filter the data, which simplifies the automated segmentation tools, but can result in loss of quality for small features. This is useful when only viewing is required, and not complex dimensional or volumetric measurements. A basic typical image processing procedure is described below, but more details of further segmentation and image processing methods are described in Mathews & Du Plessis [23], including a step-by-step guide for the segmentation of frog bones taken from whole frog scans.”

- Lines 546/547: double use of "required" - rephrase sentence.

The double use of “required” was removed.

- Line 549: "Image analysis aims to achieve the extraction ..." → sounds strange, rephrase

This sentence was changed and now reads: “The next step usually involves image analysis, which aims to extract both qualitative, as well as quantitative data from the images or image sequences [6].”

- Lines 577 and following: "The fact that the same sample" → why should that be confusing to a user? It's a simple matter of choosing an adequate "zoom level", as in photography, not a strange concept. I suggest removing it.

This sentence was removed.

- Lines 581/ 583: Either 40 minutes or 1 hour. Confusing otherwise.

This section was removed.

- Lines 584 and following: The aim here is not to achieve a longer or shorter scan, but to scan at a different resolution, which, in turn, has an impact on the scan time.

This section was removed.

- I suggest rephrasing the whole paragraph lines 574 to 593 to make it more concise - at the moment it is confusing to read. Maybe a small table could be more helpful in summarizing the

impacts of resolution on scan time and quality of image. Remove empty and redundant phrases from this paragraph.

This section was shortened and now reads: “In order to capture maximum detail it could be considered selecting only a specific (smaller) region of a sample, opposed to scanning the entire sample, consequently achieving a higher resolution (and more detail). The sample is thus sectioned and also referred to as a ROI scan of a section of the sample, which will be described in the 3.8.6.”

- Line 593: "next section" - refer to specific section, since they are numbered

This was done – see point above.

- Lines 595 and following: does not really fit here - it has to do with resolution, but the transition is a little abrupt. Explain "correlation" of SEM and micro-CT - how can this be done? Do you mean combined? Are there workflows / examples / references for this?

This section was removed.

- Line 599: "apply some process to the sample" sound strange and very general. Check the writing style throughout the whole paragraph.

This section was removed.

- Line 612: "getting more scans done semi-automated is of interest" → check writing style.

This sentence was changed and now reads: “In a multi-user facility the systems are operational for long periods, and there is a need to streamline and automate the workflow.”

- Line 620: explain or rephrase "vertical-adjusted multiple scan" - not easy to understand for new users

The words “vertical-adjusted” were removed.

- Line 624 and following: I'd place this paragraph before the previous on batch scanning, as it has to do with maintenance / system not being available.

The paragraph was moved as advised.

- Line 624: "Misconception" is strange here - I don't think any reader will have any idea about hardware failures and thus won't have any misconceptions.

- Line 626: "... stopped until the system is stable again" - explain what is meant by "stable" - this is not a full hardware failure. The first two sentences may be put into a more logical sequence.

- Line 627: Sentence starting with "However" is unrelated to the previous one(s). This should be placed directly after the first paragraph (line 609). Generally, consider re-structuring the paragraph 3.7 to follow a clearer line of thought.

The section on maintenance was changed considerably, and now reads: “Although some X-ray systems can be relatively maintenance-free (usually those with sealed X-ray sources), most commercial X-ray micro-CT systems require significant maintenance, which must be considered in terms of financial implications. Benchtop systems require less maintenance than cabinet and walk-in systems, with annual services and replacement parts contributing roughly 10% (depending on the usage) to the purchase price of the system. Additionally, typical usage for a large cabinet based system requires approximately 10-20% down-time for repairs and maintenance, as well as consumable replacements. It should be noted that filament replacement is required approximately once a week.

Another consideration regarding micro-CT scanning is the frequency of hardware failures. Such failures are common when performing continuous-rotation scans. Consequently, failure of any kind (even of only one image) causes the entire scan to fail and therefore using stepwise rotation is advised. For these reasons, the availability of local technical support from a supplier is a major consideration, along with the system type. In general, the larger the system the more maintenance is required.

In a multi-user facility the systems are operational for long periods, and there is a need to streamline and automate the workflow. For this purpose, some suppliers offer automated sample loading (e.g. 12 samples pre-loaded in a rotating sample mount), automated choice of scan settings and even sending of emails or phone messages when a scan is completed (or failed). Automated batch reconstruction, as well as batch image processing and analysis is possible on multiple scan data sets done overnight, when using macros written by the user. A limitation is, however, that the procedure should be very simple as there is nobody to check the result. None the less, the most widely used method of getting optimal samples done in the available time span, is to load samples vertically and then scan each one sequentially. In this way, approximately 3-10 samples can be scanned overnight, depending on the scan parameters and assuming no hardware failures occurs during the scan time."

-Figure 6: Make arrows smaller or different - text is difficult to read.

This figure was removed from the manuscript.

- Line 644: Drying a chameleon at ambient conditions may be fine, as the skin is tough and it will not change morphology, but please mention that this may destroy the sample and distort morphology (and certainly anatomy).

This point was stressed by adding the following sentence: "Caution should, however, be taken that the sample is fit for drying as some samples will change morphologically during drying."

- Line 651 and following: "Since the sample was loaded at 45 degrees...": I cannot understand how a better voxel size can be achieved here by the tilt of the specimen. My experience has shown that aligning the specimen vertically and to the centre of the rotation stage will reduce the space needed during rotation, which in turn will allow a larger zoom / higher resolution, as the width of the sample on the detector is less. Please explain this reasoning.

The effect of the tilting is explained by the added sentences to the respective section, which reads: "A further improvement could be obtained by loading the sample vertically and allowing it to rotate within the edges of the detector, but this requires multiple scans. Therefore, a single scan optimal resolution is found for an elongated sample at 45 degrees."

- Line 656: "As the chameleon is a biological sample, no beam filtration was required" → This is not a universal guideline! More useful here would be to state that the combination of instrument, target, energy and the characteristics of the sample did not require a filter, or something like that. With 100kV I would certainly use a filter, probably even a Cu+Al (strongest filtration available).

This sentence was changed and now reads: "The combination of instrument, target, energy and characteristics of the sample did not require beam filtration."

- Lines 658 and following: What is the difference here between good penetration value but not high signal values? How did you evaluate this? And how/why does increase of current (independently of energy) help to improve this?

Higher current improves the total signal counts on the detector, while keeping the penetration ratio the same. This improves signal to noise ratio. It is already explained in the paragraph and reference is made to the definition of penetration ratio in section 3.1.5.

Explain also why higher quality would have been possible at lower voltage and the relationship to penetration values. All these tips are potentially very helpful but they need a little more background explanation to those physical relationships and terms so that a user on a different instrument can follow the same reasoning on their machine, where settings may be completely different.

The penetration values were relatively high, as mentioned, therefore reducing this ratio by lowering the voltage can increase image quality. However, as mentioned this increases scan time and could also lead to other artifacts potentially. We do not add more detail for this to save space as the other reviewer requested.

- Sections 3.8.2 - 3.8.4 could use a little more explanation for each step so that the user can actually understand the reasoning. Referring back to guidelines, as you did in 3.8.3.iii may be helpful.

More explanation was added and the respective sections now read: “Corrected the background by taking the sample out and creating a smooth background image”; “cropping to remove unwanted regions around the edges using the manual crop editor” ; “smoothed to produce a clean surface rendering using adaptive Gauss default image filtering”

- Shorten section 3.8.6 a little to remove narrative style. Example: the first few lines of the paragraph can be just shortened to "Without the need for physical sectioning, the head of the animal was scanned at a higher resolution of 30µ.....". Similarly, a more parsimonous style can be applied to the whole section. Reduce also explanations of figures (you have these already in the legends, so only a reference to figures are needed).

Without the need for physical sectioning, the head of the animal was scanned at a higher resolution of 30 µm (with a field of view of about 30 mm) compared to the full body scan of 75 µm (field of view 75 mm). The higher resolution allows smaller features (i.e. skeleton structures (Fig. (5)) to be visualised. To maintain a high resolution of 30 µm and scan the entire sample, an automated multiple-scan process could have been used. This entails a sequence of scans that are performed at different height positions across a vertically mounted sample. The multiple scans are then stitched together to form a large data set and it should be noted that it is a lengthy process

Insert Figure 5 approximately here

To obtain sub-micron resolution of the horn of the chameleon, physical sectioning thereof was needed. Images depicting the improvement in resolution (from 10 µm to 0.95 µm) are depicted in Figs. 6 (a) to (e). The 10 µm scan was done in a nano-CT instrument rather than the micro-CT as better image quality was expected, although most micro-CT models can also scan at a 10 µm resolution.

- Lines 730 and following: Other micro-CT models do not have problems at scanning at a 10µm and even higher resolution, so make clear that in your specific case you decided to use the available nano-CT, but that this is not a general guideline.

This point was addressed in the following new sentence: “The 10 µm scan was done in a nano-CT instrument rather than the micro-CT as better image quality was expected, although most micro-CT models can also scan at a 10 µm resolution.”

- Overall, the whole section on different resolutions could be shortened to a half-page paragraph, and images can be combined to a single one. This paragraph should just give the user an overview of the possibilities of different instruments / settings, but currently it would take about 3-4 pages in print, which I find a bit much.

This was done as shown above.

4. Summary

- I suggest removing mentions/explanations of the "interesting biological findings" from the summary, biological investigations are not the scope of the paper.

The summary was adjusted and now reads: “3D laboratory micro-CT is a fast growing non-destructive testing and analysis method in scientific research applications. The increasing accessibility of such instruments will lead to an increasing number of new discoveries in scientific research applications. The aim of this paper was to provide a focused “how-to” guide for new potential users to better plan their work, and understand how to best make use of this technology. A specific new case study was used as demonstration – the Jackson’s three-horned chameleon specimen, which was scanned at different settings, and the full data sets provided as supplementary information. These data sets are meant to be used to gain a better understanding for viewing and handling typical 3D data sets from the technique. Scans up to sub-micron

resolution resolved osteocyte structure in the horn of the chameleon. These observations demonstrate a typical multiscale investigation by X-ray micro- and nano-CT.”

Table 1:
- Stitching artefacts: the cause "sample too wide" is unclear. Stitching artefacts should only occur if the sample is too wide and you do an oversize scan (and these parts are stitched together), but then artefacts are a cause of software / algorithm problems.
This was corrected and the following cause was given: “Reconstruction algorithms when stitching sample that is too wide for a single scan.”
- In Beam Hardening the part on "Edge of sample seems brighter...." in the "cause" column is actually an effect - the cause is only the part on the insufficient penetration of the sample.
This was corrected.
- Histogram is shifted : The histogram is not mentioned/ explained anywhere in the text before, may merit a little more explanation.
Reference to histogram was removed and the following was rather given as the problem: “The image is very dark on materials of interest, with bright spots in places”
- Scattering is not mentioned / explained in text. Solution to this may not be available in all scanners.
The authors are aware that this option might not be available in all systems, and therefore suggest that other non-system-specific reconstruction software is used. The text therefore stays unchanged.

As the authors have fully addressed the points raised by the reviewers, we are optimistic about a positive decision on publishing this paper in *GigaScience*.

I would like to confirm that all co-authors have reviewed this latest manuscript and that they approve submission of this manuscript to *GigaScience* in its current format. This manuscript has not being submitted elsewhere.

I trust that you would find the manuscript in order and I look forward to hearing from you soon.

Kind regards
Anton du Plessis
