# Supplementary material for: Laboratory x-ray micro-computed tomography: a user guideline for biological samples
Source: Gigascience. 2017 Apr 17;6(6):1–11. doi: 10.1093/gigascience/gix027 (PMC5449646; doi:10.1093/gigascience/gix027)
Supplement: GIGA-D-16-00031_Revision_2.pdf [file gix027_GIGA-D-16-00031_Revision_2.pdf]

Manuscript
Click here to download Manuscript Manuscript_CB1.docx

Laboratory X-ray micro-computed tomography: a user guideline for biological samples

Anton du Plessis[1,2, a *], Chris Broeckhoven[3,b], Anina Guelpa[1,c] & Stephan Gerhard le Roux[1,d]

[1] CT Scanner Facility, Central Analytical Facilities, Stellenbosch University, Stellenbosch, South Africa

[2] Physics Department, Stellenbosch University, Stellenbosch, South Africa

[3] Department of Botany and Zoology, Stellenbosch University, Stellenbosch, South Africa

[a] anton2@sun.ac.za , [b]cbroeck@sun.ac.za, [c]aninag@sun.ac.za, [d] lerouxsg@sun.ac.za

* Corresponding author

Abstract

Laboratory X-ray micro-computed tomography (micro-CT) is a fast growing method in scientific research applications that allows for non-destructive imaging of morphological structures. This paper provides an easily operated "how-to" guide for new potential users and describes the various steps required for successful planning of research projects that involve micro-CT. Background information on micro-CT is provided, followed by relevant set-up, scanning, reconstructing and visualization methods and considerations. Throughout the guide, a Jackson's chameleon specimen which was scanned at different settings is used as an example. The ultimate aim of the paper is make new users familiar with the concepts and applications of micro-CT in an attempt to promote its use in future scientific studies.

Keywords: X-ray tomography, micro-computed tomography, nano-computed tomography, 3D imaging, non-destructive analysis

1. Introduction

In recent years, substantial effort has been made to try and improve current techniques for investigating the morphology of biological samples in a non-destructive manner. One of these techniques is computerized axial tomography (CAT) or computed tomography (CT), a method widely used for non-invasive imaging of the anatomy of the human body [1]. Computed or computerized axial tomography involves the recording of two-dimensional (2D) X-ray images from various angles around an object, followed by a digital three-dimensional (3D) reconstruction. The resultant 3D-rendered volume not only allows for the multidirectional examination of an area of interest (e.g. organ), but also permits dimensional, volumetric or other more advanced measurements to be made [2, 3].

Industrial X-ray computed tomography is a specialized form of CT scanning, meant specifically for non-medical applications (hence the term "industrial") and frequently involves resolutions in the micrometer (µm) range. The method is therefore termed micro-computed tomography (micro-CT) and in the case of sub-micron resolution, such methods are termed nano-CT or sometimes X-ray microscopy, as the resolution is similar to optical microscopes. Industrial CT differs from medical CT in a three important ways: (1) due to its medical application, the X-ray source and detector move around a stationary sample in medical CT, whereas in industrial CT, the X-ray source and detector are fixed around a rotating sample. This rotating sample design facilitates image resolution adjustment (e.g. higher image resolution for smaller samples); (2) industrial CT is more flexible than medical CT with regards to voltage and current modification, which allows for the set-up to be modified to suit a range of materials (e.g. higher voltage for dense materials); (3) the image resolution of industrial CT scanners is often higher than that of medical CT scanners. Resolutions of industrial CT scanners are generally in the range of 5 – 150 µm, compared to medical CT scanners having best resolutions of 70 µm. In contrast, most nano-CT scanners have resolutions down to 0.5 µm. It must, however, be noted that medical micro-CT scanners optimized for scanning small live animals are available and can obtain similar resolutions as industrial CT scanners.

Industrial CT has numerous applications and is useful in any scientific field where non-destructive analysis is warranted. The versatility of this technique is shown in the number of reviews that have been published recently, such as in food sciences [4], the geosciences [5], materials sciences [6, 7] and biological sciences [8]. In biological sciences, industrial CT has gained popularity in recent years due to its application in taxonomy [9], paleobiology [10], evolutionary and ecological biology [11]. In addition, Broeckhoven et al. [12] have recently proposed a protocol that makes use of industrial CT to obtain high-resolution images of live reptiles and amphibians.

Despite its numerous applications and potential, the use of industrial CT has not been maximized yet as researchers from biological sciences are often unfamiliar with the scanning process, including sample preparation, the scanning process itself and 3D reconstruction. Lack of knowledge could result in poor scan quality and/or inability to extract adequate information for the required research purpose or question. Here, we provide guidelines that can be consulted not only by new users with a general biological background, but also by CT operators that are unfamiliar with biological specimens. A multi-scale investigation of the Jackson's chameleon (*Trioceros jacksonii*) is used as an example throughout the guideline. Ultimately, our aim is to improve the efficiency of micro-CT facilities and biological research through an improved understanding of the capabilities and limitations of the technique.

2. Background to computed tomography

Micro-CT makes use of an X-ray source and detector to obtain 2D images of a sample which, in turn, can be combined to create a 3D reconstruction of that specific sample [13]. The fundamental components of any micro-CT instrument are (1) penetrating ionizing radiation, (2) a sample manipulator and (3) a detector [14] (Fig. 1). The basic principle of micro-CT is described in Kak and Slaney [15]. In summary, X-rays are generated by a micro-focus X-ray tube, which uses a beam of electrons accelerated by a voltage of up to 240 kV (or more in a vacuum tube), and are focused onto a tungsten or similar metal target. The interaction between the fast moving electrons and the metal target is responsible for creating X-rays. The X-rays are then directed through and around a sample, before being collected on a 2D X-ray detector in the form of a "shadow image", also called a projection image or radiograph [3]. In industrial CT, the sample manipulator (or rotation table) positions the sample in the path of the radiation beam and rotates it through a specific angle (usually 180 or 360°). The detector converts the attenuated radiation, which passes through the sample along a straight line, into the 2D digital images, consisting of thousands of pixels. In this way, many hundreds or thousands of 2D projection images are recorded during the scan process. After scanning, these images are used to reconstruct a 3D data set by making use of filtered back-projection algorithms [16]. Effectively, every volumetric pixel (or voxel) is imaged (by 2D projections) from many angles, and the sum of its view from every angle produces a representation of the actual X-ray density and hence brightness of that voxel [3]. Following reconstruction, a variety of software tools can be used for data visualization and analysis. These steps are all described below with a discussion of practical considerations.

Insert Figure 1 approximately here

3. Computed tomography procedure

The micro-CT procedure includes various steps such as (1) sample preparation and mounting, (2) scanner set-up and parameter selection, (3) scanning procedure, (4) image reconstruction and (5)

image visualization. We refrain from explaining the image processing and analysis as this is highly dependent on the software used, but suggest researchers make use of the program developer's user manuals. The set-up considerations are explained here together with three general guidelines (Guidelines I to III) which can be used to aid the scanning process. The entire micro-CT procedure will then be explained, where applicable, using a Jackson‘s chameleon (*Trioceros jacksonii*) from the Ellerman Collection at Stellenbosch University (specimen number USEC/H-2927) as an example. The sample was scanned using a Phoenix V|Tome|X L240 (General Electric Sensing and Inspection Technologies / Phoenix X-ray, Wunstorff, Germany) micro-CT system, as well as a Phoenix nanotom S (General Electric Sensing and Inspection Technologies / Phoenix X-ray, Wunstorff, Germany) nano-CT system, both located at the CT Scanner Facility of the Central Analytical Facility (CAF), Stellenbosch University, South Africa [17]. Full data sets that accompany the descriptive analysis are provided as supplementary information. These data sets can be used to obtain a better understanding of viewing and handling typical 3D data sets resulting from the proposed procedure.

3.1 Sample preparation and mounting

Micro-CT requires very little, if any, sample preparation and a sample can usually be scanned exactly as provided. Because of the rotating sample design of industrial CT scanners, it is important to load the sample correctly to avoid movement during scanning. Sample mounting involves the use of a low-density materials (e.g. cardboard tubes, plastic bottles or glass rods) which hold the sample in place on a rotation stage, but separates the sample from the dense rotation stage hardware. We suggest that samples are loaded at a slight angle to ensure that parallel surfaces to the X-ray beam are minimized (Fig. 2). The reason is that parallel surfaces are not penetrated properly and lead to image artifacts and lack of detail in the data set in the plane of the flat surface parallel to the beam.

As mentioned before, the most important factor is to avoid movement of the sample during scanning. Sample movement, for example if the sample is not properly secured in its holder, will inevitably result in a blurred 3D image which might not be suitable for analysis. Likewise, dehydration of a preserved or wet sample can cause shrinking and might result in a blurred 3D image. For obvious reasons, this is more relevant during longer scan times. Various approaches can be used to overcome the problem of movement or shrinkage. The most convenient approach is to dry the sample before scanning. However, as this technique is rather invasive, it is unsuitable for valuable or delicate samples, such as museum specimens and should be avoided unless the samples are not being reused. A more suitable method is to wrap the sample in a wet cloth (i.e. drenched in water, ethanol, formalin or isopropanol), thereby keeping the sample moist during the scanning procedure. Another possibility would be to scan samples inside liquid filled tubes. However, care must be taken that the sample is not kept in place by the edges of the container,

because these edges will not be separable from the sample during the image processing steps. It should be noted that some samples are too small or delicate to be removed or are prohibited from being removed from their containers and might need to be scanned therein. In these cases, staining should be considered to increase the contrast of the specimen compared to that of the surrounding medium. We refer to the studies by Mizutani and Suziki [8], Metscher's [18] and Pauwel et al.'s [19] for further information on soft tissue scanning and staining methods to enhance contrast. The choice of mounting method will often be determined by the museum to which the sample belongs. In this case, we suggest that museum curators weigh all the above mentioned options carefully against their disadvantages to ensure that researchers can easily and rapidly obtain scans with high image quality.

The mounting procedure for nano-CT scanning is similar to that of micro-CT scanning of very small samples. The sample is mounted on top of a small glass rod and secured with double sided tape, glue or can be placed inside a small cube of foam, fitted with a small cavity or slit and attached to the glass rod. A plastic film (e.g. Parafilm®) can be used to cover soft tissue or wet samples to avoid dehydration.

Insert Figure 2 approximately here

3.2 Scanner set-up and parameters

3.2.1 Sample size vs. resolution

Careful selection of resolution is the first major factor affecting a micro-CT scan. A useful guideline (Guideline I) when estimating the best possible resolution for a sample of known dimensions is:

i. The optimal resolution is a factor 1000 smaller than the width of the sample. For instance, a sample with a width of 100 mm has an optimal resolution of approximately 100 µm.

The above guideline is based on the standard practice of using only the central 1000 of 2000 available pixels of the detector to minimize possible artifacts from the edges. This is due to two reasons: first, the cone beam has reduced intensity near the edges and second, the cone beam geometry results in non-ideal reconstruction away from the central slice. For both these reasons, it is suggested to use the middle of the detector to minimize artifacts and reduced contrast near the edge. While most detectors have 2000 pixels, others have more pixels which allows for improved magnification for the same sample size. This, however, might introduce other problems including an increase in data set size and prolonged reconstruction times. It must be noted that it is theoretically possible to use all 2000 available pixels in the above example, resulting in a resolution of 50 µm. Nevertheless, besides the risk of artifacts from the edge regions, it can be challenging to

mount a sample perfectly central on the rotation axis to avoid movement out of the field of view during rotation.

3.2.2 Resolution, voxel size and X-ray spot size

The voxel size of a micro-CT image is dependent on the magnification and object size as described above. This is related to the distance of the sample from the X-ray source and the detector [4]. Voxel size and spatial resolution are two concepts that are often confused, since the voxel size is the size of a pixel in 3D space, i.e. the width of one volumetric pixel (isotropic in 3 dimensions). This value does not consider the actual spatial resolution capability of the scan system. For example, if the X-ray spot size (focused X-ray spot from the source) becomes larger than the chosen voxel size, the spatial resolution of the system becomes poorer. That means less details are detectable, despite a good voxel size, due to the actual resolution which is not optimal. Since most commercial systems limit the size of the X-ray spot to the required voxel size (or provide the user an indication of this), the actual and voxel resolution are usually the same, but this is not regularly tested or reported. It is possible to use resolution standards (such as calibrated-thickness metal wires) to confirm spatial resolution and some reference standards exist, although a generally accepted standard for industrial CT systems does not yet exist. It is therefore possible that the amount of detail that is detectable in a scan can vary considerably from system to system, or even between different scans from the same type of system. The quality differences are either due to improper settings that possibly result in large X-ray spot sizes, or to improper choice of other scan parameters. The sole way of testing the scan quality is to image a small feature of known dimensions and ensure the feature is visible in the CT slice image.

3.2.3 Scan time, number of images and rotational options

The major consideration for scan time is the acquisition time of single projection images, which can vary from system to system due to detector sensitivity and dynamic range differences, X-ray tube brightness differences, and differences in physical distance form source to detector [3]. A typical image acquisition time in a walk-in cabinet system with a 16-bit flat-panel detector is 500 ms per image, while some benchtop systems may have image acquisition times from a few hundred ms to up to several seconds per image. All systems have variable image acquisition times and therefore scan times can vary considerably. To obtain the highest possible scan quality, the full dynamic range of the detector should be explored. By doing so, the image contrast is maximized by raising the image acquisition time up to near saturation of the detector for a particular X-ray setting. If the image acquisition time is too low, the resulting contrast will be poor with grainy images in extreme cases.

Some scanners involve continuous scanning (i.e. continuous rotation and image acquisition without steps), but the discussion here is limited to a stepwise rotation for simplicity. At each step

position, one or more images can be acquired and averaged to provide an improved image quality compared to a single image per position. While the averaging method reduces noise and consequently improves image quality, its effect highly depends on the inherent noise of the detector used. For samples which might experience small vibrational movements during rotational movement (e.g. leaves or hairs), it is advisable to use the skip function (if available) because it ignores the first image acquired at each new step position (during which time the sample stabilizes). Since this vibration is due to the stepwise process, an alternative approach would be to use continuous scanning because it also reduces vibration. In this case, however, averaging is not possible.

The number of step positions required depends on the sample size relative to the magnification. Therefore, the higher the magnification and hence the number of pixels used on the detector, the larger the number of images required for a good reconstruction. A useful guideline in this regard (Guideline II) is:

i. *The number of pixels covered by the sample on the detector in width (pixels) multiplied by 1.6 equals the number of projection step positions required. Consequently, up to a maximum of 3200 step positions are used for a typical 2000 pixel wide detector*.

3.2.4 Scanner parameters

Voltage - X-ray voltage highly depends on the type and material composition of the sample. The most optimal material discrimination is usually obtained by using lower voltages. However, the X-ray penetration value (i.e. the percentage of detector counts around and through the sample) might be too low in case of dense material, thereby causing noise and artifacts. Beam hardening represents the most common CT artifact, causing noise and artifacts (see section 3.6 for more). Beam hardening occurs when the X-ray beam, which comprises a range of X-ray energies, encounters differences in absorption from different angles and along different paths through the object, either due to a very dense object itself or due to dense parts of an object. Different X-ray paths result in varying absorption of the easily-absorbed low-energy X-rays, and this results in either “cupping” artefacts in dense objects (brighter regions around the edges of the material) or streaky artefacts in dense parts of a larger object (especially for very dense parts, such as metal tags).

Filtration – Two filter applications exist: (1) the filter is placed between the X-ray source and the sample, or (2) the filter is placed between the X-ray detector and the sample. The first type of filtration, called beam filtering, is useful when the voltage is increased and a beam filter is added to pre-compensate for expected beam hardening. The filter effectively reduces the polychromaticity of the beam, thereby preventing streaky artifacts. Frequently used beam filters include 0.1 to 2

mm of copper and 0.5 to 1.5 mm tin or combinations of these, as well as aluminum, all used for beam filtration. The second type of filtration, detector filtration, can also be used to reduce noise if, due to the density of the object, secondary X-ray emission is produced or scattering is present. This may happen when a dense material strongly absorbs X-rays and re-emits lower energy X-rays by fluorescence, or when a large amount of scattering is present from nanostructured samples, causing X-ray scattering. In both cases, using a filter after the sample and before the detector shields the detector from low energy X-ray emission and scattering, limiting noise.

Guideline III is presented for the calculation of the scanner voltage and determining adequate penetration values.

i. *The following X-ray tube voltages can be used as a starting point: biological samples: 30 to 100 kV; small rocks and light metals: 60 to 150 kV; heavy metals and larger rocks: 160 to 240 kV or more; and in general: small samples require low voltage.*
ii. *A typical setup method to find best settings for a particular sample type, is to rotate the sample until its 2D X-ray projection image shows the darkest region (its longest or densest axis) and then the user can calculate the sample's minimum penetration ratio compared to the background X-ray intensity (using the grey value counts measured in the X-ray image). Penetration values from 10 % to 90 % should result in good scan quality. If the penetration value is less than 10%, an increased voltage or current is required, whereas, if it is above 90% the voltage or current should be lowered.*
iii. *If the X-ray detector becomes saturated as a result of (ii), beam filters can be applied to prevent saturation, while still increasing the penetration value. By making use of a beam filter, a higher voltage or current can be obtained with a reduction in the low energy X-rays such that the detector does not yet saturate.*

3.3 Scanning procedure

Prior to scanning, it is important that the background is normalized. Background normalization is achieved by removing the sample and using the X-ray beam at the chosen settings to correct for all intensity variations across the detector (i.e. the X-ray beam being more intense in the middle of the detector compared to the edges of the detector). This normalization procedure can be conducted prior to each scan, but is in practice only required if X-ray or acquisition settings change, or after a long period of scanner inactivity. In addition, it is necessary to run a beam centering prior to scanning to ensure correct focusing of the electrons, thereby ensuring the smallest spot and highest emission. In most commercial systems, however, this is an automated process. Once the sample is loaded and settings chosen, images can be acquired. The scanning itself is done automatically with no user interaction. Frequent supervision is advisable as several errors may occur including an unstable X-ray source (requiring a warmup) or filament burn

(requiring replacement). It is important to note that addressing these issues can take a considerable amount of time and this should be taken into account during data collection planning.

Although our proposed scanner settings are aimed at acquiring high image quality, it must be mentioned that it is possible to obtain a shorter scanning duration. This can be achieved by using less images, eliminating averaging and reducing exposure times. Fast scans (e.g. 5-15 min) might not be ideal but can be sufficient in some cases, for example when trying to identify a relatively large feature or when simple measurements have to be taken. Alternatively, they are also used as an exploratory method to find a region of interest prior to commencing a long, higher quality scan.

3.4 Image reconstruction

After all 2D image projections are obtained, a 3D volume can be constructed. The reconstruction process involves the mapping of each voxel by using projection image representations of that particular voxel from many angles. This mapping is done by a Feldkamp filtered back-projection algorithm [20]. Commercial micro-CT systems have built-in reconstruction software packages that might differ in settings, but are all based on the same algorithms. For example, Volume Graphics (http://www.volumegraphics.com/) is a stand-alone software package mainly used for 3D image analysis, but also offers a module for reconstruction. Another commercial standalone software for reconstruction is offered by Inside Matters (https://insidematters.eu/), called Octopus Reconstruction.

Reconstruction software involves a series of options, which might affect the quality of the obtained 3D data. These options will be described in general here, though reconstruction software packages might differ in their availability of the described options. Firstly, the field of view can be cropped to make the total reconstructed volume smaller. This helps reducing the data set size and reduces the duration of the reconstruction since less memory is required. This is especially helpful when time or computational power is limited. Secondly, the type of output file can be chosen, which is usually selected as 16-bit. Here, it is possible to select 8-bit if storage space or memory is limited. Thirdly, the exact location of the rotation axis in each projection image is found by making use of an automated algorithm which finds the central pixels in all 2D X-ray images – the use of the exact rotation axis in the back-projection algorithm improves the quality of the reconstruction and is especially important at higher resolutions. This process can also be coupled with a refinement process, correcting for small movement or shift of the sample and improve the edge clarity in the reconstructed data set. Next, beam hardening correction is to be considered. Beam hardening corrects much of the generally-occurring “cupping” effect in samples where the edges seem brighter than the middle of the scan. Another option called clamping, disregards a certain percentage of pixels that are “outliers” in terms of strong or weak absorption compared to the rest of the data and which effectively improves the grey value contrast in the images. Clamping can be

very useful when a small quantity of bright dense phases that are not of interest are present. The percentage of pixels that are clamped, and the clamping direction (lowest or highest grey values only, or both) can be set. Furthermore, it may also be possible to make use of special settings to select the background detector counts in each image and normalize this across the series of images, which is useful when scattering is present, resulting in brighter or darker projection images from different angles. It is possible to use special algorithms to remove ring artifacts by disregarding "dead" pixels from the 2D projection images. Ring artifacts especially near the center of rotation are also removed by making use of a detector shift process, whereby the detector shifts horizontally between step positions and which are corrected in the reconstruction process resulting in a smoothing of the rotational center artifact. It is clear that various options exist for the reconstruction of a data set, thereby making this process an important step which can help the user with obtaining improved image quality. Since the reconstruction process itself can vary significantly, it is suggested that the raw 2D X-ray projection images are retained after completion of the reconstruction process, as this will allow the user to improve the reconstruction of the same data in the future.

3.5 Image visualization

Micro-CT data can be visualized in two different ways, either by volume rendering or surface rendering. Volume rendering is typically conducted in a 3D data analysis software package and involves iso-surface views using a user-defined threshold value, or a user-defined greyscale gradient for more advanced 3D rendering algorithms. These differ from 3D Computed Aided Design (CAD) software in that they handle full voxel data, i.e. data exist everywhere in a 3D voxel grid, not only on surfaces of the object. In other words, CAD software packages use triangulated mesh data of surfaces only (point locations only), while full CT data comprise data at every point in 3D space (grey value at every point). Therefore, a volumetric data set is significantly larger and requires more intensive computing power, even for simple visualization. Commonly used commercial software available for volume rendering include Avizo (https://www.fei.com/software/avizo3d/), Volume Graphics VGStudio (http://www.volumegraphics.com/en/products/vgstudio-max/), Amira (https://www.fei.com/software/amira-3d-for-life-sciences/) and Simpleware (https://www.simpleware.com/), whereas surface rendering software are Blender (https://www.reddit.com/r/blender/), SolidWorks (http://www.solidworks.com/) and Autodesk (http://www.autodesk.com/). Additionally, freeware (or open source) software, which can be used for analysis of CT data in 2D or 3D, include ImageJ (http://imagej.net/), MIPAR (http://www.mipar.us/), Blob3D (http://www.ctlab.geo.utexas.edu/software/blob3d/), Quant3D (http://www.ctlab.geo.utexas.edu/software/quant3d/) and 3dma_rock (http://www.ams.sunysb.edu/~lindquis/3dma/3dma_rock/3dma_rock.html). We refer to Walter et

al. [21] for additional information regarding software options that allow visualization of micro-CT data.

3.6 Scan quality problems and artifacts

The diversity of available scanner options and settings can unfortunately be associated with various image quality problems and artifacts. Figs. 3 (a) to (c) show micro-CT slice images of the chameleon with metal streak artefacts present, too low voltage and too high voltage, respectively. In the first case, the streak artefacts reduce the image quality of the specimen, while too low voltage causes brightness variations around dense objects in the image and too high voltage results in poor contrast between materials. The above-mentioned illustrate some of the typical image quality problems that can occur during scanning. It is not only the scan process but also reconstruction that can affect the image quality as shown in three examples in Fig 3 (d) to (f). Fig. 3 (d) has poor contrast, in this case due to incorrect reconstruction setting (clamping). The same effect may occur when a sample is scanned with the metal rotation table in the scan volume. Fig. 3 (e) has a double edge, due to incorrect reconstruction setting (i.e. offset correction). This double edge can also occur if the sample moves during a scan, though to a lesser degree. Fig. 3 (f) illustrates a slight blur on the edges, which is due to sample vibration due to non-rigid mounting of the sample and stepwise rotation causing the sample to move slightly, more so on the top than the bottom of the sample.

Insert Figure 3 approximately here

Beam hardening has been mentioned before within the context of streak artifacts. However, samples with homogenous material density scanned with an insufficient voltage might also result in a “cupping” effect. This artifact arises when X-rays do not penetrate the sample sufficiently. Other artifacts and unwanted image effects include cone beam artifacts affecting the edges of materials near the edges of the detector, double edges due to tilt axis misalignment relative to beam axis, and blurring due to an unstable rotational axis. We refer the reader to relevant publications on CT artifacts by Barrett and Keat [22], as well as Boas and Fleischmann [23]. Additionally, Table 1 summarizes problematic micro-CT scans as discussed in this paper, providing the cause(s) and possible solution(s) to the problem.

Insert Table 1 approximately here

3.7 Example: micro-CT scanning of a three-horned chameleon

The considerations, guidelines and options related to micro-CT scanning of biological samples are presented here and could be used as guiding principles when conducting micro-CT scans and

analysis. The three-horned chameleon is used as an example and will follow the step-wise guidelines as presented in this paper.

1. **Sample preparation and mounting** - A preserved three-horned chameleon specimen was taken out of its preservation jar and dried out at ambient conditions for a few hours prior to being mounted on florist foam fixed on top of a cardboard tube (Fig. 2 (a)). Although this method might not be ideal for museum specimens (see 3.1) it was chosen to avoid imaging artefacts associated with movement during dehydration. The densest features of the chameleon can be seen as the darker regions of a digital X-ray projection image of the specimen (Fig. 2 (b)).
2. **Scanner set-up and parameters -** The total height of the sample was 2000 pixels, and using Guideline I, the best possible resolution that could be obtained was 75 µm. Following Guideline II, 3200 step positions were used. The sample was loaded at 45 degrees, because it provided a slight improvement in the best possible voxel size compared to horizontal or vertical mounting, for a single scan volume (vertical or horizontal would be limited to the longest axis of the chameleon sample). It would have been possible to load the sample vertically and scan at a similar resolution, but this would have required multiple scans. Averaging was set to 2 and skipping of the first image at each new position was used. Initially, a typical image acquisition time of 500 ms was set, resulting in a total scan duration of approximately one hour. Tube voltage was set to 100 kV, whereas the beam current was set to 100 µA. No beam filtration was used. This setting showed a good penetration value, but due to relatively low signal values on the detector the current was increased to 200 µA to obtain approximately 8000 counts, where 10 000 is the saturation level of the detector (Guideline III). In this process a trade-off between scan time and image quality was found. Higher quality would have been possible with more averaging, resulting in longer scan times. Higher quality would also have been possible at lower voltage since the penetration values were quite high. When lowering the voltage, the total X-ray emission from the source reduces, which requires a longer image acquisition time to allow the best possible contrast capable with the detector. However, this also increases scan time and additionally, lower voltages can cause unexpected artifacts as explained above.
3. **Scanning –** The background was corrected by removing the sample and creating a smooth background image. A beam centering was conducted, the sample mounted on florist foam was loaded and the image acquisition process was started. The process was monitored to correct for any errors.
4. **Image reconstruction** - Reconstruction settings used for the chameleon scan included: cropping to remove unwanted regions around the edges using the manual crop editor, selecting the 16 bit data type and correcting for offset by using a scan optimization

process. Additionally, a low beam hardening correction value and a background intensity value was used to correct for variations in intensity. The reconstruction process resulted in a single data file with a size of 6.3 gigabytes.

5. **Image visualization** - The 3D visualization of the chameleon is shown in Figs. 4 (a) and (b). A simple thresholding function (not explained here) allows for the visualization of the skeleton structure which is notably denser than the rest of the animal.

Insert Figure 4 approximately here

3.8. Scanning at higher resolution

As discussed in Section 3.2.1, the choice of resolution is perhaps the most important factor for data collection planning. Here, we briefly illustrate the differences between resolution settings using the chameleon as an example. Firstly, the full body scan (resolution: 75 µm) is compared to a close-up of the head scanned at 30µm. It is evident from Fig. 5 that a higher resolution allows smaller features (e.g. skeleton structures) to be visualized. As mentioned earlier a higher resolution (e.g. 30 µm) can be used to scan the entire sample with an automated multiple-scan process in which a sequence of scans are performed at different height positions across a vertically mounted sample. The multiple scans can afterwards be stitched together to form a large data set. However, it should be noted that this is a lengthy process.

Insert Figure 5 approximately here

Secondly, the horn of the chameleon was scanned after dissection to obtain sub-micron resolution. The improvement in resolution (from 10 µm to 0.95 µm) is depicted in Figs. 6 (a) to (d). The sub-micron resolution allows the user to obtain detailed information on, for example, the bone micro-architecture of a sample. The 10 µm scan was conducted using a nano-CT instrument, but it must be noted that most micro-CT models are able to achieve this resolution.

Insert Figure 6 approximately here

4. Summary

In this paper we aimed to provide a “how-to” guide for new users unfamiliar with micro-CT to obtain a better understanding of the technique. In addition we provided suggestions and guidelines which can be used during research planning and facilitate the interaction between researchers and CT operators and/or facilities. An example – the Jackson’s chameleon – scanned at various settings was used to illustrate the procedure and by making use of the guidelines, users can adapt the procedure to suit a variety of study objects or organisms.

5. Acknowledgements

We would like to thank A. Ziegler and S. Faulwetter for constructive comments on previous versions of this manuscript.

6. References

1. Kalender WA. X-ray computed tomography. Physics in Medicine and Biology. 2006;51(13):29-43

2. Brunke O. High-resolution CT-based defect analysis and dimensional measurement. Insight-Non-Destructive Testing and Condition Monitoring. 2010;52(2):91-3

3. Singhal A, Grande JC, Zhou Z. Micro/Nano CT for visualization of internal structures. Microscopy Today. 2013;21(02):16-22

4. Schoeman L, Williams P, du Plessis A, Manley M. X-ray micro-computed tomography (μCT) for non-destructive characterisation of food microstructure. Trends in Food Science and Technology. 2016;47:10-24

5. Cnudde V, Boone MN. High-resolution X-ray computed tomography in geosciences: A review of the current technology and applications. Earth-Science Reviews. 2013;123:1-17

6. Maire E, Withers PJ. Quantitative X-ray tomography. International Materials Reviews. 2014;59(1):1-43

7. Landis EN, Keane DT. X-ray microtomography. Materials Characterization. 2010;61(12):1305-16

8. Mizutani R, Suzuki Y. X-ray microtomography in biology. Micron. 2012;43(2):104-15

9. Faulwetter S, Vasileiadou A, Kouratoras M, Dailianis T, Arvanitidis C. Micro-computed tomography: Introducing new dimensions to taxonomy. ZooKeys. 2013;263:1

10. O'Connor PM, Sertich JJ, Stevens NJ, Roberts EM, Gottfried MD, Hieronylus TL, Jinnah ZA, Ridgely R, Ngasala SE, Temba J. The evolution of mammal-like crocodyliforms in the Cretaceous Period of Gondwana. Nature 2010;466(7307):748-751

11. Broeckhoven C, Diedericks G, Hui C, Makhubo BG, Mouton, PLFN. Enemy at the gates: Rapid defensive trait diversification in an adaptive radiation of lizards. Evolution, 2016;70(11):2647-2656

12. Broeckhoven C, du Plessis A, le Roux SG, Hui C. Beauty is more than skin deep: a non-invasive protocol for in vivo anatomical study using micro-CT. Met. Ecol. Evol. in press

13. Kotwaliwale N, Singh K, Kalne A, Jha SN, Seth N, Kar A. X-ray imaging methods for internal quality evaluation of agricultural produce. Journal of Food Science and Technology. 2011;51(1):1-15

14. Duliu OG. Computer axial tomography in geosciences: an overview. Earth-Science Reviews. 1999;48(4):265-81

15. Kak AC, Slaney M. Principles of computerized tomographic imaging New York: IEEE Press; 1988.

16. Lin C, Miller J. Cone beam X-ray microtomography-a new facility for three-dimensional analysis of multiphase materials. Minerals and Metallurgical Processing. 2002;19(2):65-71
17. du Plessis, A., le Roux, S. G., & Guelpa, A. (2016). The CT Scanner Facility at Stellenbosch University: An open access X-ray computed tomography laboratory. Nuclear Instruments and Methods in Physics Research Section B: Beam Interactions with Materials and Atoms, 384, 42-49.
18. Metscher BD. MicroCT for developmental biology: A versatile tool for high-contrast 3D imaging at histological resolutions. Developmental Dynamics. 2009;238(3):632-40
19. Pauwels E, Van Loo D, Cornillie P, Brabant L, Van Hoorebeke L. An exploratory study of contrast agents for soft tissue visualization by means of high resolution X-ray computed tomography imaging. Journal of Microscopy. 2013;250(1):21-31
20. Feldkamp L, Davis L, Kress J. Practical cone-beam algorithm. Journal of the Optical Society of America. 1984;1(6):612-9
21. Walter T, Shattuck DW, Baldock R, Bastin ME, Carpenter AE, Duce S et al. Visualization of image data from cells to organisms. Nature Methods. 2010;7:S26-S41
22. Barrett JF, Keat N. Artifacts in CT: recognition and avoidance 1. Radiographics. 2004;24(6):1679-91
23. Boas FE, Fleischmann D. CT artifacts: causes and reduction techniques. Imaging in Medicine. 2012;4(2):229-40

Figures

Figure 1. Photograph of the micro-CT scanner used during the study showing the fundamental components of the set-up. A typical micro-CT scanner consists of an X-ray tube (A) that emits X-rays, which pass through a sample (B) before being recorded by an X-ray detector (C).

Figure 2. Mounting of a Jackson's chameleon. Florist foam mounting material forms the basis onto which the sample is placed (a). A 2D X-ray projection image shows the very low density of the mounting material (b).

Figure 3. Micro-CT slice images of the chameleon illustrating the common artefacts. In (a) a metal tag is included in the scan volume, resulting in streaky artifacts (bottom right in image). In (b) an insufficient voltage was used, thereby creating image artifacts around the dense parts of sample. In (c) the voltage setting was too high resulting in poor contrast. In (d) poor image quality is caused by reconstruction clamping which was set too high. In (e) double edges are present due to incorrect offset calculations during reconstruction. In (f) slight blur is present due improper mounting.

Figure 4. Three-dimensional reconstructions of a Jackson's chameleon illustrating a surface view (a), and a semi-transparent view showing the skeleton in yellow (b).

Figure 5. A high-resolution (30 µm) scan of a Jackson's chameleon showing the skeletal elements present in the head.

Figure 6. Slice images of the horn of a Jackson's chameleon obtained by using nano-CT showing the bony core at 10 µm (a), 4 µm (b). At a very high resolution of 0.95 µm (c), the bone micro-architecture becomes clearly visible. A 3D rendering of the structure of the bony core inside the chameleon horn is visualized in (d).

Tables

Table 1. Summary of the various errors and artifacts discussed throughout this paper, stating the problems, possible cause and potential solution, respectively.

Problem	Cause	Solution
Grainy image	Image acquisition time too low	Increase image acquisition time
Streaky artifacts	Differences in absorption from different angles; X-ray penetration is insufficient	Increase voltage
Poor contrast	Too high voltage is used	Reduce voltage
Blurred image	Improper sample mounting; allowing sample to move during scanning	Proper mounting to ensure no movement during scanning
Stitching artifacts / vertical or horizontal line	Reconstruction algorithms when stitching sample that is too wide for a single scan	Make sub-sections of sample; use a smaller sample or less magnification
Beam hardening / cupping effect	Insufficient penetration of the sample	Reconstruction: use beam hardening correction option, or scan with higher voltage and more beam filters
Small movement or shift (double edge)	Inaccuracy of rotation stage or movement of sample	Reconstruction: do an offset correction; or rescan if offset cannot be corrected. Reset stages. Hardware could be faulty, e.g. tilt axis alignment
The image is very dark on materials of interest, with bright spots in places	Small quantity of bright dense phase are present, but irrelevant	Reconstruction: make use of the clamping option
Scattering	Causes brighter or darker projection images from different angles	Reconstruction: select background detector counts in each image and normalise across the series of images
Ring artifacts	Bright rings are visible in the top slice view	Reconstruction: make use of ring artifact reduction by disregarding 'dead' pixels from the projection image (or disregard pixels in the

		acquisition process)
Central rotation artifact	The center of rotation is visible as a line in a side slice view, or a dot with concentric rings from the top view.	Make use of detector shift option in acquisition, which smooths out the artifact.
Bright ring around outside of scan volume, resulting in poor image quality	In ROI scans where the sample extends over the side of the 2D image	Use special reconstruction algorithm which corrects for this, or crop the ROI further in reconstruction
Cone beam artifacts	Affecting the edges of materials near the edges of the detector	Use less magnification to fill less pixels on detector

 Click here to download Figure Figure1.tif

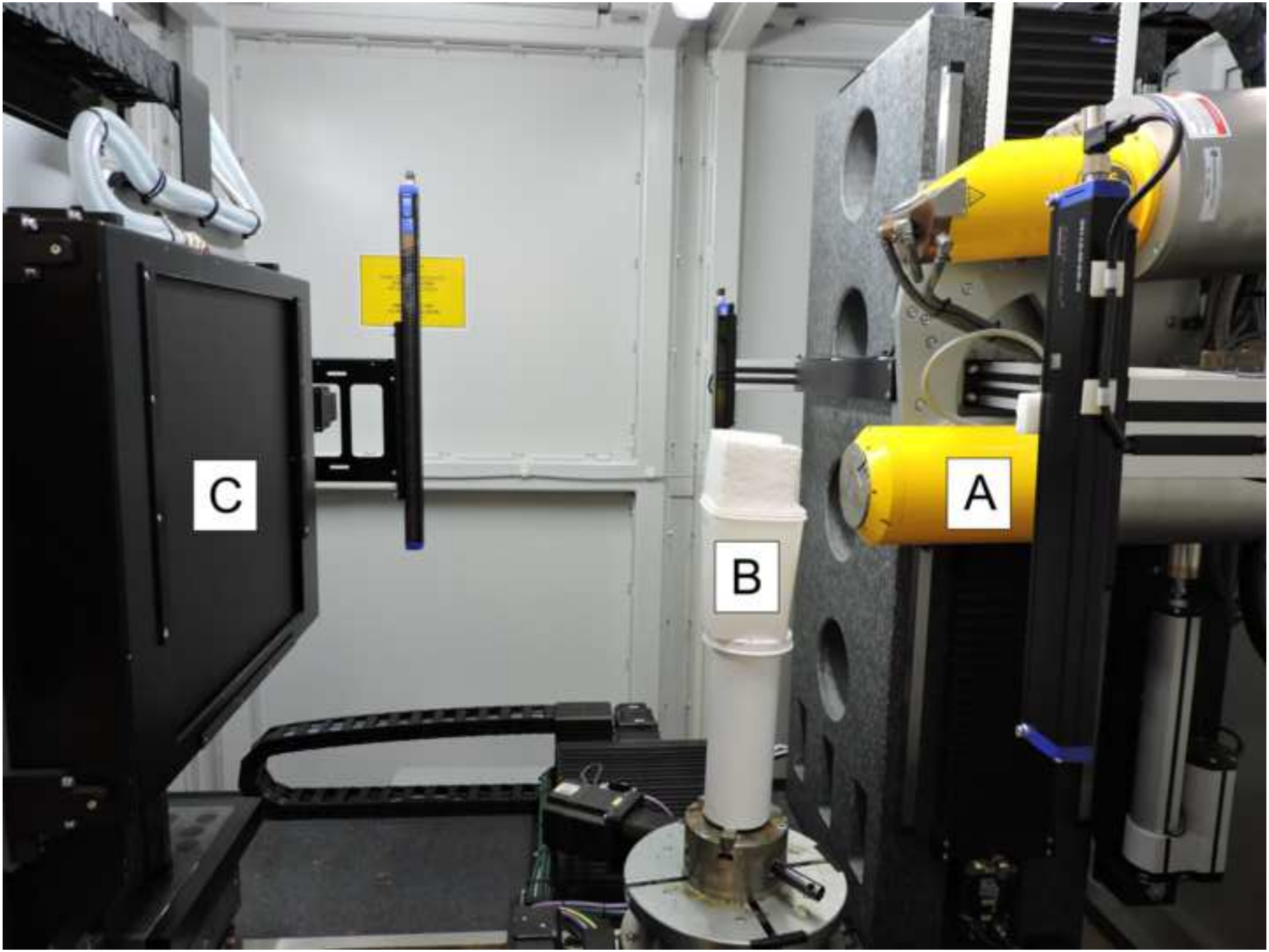

 Click here to download Figure Figure2.tif

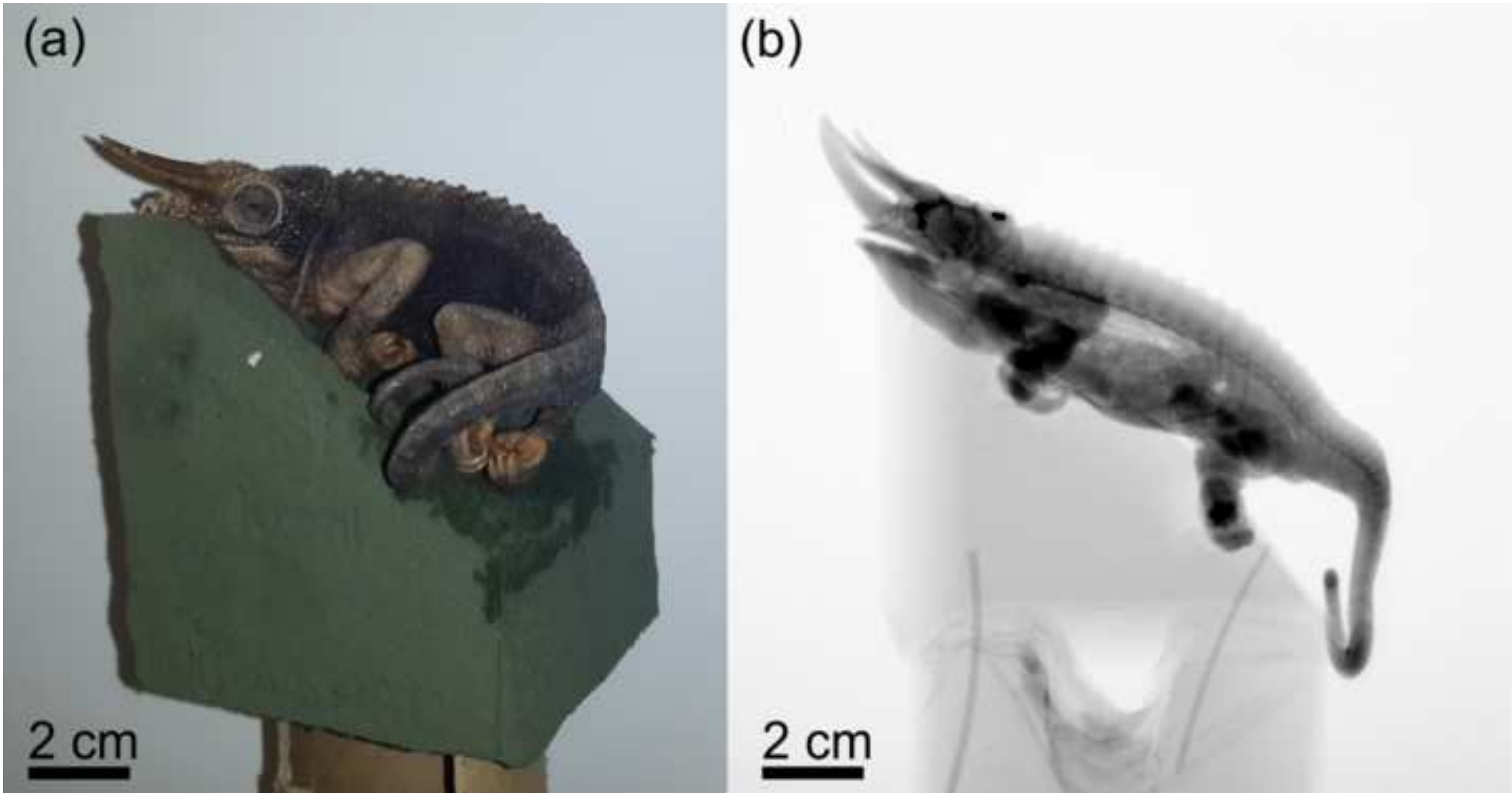

Figure

Click here to download Figure Figure3.tif

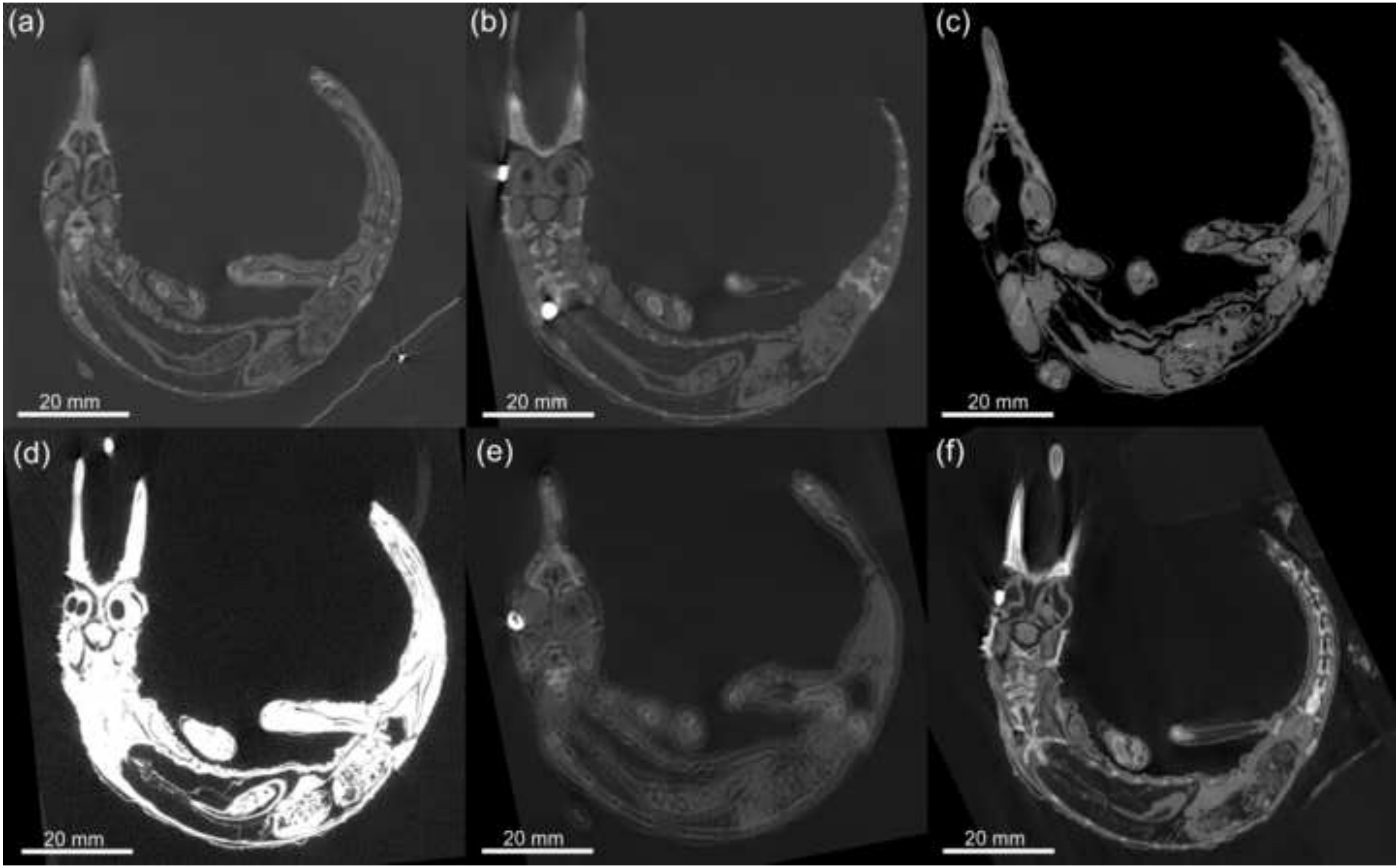

 Click here to download Figure Figure4.tif

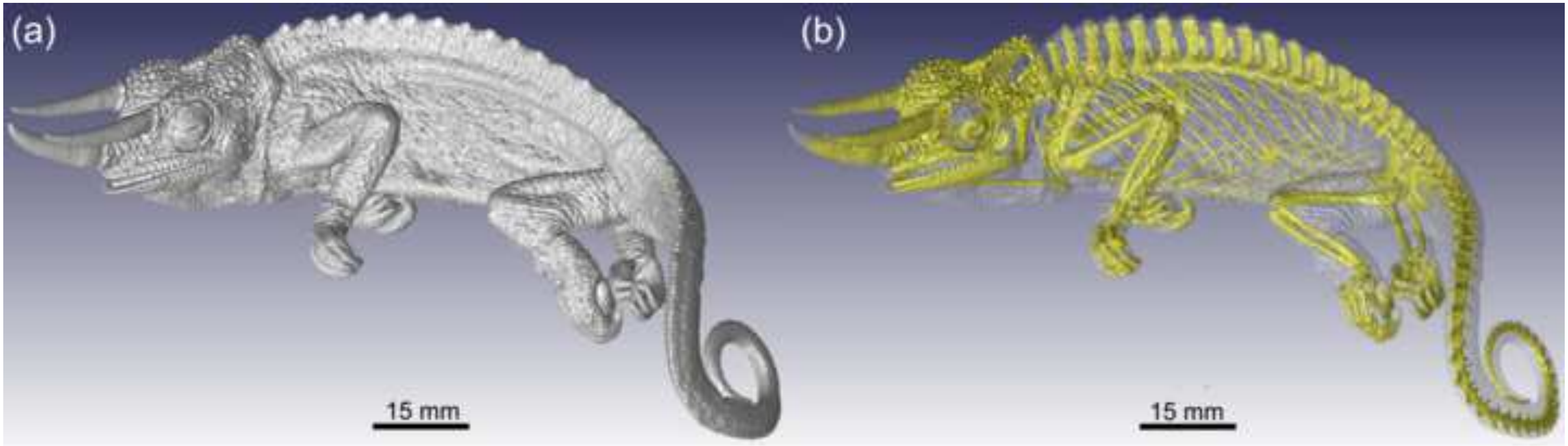

-

 Click here to download Figure Figure5.tif

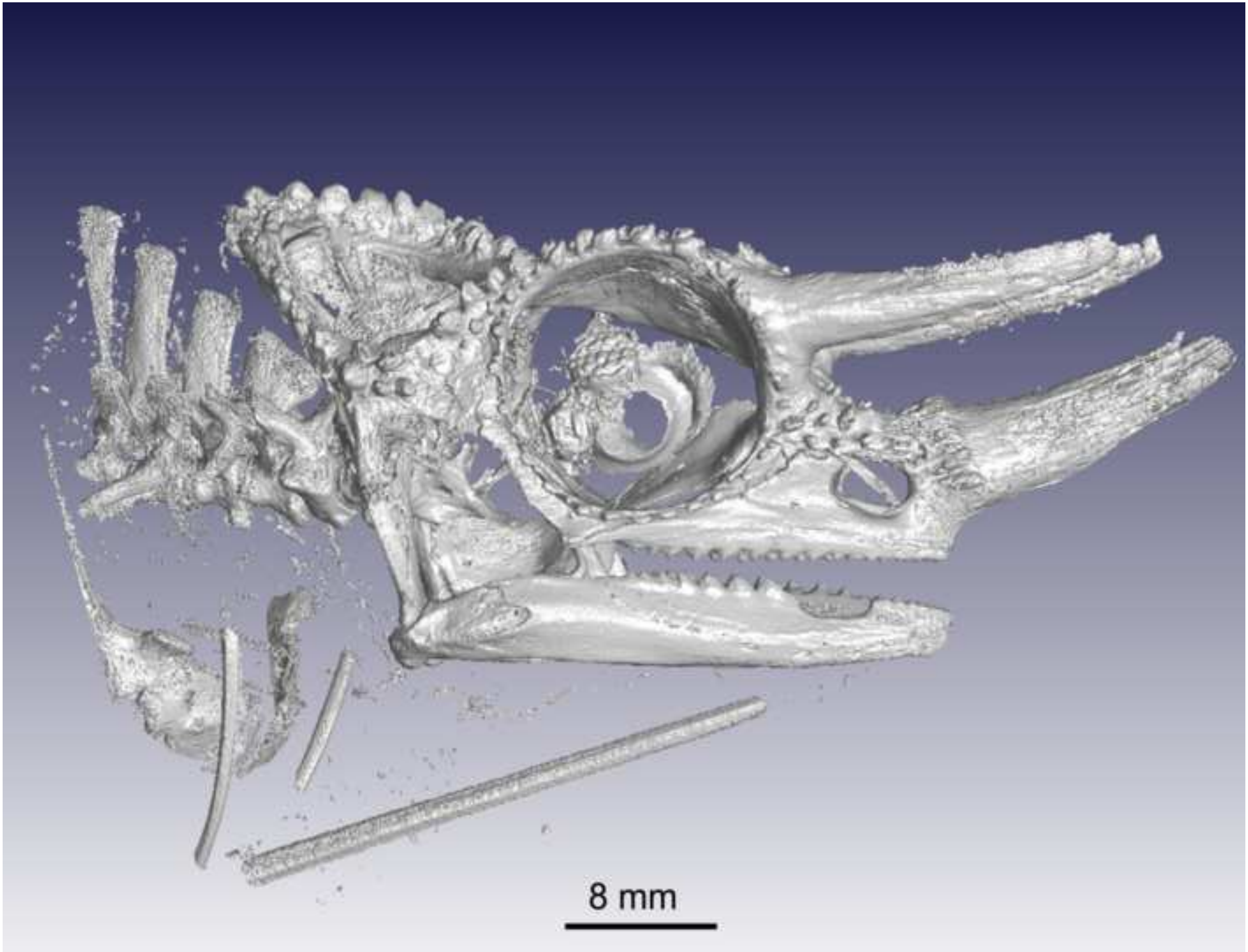

Click here to download Figure Figure6.tif

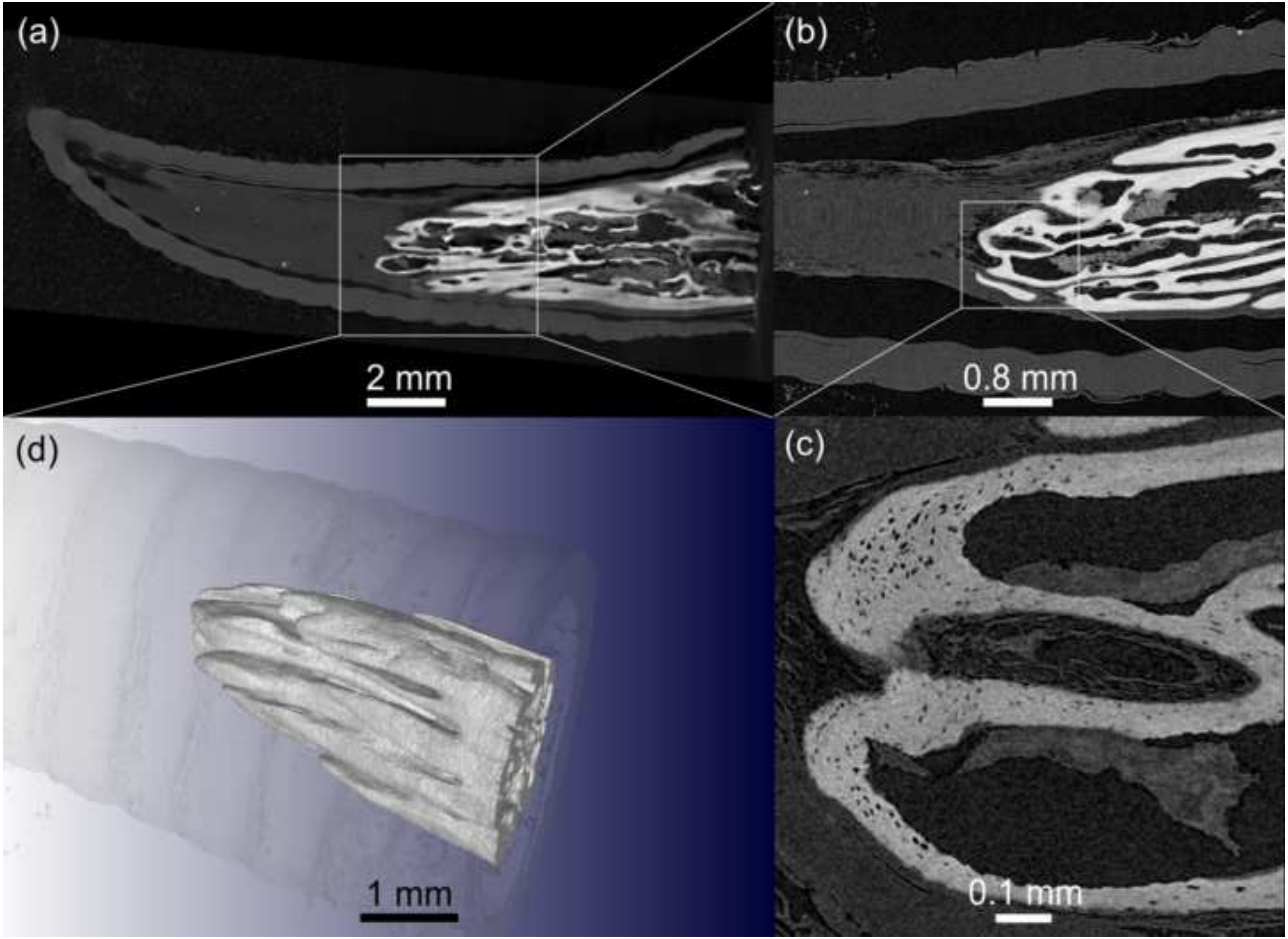

Dear Dr. Scott Edmunds,

Re: Resubmission of reviewed manuscript (GIGA-D-16-00031.R1) for GigaScience

Thank you for considering resubmission of the manuscript that we submitted to GigaScience. We have read carefully through the comments of the two reviewers. All make excellent suggestions to improve the study, which we address below, followed by a response to more specific comments.

As suggested by the reviewers, we have included a zoologist (Dr. Chris Broeckhoven) who has revised the manuscript. The following major revision steps have been taken:

1) **Word count**: we agree that the word count of the paper (i.e. ± 9800 words) was too high and a reduction to ± 7000 words might be more than acceptable. For this reason, and after some consideration, we have decided to delete several paragraphs and sections from the current manuscript. We believe that these sections do not contribute to the strength of the manuscript, or are not required for understanding the process of micro-CT scanning. Likewise, we believe that the image analysis section is redundant in the current version of the manuscript. Image analysis highly depends on the software that is deployed by the user (in which case user manuals would be more suitable to consult).
2) **Grammar and wording**: We have done considerable effort to edit the grammar and wording of the manuscript. Many sentences (which aren't specified here) have been rephrased, checked for spelling mistakes and linguistic errors. In addition, sections were rephrased to improve flow and a less casual writing style was adopted.
3) **Structure**: Several changes have been made to improve the structure of the manuscript. In particular, the section on scanning errors and artefacts has been moved to the end as this section requires an understanding of the various steps of scanning (preparation, reconstruction etc), which might confuse the reader if it is mentioned too much in advance.
4) **Figures**: We have edited all the figures, which we believe are now more suitable for publication. In addition, we have replaced Figure 1 with an image of the actual scanner. It will be easier for users to familiarize themselves with an actual micro-CT set-up compared to a schematic drawing.

We address some specific comments to issues raised by the reviewers below, but marked them "N/A" if the issue concerns a section or sentence that is no longer part of the current version of the manuscript.

Reviewer 1

Page 2, line 1: "The ability to perform noninvasive analysis is often of prime concern when working with biological samples." Overly generalized statement, a cell biologist is working with biological samples and will readily use histology or TEM (invasive!) to conduct his analyses
COMMENT: First sentence has been removed and instead emphasis was given to the second sentence.

Page 2, line 17: three dimensional written in text form, although the abbreviation (3D) has been introduced a few lines before.
COMMENT: Care was taken to use the abbreviation (3D) in the remainder of the manuscript after its initial introduction.

Page 2, line 27: I am not aware of the term "μ (XCT)"
COMMENT: N/A

Page 2, line 38: "...from small low-cost benchtop systems to cabinet systems able to house larger samples and even as large as walk-in cabinet systems..." this sentence is not correct English in my opinion.
COMMENT: N/A

Reviewer 2

Section 3.1.1, first sentences: In the response to the reviewers, a different new first paragraph is cited (" Section 3.1.1 has been changed accordingly and now reads: "However, some soft tissue samples are preserved in a liquid and will damage if removed, therefore requiring scanning in the liquid as is. In these cases, staining increases the contrast of the specimen compared to the surrounding medium [4, 12, 11].""). There seems to be some confusion, please double check the text for the most up-to-date version.
COMMENT: The entire section on sample mounting has been rephrased and comments have been incorporated.

Section 3.1.1, p. 5, lines 10 and following: The issue with scanning samples in liquid (ethanol) is not their damage through manual handling but the potential desiccation when removed from their storage liquid, which causes irreversible changes to the morphology. Please consult with an invertebrate zoologist who is used to handle specimens preserved in liquids and correct this section. In addition, the sentence "It is also possible to scan samples in liquid filled..." does now not fit with the previous sections.
COMMENT: see above

Section 3.1.1, p. 5, lines 23 and following "The vertical mount method" is not explained.
COMMENT: N/A

Page 8. line 20: figure 3 is referred to in the text before any reference to Fig2. Check if this is OK with the journal.
COMMENT: Figure order has been changed

Page 8. line 34: Although the reference to 3.1.6 about beam hardening has been inserted, the actual explanation on beam hardening has not been moved from the (old) section 3.1.5 to 3.1.6, thus there is currently no explanation on beam hardening in the ms.
COMMENT: Beam hardening is now explained in Section 3.2.4.

Page 8. line 35: Sentence "This penetration value" should go further up in the paragraph (somewhere after the first sentence) to ensure a better text flow.
COMMENT: Placement of "penetration value" has been changed to improve text flow.

Page 8. line 38: Verbose sentence/paragraph, shorten.
COMMENT: N/A

Page 9, guidelines: These are actually not guidelines, they don't stand alone (e.g. III makes no sense without II), Please merge them and rephrase them so that they can be used as independent guidelines. In addition, II and V are similar, can be merged)
COMMENT: The number of guidelines have been reduced. Subsections of guideline III have been edited and merged.

Page 12, lines 34 and following: A reference to "some scanners" has been inserted in the sentence, but now it reads as if some scanners have the option to remove the bright ring, not that the bright ring is only present in some scanners.
COMMENT: N/A

Page 13, first paragraph: Can be shortened to make more clear and understandable. Rephrase to make clear that microCT does not have a built-in calibration, but data can be calibrated. Currently, the difference between medical scanners and microCT are unclear due to the style of writing.
COMMENT: N/A

Page 14 line 2: replace "notepad" with "text editor" (notepad is a commercial product by Microsoft, not a general term for the type of software)
COMMENT: N/A

Section 3.4. Please rewrite the whole paragraph until line 25. The differences between surface and volume rendering are still not clear, probably contain errors (Volume rendering does not involve isosurface views), and remove the reference to CAD which is likely unknown to users. In addition, Blender is listed twice in the software section.
COMMENT: N/A

Section 3.5. The different options of thresholding are very confusing to read and difficult to understand. If possible, rephrase, in logical order, with clear explanations. If this is impossible without visual examples, please remove parts of the section. Currently, it is not helpful to a novel user.
COMMENT: N/A

Page 15, line 12: Filtering the data is mentioned, but the explanation to data filtering is given below this paragraph. Add a reference (e.g. "see below") to make it more easy to understand.
COMMENT: N/A

Page 15, line 33 and following: Paragraph is still unclear. It looks as if smoothing should be done before the segmenting (which you describe above)? Thus, move it before those steps in the description. Jumping back and forth between steps is confusing. Binarization is still unclear, too. why is it done here?
COMMENT: N/A

Summary: Reference to the ostecyte structure is given, but not mentioned anymore in the text.
COMMENT: We decided not to go into detail on the cell type itself but rephrased it as “bone micro-architecture”
