# Supplementary material for: Laboratory x-ray micro-computed tomography: a user guideline for biological samples
Source: Gigascience. 2017 Apr 17;6(6):1–11. doi: 10.1093/gigascience/gix027 (PMC5449646; doi:10.1093/gigascience/gix027)
Supplement: Response_to_reviewer_comments_Orginal_Submission.pdf [file gix027_Response_to_reviewer_comments_Orginal_Submission.pdf]

Dear Dr Edmunds

Re: Resubmission of reviewed manuscript (GIGA-D-16-00031) for GigaScience

We hereby re-submit our reviewed manuscript titled: Laboratory X-ray micro-computed tomography: a user guideline for biological samples (previous title: Laboratory X-ray micro-computed tomography: a generalised approach for biological samples using a three-horned chameleon as example). In this new submission the points raised by the reviewers have been addressed and, once again, the reviewers are thanked for their thorough and valuable contributions towards improving this manuscript.

The changes made to this manuscript are described point-by-point (in red) in response to reviewer comments in the personal cover letter at the back of the file.
