# Supplementary material for: Laboratory x-ray micro-computed tomography: a user guideline for biological samples
Source: Gigascience. 2017 Apr 17;6(6):1–11. doi: 10.1093/gigascience/gix027 (PMC5449646; doi:10.1093/gigascience/gix027)
Supplement: Response_to_reviewer_comments_Revision_1.pdf [file gix027_Response_to_reviewer_comments_Revision_1.pdf]

Dear Dr. Scott Edmunds,

Re: Resubmission of reviewed manuscript (GIGA-D-16-00031.R1) for GigaScience

Thank you for considering resubmission of the manuscript that we submitted to GigaScience. We have read carefully through the comments of the two reviewers. All make excellent suggestions to improve the study, which we address below, followed by a response to more specific comments.
As suggested by the reviewers, we have included a zoologist (Dr. Chris Broeckhoven) who has revised the manuscript. The following major revision steps have been taken:
1) Word count: we agree that the word count of the paper (i.e. ± 9800 words) was too high and a reduction to ± 7000 words might be more than acceptable. For this reason, and after some consideration, we have decided to delete several paragraphs and sections from the current manuscript. We believe that these sections do not contribute to the strength of the manuscript, or are not required for understanding the process of micro-CT scanning. Likewise, we believe that the image analysis section is redundant in the current version of the manuscript. Image analysis highly depends on the software that is deployed by the user (in which case user manuals would be more suitable to consult).
2) Grammar and wording: We have done considerable effort to edit the grammar and wording of the manuscript. Many sentences (which aren't specified here) have been rephrased, checked for spelling mistakes and linguistic errors. In addition, sections were rephrased to improve flow and a less casual writing style was adopted.
3) Structure: Several changes have been made to improve the structure of the manuscript. In particular, the section on scanning errors and artefacts has been moved to the end as this section requires an understanding of the various steps of scanning (preparation, reconstruction etc), which might confuse the reader if it is mentioned too much in advance.
4) Figures: We have edited all the figures, which we believe are now more suitable for publication. In addition, we have replaced Figure 1 with an image of the actual scanner. It will be easier for users to familiarize themselves with an actual micro-CT set-up compared to a schematic drawing.

We address some specific comments to issues raised by the reviewers below, but marked them "N/A" if the issue concerns a section or sentence that is no longer part of the current version of the manuscript.

Reviewer 1
Page 2, line 1: "The ability to perform noninvasive analysis is often of prime concern when working with biological samples." Overly generalized statement, a cell biologist is working with biological samples and will readily use histology or TEM (invasive!) to conduct his analyses
COMMENT: First sentence has been removed and instead emphasis was given to the second sentence.

Page 2, line 17: three dimensional written in text form, although the abbreviation (3D) has been introduced a few lines before.
COMMENT: Care was taken to use the abbreviation (3D) in the remainder of the manuscript after its initial introduction.

Page 2, line 27: I am not aware of the term "µ (XCT)"
COMMENT: N/A

Page 2, line 38: "...from small low-cost benchtop systems to cabinet systems able to house larger samples and even as large as walk-in cabinet systems..." this sentence is not correct English in my opinion.
COMMENT: N/A

Reviewer 2
Section 3.1.1, first sentences: In the response to the reviewers, a different new first paragraph is cited (" Section 3.1.1 has been changed accordingly and now reads: "However, some soft tissue samples are preserved in a liquid and will damage if removed, therefore requiring scanning in the liquid as is. In these cases, staining increases the contrast of the specimen compared to the surrounding medium [4, 12, 11]."").

There seems to be some confusion, please double check the text for the most up-to-date version.
COMMENT: The entire section on sample mounting has been rephrased and comments have been incorporated.

Section 3.1.1, p. 5, lines 10 and following: The issue with scanning samples in liquid (ethanol) is not their damage through manual handling but the potential desiccation when removed from their storage liquid, which causes irreversible changes to the morphology. Please consult with an invertebrate zoologist who is used to handle specimens preserved in liquids and correct this section. In addition, the sentence "It is also possible to scan samples in liquid filled..." does now not fit with the previous sections.
COMMENT: see above

Section 3.1.1, p. 5, lines 23 and following "The vertical mount method" is not explained.
COMMENT: N/A

Page 8. line 20: figure 3 is referred to in the text before any reference to Fig2. Check if this is OK with the journal.
COMMENT: Figure order has been changed

Page 8. line 34: Although the reference to 3.1.6 about beam hardening has been inserted, the actual explanation on beam hardening has not been moved from the (old) section 3.1.5 to 3.1.6, thus there is currently no explanation on beam hardening in the ms.
COMMENT: Beam hardening is now explained in Section 3.2.4.

Page 8. line 35: Sentence "This penetration value" should go further up in the paragraph (somewhere after the first sentence) to ensure a better text flow.
COMMENT: Placement of “penetration value” has been changed to improve text flow.

Page 8. line 38: Verbose sentence/paragraph, shorten.
COMMENT: N/A

Page 9, guidelines: These are actually not guidelines, they don't stand alone (e.g. III makes no sense without II), Please merge them and rephrase them so that they can be used as independent guidelines. In addition, II and V are similar, can be merged)
COMMENT: The number of guidelines have been reduced. Subsections of guideline III have been edited and merged.

Page 12, lines 34 and following: A reference to "some scanners" has been inserted in the sentence, but now it reads as if some scanners have the option to remove the bright ring, not that the bright ring is only present in some scanners.
COMMENT: N/A

Page 13, first paragraph: Can be shortened to make more clear and understandable. Rephrase to make clear that microCT does not have a built-in calibration, but data can be calibrated. Currently, the difference between medical scanners and microCT are unclear due to the style of writing.
COMMENT: N/A

Page 14 line 2: replace "notepad" with "text editor" (notepad is a commercial product by Microsoft, not a general term for the type of software)
COMMENT: N/A

Section 3.4. Please rewrite the whole paragraph until line 25. The differences between surface and volume rendering are still not clear, probably contain errors (Volume rendering does not involve isosurface views), and remove the reference to CAD which is likely unknown to users. In addition, Blender is listed twice in the software section.

COMMENT: N/A

Section 3.5. The different options of thresholding are very confusing to read and difficult to understand. If possible, rephrase, in logical order, with clear explanations. If this is impossible without visual examples, please remove parts of the section. Currently, it is not helpful to a novel user.
COMMENT: N/A

Page 15, line 12: Filtering the data is mentioned, but the explanation to data filtering is given below this paragraph. Add a reference (e.g. "see below") to make it more easy to understand.
COMMENT: N/A

Page 15, line 33 and following: Paragraph is still unclear. It looks as if smoothing should be done before the segmenting (which you describe above)? Thus, move it before those steps in the description. Jumping back and forth between steps is confusing. Binarization is still unclear, too. why is it done here?
COMMENT: N/A

Summary: Reference to the ostecyte structure is given, but not mentioned anymore in the text.
COMMENT: We decided not to go into detail on the cell type itself but rephrased it as “bone micro-architecture”
