# Supplementary material for: Laboratory x-ray micro-computed tomography: a user guideline for biological samples
Source: Gigascience. 2017 Apr 17;6(6):1–11. doi: 10.1093/gigascience/gix027 (PMC5449646; doi:10.1093/gigascience/gix027)
Supplement: Response_to_reviewer_comments_Revison_2.pdf [file gix027_Response_to_reviewer_comments_Revison_2.pdf]

Copy edited version uploaded here, addressing points requested by the referees, and also improvements to readability by a more general audience.
