# Supplementary material for: Laboratory x-ray micro-computed tomography: a user guideline for biological samples
Source: Gigascience. 2017 Apr 17;6(6):1–11. doi: 10.1093/gigascience/gix027 (PMC5449646; doi:10.1093/gigascience/gix027)
Supplement: Reviewer_1_Report_(Revision_1).pdf [file gix027_Reviewer_1_Report_(Revision_1).pdf]

Reviewer Report

Title: "Laboratory X-ray micro-computed tomography: a user guideline for biological samples"

Version: Revision 1 **Date:** 9/5/2016

Reviewer name: Alexander Ziegler

Reviewer Comments to Author:

SG le Roux now more comprehensively and concisely provides the reader with an introduction to µCT scanning. Although the article constitutes a significant improvement compared to previous versions, I still recommend that the authors undertake a MAJOR REVISION of their manuscript.

One of the primary reasons for this recommendation is that in my view the article still is not properly written for the intended audience, i.e. newcomers to µCT from a primarily biological background. While some parts of the article can be easily understood, other aspects can only be grasped with a considerable degree of experience in µCT scanning. I would therefore like to encourage the authors once more to seek the help (maybe even in the form of a co-authorship) of a zoologist without any knowledge in µCT. This person will certainly rapidly be able to identify those parts of the manuscript that are currently unsuitable for a reader without any knowledge in this technique.

Another point that still requires attention as well as love for detail are grammar, style, and wording of the text - although English is not my mother-tongue, I can easily spot numerous mistakes, sometimes several on a single page. These mistakes convey the impression that the authors did not take their submission fully seriously. This same notion also applies to the figures (see my comments below). Here just a few examples from the first text page:

- Page 2, line 1: "The ability to perform non-invasive analysis is often of prime concern when working with biological samples." - overly generalized statement, a cell biologist is working with biological samples and will readily use histology or TEM (invasive!) to conduct his analyses

- Page 2, line 17: three dimensional - written in text form, although the abbreviation (3D) has been introduced a few lines before

- Page 2, line 27: I am not aware of the term "µ (XCT)"

- Page 2, line 38: "...from small low-cost benchtop systems to cabinet systems able to house larger samples and even as large as walk-in cabinet systems..." - this sentence is not correct English in my opinion

These aspects (grammar, style, content) as well as the fact that the text should be thoroughly checked for consistency once more lead me to suggest a MAJOR REVISION. I would like to urge the authors to invest more time in the preparation of their manuscript.

Figures

As in my previous comments I would like to encourage the authors to adhere to the basic guidelines in the preparation of figures for zoomorphological publications. For example, in case of figure plates, it is custom to align the individual images in size - this basic concept is not followed in Figure 2 (unprecise placing of images), Figure 4 (missing fourth image), and Figure 6 (quite chaotic figure). Furthermore, in the schematic Figure 1 the position of the detector is confusing (facing the reader instead of the radiation source), while in Figure 3 scales are missing. In addition, the authors need to postition the images in Figure 6 in a standardized orientation - it is presently quite difficult to identify the areas that are shown at the consecutive higher magnifications.

Kind regards

Alexander Ziegler

Level of Interest

Please indicate how interesting you found the manuscript: An article whose findings are important to those with closely related research interests

Quality of Written English

Please indicate the quality of language in the manuscript: Needs some language corrections before being published

Declaration of Competing Interests

Please complete a declaration of competing interests, considering the following questions:

- Have you in the past five years received reimbursements, fees, funding, or salary from an organisation that may in any way gain or lose financially from the publication of this manuscript, either now or in the future?

- Do you hold any stocks or shares in an organisation that may in any way gain or lose financially from the publication of this manuscript, either now or in the future?
- Do you hold or are you currently applying for any patents relating to the content of the manuscript?
- Have you received reimbursements, fees, funding, or salary from an organization that holds or has applied for patents relating to the content of the manuscript?
- Do you have any other financial competing interests?
- Do you have any non-financial competing interests in relation to this paper?

If you can answer no to all of the above, write 'I declare that I have no competing interests' below. If your reply is yes to any, please give details below.

Click here to enter text.

I agree to the open peer review policy of the journal. I understand that my name will be included on my report to the authors and, if the manuscript is accepted for publication, my named report including any attachments I upload will be posted on the website along with the authors' responses. I agree for my report to be made available under an Open Access Creative Commons CC-BY license (http://creativecommons.org/licenses/by/4.0/). I understand that any comments which I do not wish to be included in my named report can be included as confidential comments to the editors, which will not be published.

I agree to the open peer review policy of the journal
