# Supplementary material for: Laboratory x-ray micro-computed tomography: a user guideline for biological samples
Source: Gigascience. 2017 Apr 17;6(6):1–11. doi: 10.1093/gigascience/gix027 (PMC5449646; doi:10.1093/gigascience/gix027)
Supplement: Reviewer_2_Report_(Original_Submission).pdf [file gix027_Reviewer_2_Report_(Original_Submission).pdf]

Reviewer Report

Title: "Laboratory X-ray micro-computed tomography: a user guideline for biological samples"

Version: Sarah Faulwetter **Date:** Click here to enter a date. July 7, 2016

Reviewer name: Sarah Faulwetter

Reviewer Comments to Author:

The re-submitted version of the paper has been improved substantially compared to its previous version and will become a valuable document for micro-CT users. However, I believe it still has room for some improvements, and I am attaching my suggestions below. As the document contains many technical aspects and descriptions of working practices that invariably are influenced by the authors' experiences and equipment, I will try to point out those points that can be generalized even more, to be applicable to other samples, instruments, types of studies, etc.

I recommend publication of the manuscript after thorough revision.

General comments:

- In parts, the language and style of the ms is rather narrative and could be more parsimonous and concise, containing many empty phrases such as "it is important to note", "generally", "more generally", etc. which can often be omitted. In addition, phrases such as "being a fascinating test sample", "the mystery that surrounds the scanning process" are, in my opinion, not an appropriate scientific writing style and sound rather strange. I may refer to text sections that could be improved in style in my more specific comments below, but as I won't be able to mention them all, the paper may benefit from an overall linguistic editing to improve the style. Several small grammatical and typographical errors will also need to be seen to.

- A number of important publications in the field of biological micro-CT imaging are not mentioned in the ms and should be included, as they provide very valuable further reading for users new to micro-CT. Many of the points explained by the authors have been described in slightly different ways (or with additional information) by other authors, and this publication would benefit enormously from a comprehensive literature overview as it would then serve as a good starting point for new micro-CT users. A few missing literature references will be listed below in the specific comment section, but only

those where it was immediately obvious that certain references are missing. More thorough work is required by the authors.

- In parts, the manuscript still mentions complex physical/engineering terms and concepts without further explanation. Being an experienced micro-CT user myself, but with a biology background, I still had difficulties understanding several more technical text sections. The text should guide the user through these more technical parts carefully. My advice here would be to ask a colleague with little or no expertise in micro-CT to go through the text and identify those difficult sections or terms.

- Much weight is given to the example (the three-horned chameleon) in several sections (title, abstract, keywords, discussion of the example). In my opinion, while it is useful to have an example, it is just this - an example for imaging to illustrate the actual purpose of the paper, and could have been done with any object. The aim of the paper is not to conduct a biologically meaningful analysis of a specific organism, so the (understandable) enthusiasm over the object which can be discerned throughout the text should be reduced (see suggestions in specific comments) to focus on the actual aim of the paper

- Use one of the available terms (e.g. micro-CT, X-ray micro-CT, laboratory X-ray CT) throughout the text.

Specific comments:

Title:

- I would remove the part "using a three-horned chameleon as an example" from the title for reasons outlined above

- The previous version had the phrase "user guide" in the title - which I find more useful and catchy as "a generalized approach". This is a kind of user manual, after all, and mentioning it in the title will attract more readers.

Abstract:

- The abstract is rather narrative and should be more concise and avoid phrases like "fascinating", "It is important to note". As this is a user guide, a more detailed description of the contents could be given, e.g. mention that an introduction is given, scanning setup, reconstruction and visualization options are presented and their background explained, etc etc, and that finally an example is given. This example should not be mentioned in more than one sentence - it is an example to explain / guide the user through a scan and in the context of this paper does not convey any biological meaning. Overall, the abstract will need to be re-written to reflect the contents of the paper more precisely.

Keywords:

- Industrial CT is probably not a keyword many users will use, especially not biologists (or those focusing on scanning biological samples). Consider replacing it or removing it.

- Remove "three-horned chameleon" or replace with the scientific name.

- "non-destructive testing" should be "non-destructive imaging" or "non-destructive analysis"? Although the term exists, it refers to mechanical / engineering applications. Users with a biology background would much more likely search for "imaging" or "analysis" than "testing".

- consider adding "user guide" or something similar to the keywords, unless you decide to include it in the title.

1. Introduction:

- Line 28: "...has been given to find techniques..." -> "...has been given to finding techniques..."

- Lines 32- 36 basically say the same thing twice - can be shortened.

- Line 44: I agree with the definition of nano-CT for imaging at sub-micron resolution. This is repeated (in part) in the paragraph lines 202-207, however, later in the paper (lines 730 and following) you refer to resolutions around 5-10µm as nano-CT (or being scanned with nano-CT / overlap between nano-CT and micro-CT). This may be specific to your scanner models with are termed as such? I am familiar with scanners that routinely scan at 1-10µm resolution and are termed micro-CT.). This should be clarified -

maybe just by explaining (possibly in the paragraph lines 202-207) that terms are not as fixed as they may seem and that they may depend on the specific model of the scanner?

- Line 47 and following: "potentially much improved and variable" - sounds strange, rephrase. Also, it does not become clear how the revolving sources vs. the revolving sample influence resolution. I suggest removing it, or rephrasing. The revolving source/sample could also be mentioned as another difference between systems.

- Line 50: "dose-limited" and "beam filtration" are not easily understood for users not familiar with the topic. Maybe omit or rephrase keep more general here (don't go into detail though).

- Line 54 and following: the explanation of the differences between synchotron and lab-Xray are not helpful for the reader - it would be more useful to explain that the one produces monoenergetic x-rays, whereas common targets (such as tungsten) in micro-CT instruments typically produce a broad spectrum of x-ray energies, as this is the basis for the use of different energies for different samples and the need for filters. The term "X-ray brightness" is not understandable to a non-expert here.

- Lines 64 and following: Here you could add a few more references to important or exemplar papers, especially to the field of biology, as the mentioned review is from 2012. As far as I know, no newer review is available for all biology, but some comprehensive papers have been published in both zoology and botany using micro-CT.

- Lines 68 and following. Remove/replace "mystery" and "de-mystifying" (non-scientific language). In addition, I think that the "mystery" of micro-CT is not one of the main reasons for underutilization, it's the lack of access to facilities and /or lack of funding (since often micro-CT services are costly, especially since biodiversity researchers/taxonomists) do often not have funds available to either buy and maintain a scanner or outsource the analyses. You may want to re-consider the first sentence of the paragraph. In addition, if you mention staining, you should cite Brian Metscher's works as these are considered the most comprehensive and very useful papers on staining biological samples, and there is a comprehensive work on staining by Pauwels et al (doi: 10.1111/jmi.12013). Similar "how to" guides exist also for plant studies (e.g. Staedler et al 10.1371/journal.pone.0075295).

2. Background to CT:

- Line 92: "The fast moving electrons hitting a metal target material creates X-rays" → "...create X-rays"

- Line 93: "the resulting cone beam" - for a non-expert, this may need explanation. Either remove the term or explain it (either here or in conjunction with explaining edge artefacts / cone beam artefacts or with why to choose only central part of detector).

- Line 105: "...with discussion" → ".. with a discussion"

3. Computed tomography basics:

- Line 135: Please cite here also other works on staining, e.g. Metscher, Pauwels, others - for many biological samples staining is crucial to obtain satisfactory results, and there is excellent literature on this.

- Lines 136 and following: The common reason for staining is to be able to scan it in a liquid, as many soft-tissue samples in a liquid preservation medium cannot be dried, as this would destroy the sample and distort their morphology. Chemical, freeze drying or critical point drying may be used for those specimens in some cases, but may make the specimen brittle and fragile, again risking damage during handling. In these cases, staining increases the contrast of the specimen compared to the surrounding medium and the scanning container, so even if the specimen is held in place by the walls of the container (see line 156), both liquid and container can usually be rendered transparent in the final visualisation. Please remove - or better - explain the drying step from your explanations and explain the reasons for staining in more detail. I also think that the statement that staining should only be used if "all other options have failed" is misleading. There are specific reasons for staining, and the ideal contrast method - staining or drying or none - depends on a variety of factors, including the characteristics of the specimen and the scope of the study, thus staining is not a "last resort" method.

- Lines: 193/194: Explain the reason behind artefacts from the edges.

-Lines 202-207: The lower limit of 500nm for the nano CT may be instrument-specific to the phoenix nanotome. Several other nano-CT models (Xradia, SkyScan) have a higher resolution (around 200-50nm), and those maximal resolutions should be mentioned, as they may be of interest for peopole. In this context, it would be also beneficial to explain the differences between voxel size and resolution (e.g. looking at specifications of instruments on websites you may find both information on voxel/pixel size as well as on resolution). For a new user, or someone who needs to decide which instrument to buy, this is important information. The information in paragraph 3.1.3 is not too helpful in this context.

- Line 216: "This value does not consider the actual spatial resolution capability..." and "X-ray spot size" Explain what "spatial resolution capability" and "X-ray spot size" mean and how they are related, so that a user can judge (e.g. when buying an instrument, or when deciding whether certain small features in the sample will be displayed satisfactorily) whether the instrument's capabilities are suitable. These are complex facts and difficult to explain - maybe an example or a small graph may help? This paragraph is important, and good explanation are rare in literature, so should be explained well and will be of help to many users.

- Line 238: " have typical image acquisition times" → " may have image acquisition times from a few hundred ms to up to several seconds" (the exposure time really varies a lot depending on scanner model, voltage and filter used, so I think raw numbers are not helpful here.).

- Line 248: "Averaging reduces noise" → " This averaging reduces nose" (term "averaging" has not been mentioned as such to explain the previous sentence, so add "this" just for clarity)

- Line 262: "on the detector in width multiplied" → add a unit to width (pixels, I guess)

- Line 262 - Guideline: I have seen the formula N = width * π (thus, 3.14, not 1.6) to calculate the number of projection images. However, I am not familiar with the background to this, so I don't know whether one or the other can be used under certain circumstances (e.g. full vs. half rotation). Please just double-check that this is correct.

- Line 263: add comma after Consequently

- Line 270: add comma after In fact

- Line 283: Remove the explanation about beam hardening here (line 283 until end of page) and just refer to the section on artefacts (i.e. ".... causing noise and artefacts (see section 3.1.6 for detailed explanations)")

- Line 290 and following: very narrative, verbose style. Rephrase or remove (remove parts on beam hardening certainly, as these should be treated in the section on artefacts).

- Line 208: weird phrasing "the smaller the sample the lower the voltage that is possible". Rephrase to be more concise

- Line 312 and following: Explain which filters are used for what - just explaining that aluminium is a "popular choice" is not very helpful.

- Line 315: "relevant to very dense objects and not biological samples" → biological samples can be dense, too (bones, calcified tissues) and may require up to 100kV, where a filter should - at least in certain scanners - certainly be used! Please remove / rephrase the sentence or put it into context - at the moment it sounds as if filters are not needed for biological samples.

- Line 316: "Detector filtering": you did not mention /explain the opposite (beam filtering), it is only assumed to be what you describe in general. Maybe start the paragraph with describing the general two

setups of filters: either filter between source and object, or between object and detector (and use the correct terms for each, as you refer to them later again) and what are the differences between them, and the effects each may create. "Secondary X-ray emission" and "scattering" are not explained here and not understandable to new users, so please try to explain better.

- Line 324: "Calculate the sample's minimum penetration..." → Explain how this is done: automatically by the machine? by the user? Should the user refer to the manual to check how the scanner model does this? As the sentence stands, a new user will not understand what needs to be done and thus this advice is not helpful.

- Guidelines IV: You mention minimum 10% explicitly in ii, then repeat in iv again the limits - maybe follow same style: if minimum is below 10%, raise voltage, if above 90%, lower voltage. Otherwise there is partial overlap.

- Lines 345 and following: The part on hardware misalignment is not well explained here - why does this lead to the specific artefact?

- Paragraph on artefacts: There are publications on CT artefacts, you may want to cite them (e..g. Barret and Keat, "Artifacts in CT: Recognition and Avoidance", or Boas and Fleischmann, "CT artifacts: causes and reduction techniques").

- Line 366: Beam centering is not a (manual) option in every scanner model (or maybe named differently). Rephrase accordingly, or refer user to manual, or mention that this may be done automatically.

- Lines 375 and following: It may be helpful to explain how these fast scans can be achieved. Which parameters should be changed to achieve a faster scan?

- Lines 394 and following: Please remove mention of scanner-specific built-in software (not useful for users with another scanner) and provide URLs for the independent software so users can obtain additional information

- Line 405: "A choice of data type can be made" → "A choice of data type for the output"

- Lines 407 and following: This option is not really clear to me. Could you rephrase?

- Lines 428 and following: Option is not available in all scanners, rephrase to "it may also be possible" or something similar. Please explain scattering (if you have not done so before in the text, in the revised version). Add comma after "scattering is present".

- Lines 436: I have never seen this "bright ring", so it may be scanner-dependent. Please rephrase accordingly

- Exchange order of last two paragraphs in section 3.3.1 ("In medical CT..." and "It is also possible...").

- Add to the calibration section that some scanners allow calibration to HU units by using calibrated specimens.

- Line 455: Using 180° scans is not a "seldom used option". In fact, I know labs where 180° is the common setting, and 360° is only used if extra detail is needed or if the sample is dense. You may want to explain in which cases either option can be useful.

- Lines 456: Example for a redundant phrase "it can be mentioned here that in the case of data collection, sometimes an image could get corrupted" → make more parsimonous: "During data collection, images may become corrupted...". Please check the ms for such styles and rephrase them, as they are tiresome to read and blow up the text unnecessarily.

- Line 461: Table 1 may be better placed after the artifacts section, not after the reconstruction section.

- Line 485: maybe add another example besides IrfanView, and give URLs

- Lines 487 and following: First few sentences are unclear to me. I believe they are meant to explain the section on scalebars, but they seem a little confusing to me. Maybe rephrase to express the main point more clearly?

- Line 498 and following: Start by explaining that CT data can be 3D- visualized in two different ways: volume rendering and surface rendering, explain the one and then the other, not by stating what volume rendering is *not*, introducing a term (CAD data) which the user is not likely to know either. In the list of softwares there are some double mentions. Add Amira, which is a very commonly used software. Blender is open source, too. You may want to differentiate between freeware and commercial, not necessarily open source (users may be more interested in the costs). Give URLs for all packages.

- Section 3.5. needs a general restructuring. It jumps back and forth between concepts and does not follow a clear line of thought. More specifically some points below:

- Line 511 and following: It does not become clear towards what aim thresholding should be used. What is "image processing" in this context? Segmentation? Analysis? Visualization? To me, the paragraph reads more like a guide to applying transfer function to a volume-rendered object. In this case, the information should go under the previous paragraph "visualisation".

- Line 516: Replace "animal" with "object"/"sample"(also further down in paragraph)

- Line 516: Sentence "Aligning the slice image to the feature allows ..." is not understandable.

- Line 517: "Its selection was done..": What is "it" and use past tense? Are you referring to a specific example here? Rephrase or remove the whole following paragraph, looks as if it is misplaced, referring to "here", "animal", …

- Line 518: Region growing should be better placed under the segmentation options

- Line 527: "During image processing": image processing is a very general term. Which step are you referring to here? Replace "are initially used" to "can be used".

- Line 232: What are "the respective voxels"?

- Line 533: What is the binarization step here? Thresholding and segmentation is always binarization, creating selected and non-selected regions.

- Line 535: "Thresholding can be done in different ways" → merge with paragraphs above.

- Line 541: replace "colouring" with "selecting"

- Line 545: "significantly filter the data" is unclear

- Lines 546/547: double use of "required" - rephrase sentence.

- Line 549: "Image analysis aims to achieve the extraction ..." → sounds strange, rephrase

- Lines 577 and following: "The fact that the same sample" → why should that be confusing to a user? It's a simple matter of choosing an adequate "zoom level", as in photography, not a strange concept. I suggest removing it.

- Lines 581/ 583: Either 40 minutes or 1 hour. Confusing otherwise.

- Lines 584 and following: The aim here is not to achieve a longer or shorter scan, but to scan at a different resolution, which, in turn, has an impact on the scan time.

- I suggest rephrasing the whole paragraph lines 574 to 593 to make it more concise - at the moment it is confusing to read. Maybe a small table could be more helpful in summarizing the impacts of resolution on scan time and quality of image. Remove empty and redundant phrases from this paragraph.

- Line 593: "next section" - refer to specific section, since they are numbered

- Lines 595 and following: does not really fit here - it has to do with resolution, but the transition is a little abrupt. Explain "correlation" of SEM and micro-CT - how can this be done? Do you mean combined? Are there workflows / examples / references for this?

- Line 599: "apply some process to the sample" sound strange and very general. Check the writing style throughout the whole paragraph.

- Line 612: "getting more scans done semiautomated is of interest" → check writing style.

- Line 620: explain or rephrase "vertical-adjusted multiple scan" - not easy to understand for new users

- Line 624 and following: I'd place this paragraph before the previous on batch scanning, as it has to do with maintenance / system not being available.

- Line 624: "Misconception" is strange here - I don't think any reader will have any idea about hardware failures and thus won't have any misconceptions.

- Line 626: "... stopped until the system is stable again" - explain what is meant by "stable" - this is not a full hardware failure. The first two sentences may be put into a more logical sequence.

- Line 627: Sentence starting with "However" is unrelated to the previous one(s). This should be placed directly after the first paragraph (line 609). Generally, consider re-structuring the paragraph 3.7 to follow a clearer line of thought.

-Figure 6: Make arrows smaller or different - text is difficult to read.

- Line 644: Drying a chameleon at ambient conditions may be fine, as the skin is tough and it will not change morphology, but please mention that this may destroy the sample and distort morphology (and certainly anatomy).

- Line 651 and following: "Since the sample was loaded at 45 degrees...": I cannot understand how a better voxel size can be achieved here by the tilt of the specimen. My experience has shown that aligning the specimen vertically and to the centre of the rotation stage will reduce the space needed during rotation, which in turn will allow a larger zoom / higher resolution, as the width of the sample on the detector is less. Please explain this reasoning.

- Line 656: "As the chameleon is a biological sample, no beam filtration was required" → This is not a universal guideline! More useful here would be to state that the combination of instrument, target, energy and the characteristics of the sample did not require a filter, or something like that. With 100kV I would certainly use a filter, probably even a Cu+Al (strongest filtration available).

- Lines 658 and following: What is the difference here between good penetration value but not high signal values? How did you evaluate this? And how/why does increase of current (independently of energy) help to improve this? Explain also why higher quality would have been possible at lower voltage and the relationship to penetration values. All these tips are potentially very helpful but they need a little more background explanation to those physical relationships and terms so that a user on a different instrument can follow the same reasoning on their machine, where settings may be completely different.

- Sections 3.8.2 - 3.8.4 could use a little more explanation for each step so that the user can actually understand the reasoning. Referring back to guidelines, as you did in 3.8.3.iii may be helpful.

- Shorten section 3.8.6 a little to remove narrative style. Example: the first few lines of the paragraph can be just shortened to "Without the need for physical sectioning, the head of the animal was scanned at a higher resolution of 30µ.....". Similarly, a more parsimonious style can be applied to the whole section. Reduce also explanations of figures (you have these already in the legends, so only a reference to figures are needed).

- Lines 730 and following: Other micro-CT models do not have problems at scanning at a 10µm and even higher resolution, so make clear that in your specific case you decided to use the available nano-CT, but that this is not a general guideline.

- Overall, the whole section on different resolutions could be shortened to a half-page paragraph, and images can be combined to a single one. This paragraph should just give the user an overview of the possibilities of different instruments / settings, but currently it would take about 3-4 pages in print, which I find a bit much.

4. Summary

- I suggest removing mentions/explanations of the "interesting biological findings" from the summary, biological investigations are not the scope of the paper.

Table 1:

- Stitching artefacts: the cause "sample too wide" is unclear. Stitching artefacts should only occur if the sample is too wide and you do an oversize scan (and these parts are stitched together), but then artefacts are a cause of software / algorithm problems.

- In Beam Hardening the part on"Edge of sample seems brighter...." in the "cause" column is actually an effect - the cause is only the part on the insufficient penetration of the sample.

- Histogram is shifted : The histogram is not mentioned/ explained anywhere in the text before, may merit a little more explanation.

- Scattering is not mentioned / explained in text. Solution to this may not be available in all scanners.

Level of Interest

Please indicate how interesting you found the manuscript: An article whose findings are important to those with closely related research interests

Quality of Written English

Please indicate the quality of language in the manuscript: Needs some language corrections before being published

Declaration of Competing Interests

Please complete a declaration of competing interests, considering the following questions:

- Have you in the past five years received reimbursements, fees, funding, or salary from an organisation that may in any way gain or lose financially from the publication of this manuscript, either now or in the future?
- Do you hold any stocks or shares in an organisation that may in any way gain or lose financially from the publication of this manuscript, either now or in the future?
- Do you hold or are you currently applying for any patents relating to the content of the manuscript?
- Have you received reimbursements, fees, funding, or salary from an organization that holds or has applied for patents relating to the content of the manuscript?
- Do you have any other financial competing interests?
- Do you have any non-financial competing interests in relation to this paper?

If you can answer no to all of the above, write 'I declare that I have no competing interests' below. If your reply is yes to any, please give details below.

I declare that I have no competing interests.

I agree to the open peer review policy of the journal. I understand that my name will be included on my report to the authors and, if the manuscript is accepted for publication, my named report including any attachments I upload will be posted on the website along with the authors' responses. I agree for my report to be made available under an Open Access Creative Commons CC-BY license (http://creativecommons.org/licenses/by/4.0/). I understand that any comments which I do not wish to be included in my named report can be included as confidential comments to the editors, which will not be published.

I agree to the open peer review policy of the journal
