# Supplementary material for: Laboratory x-ray micro-computed tomography: a user guideline for biological samples
Source: Gigascience. 2017 Apr 17;6(6):1–11. doi: 10.1093/gigascience/gix027 (PMC5449646; doi:10.1093/gigascience/gix027)
Supplement: Reviewer_2_Report_(Revision_1).pdf [file gix027_Reviewer_2_Report_(Revision_1).pdf]

Reviewer Report

Title: "Laboratory X-ray micro-computed tomography: a user guideline for biological samples"

Version: Revision 1 **Date:** 8/8/2016

Reviewer name: Sarah Faulwetter

Reviewer Comments to Author:

The paper "Laboratory X-ray micro-computed tomography: a user guideline for biological samples" has much improved, but there are still some revisions necessary. After these have been incorporated, I recommend the ms for publication.

The writing style has improved but the text still requires a careful linguistic revision. Grammatical errors are frequent (e.g. subject in third person singular, verb is not; subject in plural, verb is not; wrong punctuation, sentences without verbs, missing verbs......), as are linguistic / stylistic issues. Many of these may be a result of sloppiness, but overall the text will need a more scientific / parsimonous / less casual writing style. In some cases, the addition of paragraphs / sentences suggested by reviewers has lead to a strange text flow, as the previous and following sentences have not been adapted to reflect this text flow.

Overall, there are still some sections that are confusing to read and - in my opinion - not suitable to explain technical concepts and/or workflows to new users of micro-CT. Often, technical terms are used without explanation and then explained much later in the text, or paragraphs are not in the order of steps that need to be taken during the workflow. These sections need be thoroughly rephrased, sentences formulated very clearly, and paragraphs need to be brought into a consistent order. I believe that the help of a colleague would be very helpful here.

Some additional specific comments:

- Section 3.1.1, first sentences: In the response to the reviewers, a different new first paragraph is cited (" Section 3.1.1 has been changed accordingly and now reads: "However, some soft-tissue samples are preserved in a liquid and will damage if removed, therefore requiring scanning in the liquid as is. In these cases, staining increases the contrast of the specimen compared to the surrounding medium [4,

12, 11].""). There seems to be some confusion, please double check the text for the most up-to-date version.

- Section 3.1.1, p. 5, lines 10 and following: The issue with scanning samples in liquid (ethanol) is not their damage through manual handling but the potential desiccation when removed from their storage liquid, which causes irreversible changes to the morphology. Please consult with an invertebrate zoologist who is used to handle specimens preserved in liquids and correct this section. . In addition, the sentence "It is also possible to scan samples in liquid-filled..." does now not fit with the previous sections.

- Section 3.1.1, p. 5, lines 23 and following " The vertical mount method" is not explained.

- Page 8. line 20: figure 3 is referred to in the text before any reference to Fig2. Check if this is OK with the journal.

- Page 8. line 34: Although the reference to 3.1.6 about beam hardening has been inserted, the actual explanation on beam hardening has not been moved from the (old) section 3.1.5 to 3.1.6, thus there is currently no explanation on beam hardening in the ms.

- Page 8. line 35: Sentence "This penetration value" should go further up in the paragraph (somewhere after the first sentence) to ensure a better text flow.

- Page 8. line 38: Verbose sentence/paragraph, shorten.

- Page 9, guidelines: These are actually not guidelines, they don't stand alone (e.g. III makes no sense without II), Please merge them and rephrase them so that they can be used as independent guidelines. In addition, II and V are similar, can be merged)

- Page 12, lines 34 and following: A reference to "some scanners" has been inserted in the sentence, but now it reads as if some scanners have the option to remove the bright ring, not that the bright ring is only present in some scanners.

- Page 13, first paragraph: Can be shortened to make more clear and understanable. Rephrase to make clear that micro-CT does not have a built-in calibration, but data can be calibrated. Currently, the difference between medical scanners and micro-CT are unclear due to the style of writing.

- Page 14 line 2: replace "notepad" with "text editor" (notepad is a commercial product by Microsoft, not a general term for the type of software)

- Section 3.4. Please rewrite the whole paragraph until line 25. The differences between surface and volume rendering are still not clear, probably contain errors (Volume rendering does not involve iso-surface views), and remove the reference to CAD which is likely unknown to users. In addition, Blender is listed twice in the software section.

Section 3.5. The different options of thresholding are very confusing to read and difficult to understand. If possible, rephrase, in logical order, with clear explanations. If this is impossible without visual examples, please remove parts of the section. Currently, it is not helpful to a novel user.

- Page 15, line 12: Filtering the data is mentioned, but the explanation to data filtering is given below this paragraph. Add a reference (e.g. "see below") to make it more easy to understand.

- Page 15, line 33 and following: Paragraph is still unclear. It looks as if smoothing should be done before the segmenting (which you describe above)? Thus, move it before those steps in the description. Jumping back and forth between steps is confusing. Binarization is still unclear, too. why is it done here?

- Summary: Reference to the ostecyte structure is given, but not mentioned anymore in the text.

Level of Interest

Please indicate how interesting you found the manuscript: An article whose findings are important to those with closely related research interests

Quality of Written English

Please indicate the quality of language in the manuscript: Needs some language corrections before being published

Declaration of Competing Interests

Please complete a declaration of competing interests, considering the following questions:

- Have you in the past five years received reimbursements, fees, funding, or salary from an organisation that may in any way gain or lose financially from the publication of this manuscript, either now or in the future?
- Do you hold any stocks or shares in an organisation that may in any way gain or lose financially from the publication of this manuscript, either now or in the future?
- Do you hold or are you currently applying for any patents relating to the content of the manuscript?
- Have you received reimbursements, fees, funding, or salary from an organization that holds or has applied for patents relating to the content of the manuscript?
- Do you have any other financial competing interests?
- Do you have any non-financial competing interests in relation to this paper?

If you can answer no to all of the above, write 'I declare that I have no competing interests' below. If your reply is yes to any, please give details below.

I declare that I have no competing interests.

I agree to the open peer review policy of the journal. I understand that my name will be included on my report to the authors and, if the manuscript is accepted for publication, my named report including any attachments I upload will be posted on the website along with the authors' responses. I agree for my report to be made available under an Open Access Creative Commons CC-BY license (http://creativecommons.org/licenses/by/4.0/). I understand that any comments which I do not wish to be included in my named report can be included as confidential comments to the editors, which will not be published.

I agree to the open peer review policy of the journal
