# Supplementary material for: Laboratory x-ray micro-computed tomography: a user guideline for biological samples
Source: Gigascience. 2017 Apr 17;6(6):1–11. doi: 10.1093/gigascience/gix027 (PMC5449646; doi:10.1093/gigascience/gix027)
Supplement: Reviwer_1_Report_(Original_Submission).pdf [file gix027_Reviwer_1_Report_(Original_Submission).pdf]

Reviewer Report

Title: "Laboratory X-ray micro-computed tomography: a user guideline for biological samples"

Version: Original Submission **Date:** 6/30/2016

Reviewer name: Alexander Ziegler

Reviewer Comments to Author:

The article "Laboratory X-ray micro-computed tomography: a generalised approach for biological samples using a three-horned chameleon as example" by Anton du Plessis, Stephan Gerhard le Roux, and Anina Guelpa provides biologists interested in applying µCT to zoological specimens with valuable guidelines and technical information relevant for carrying out successful experiments. Furthermore, the authors have deposited a series of exemplary scans in a digital repository for download by the scientific community as well as the general public. The article will be of interest for all those zoologists that are planning to use µCT for their research purposes, but also contains some information that will be useful for more experienced µCT users. However, as detailed below, the article requires Major Revisions before it will be suitable for publication.

General comments

- You have chosen an inappropriate way to conduct this type of analysis using a large zoological wet sample. Every curator that knows what she or he is doing would not permit this kind of semi-destructive analysis using an ethanol-preserved specimen. In your particular case, the animal should either have been scanned in its original jar, in a closed plastic jar filled with a little ethanol or would have best been transferred to a plastic container completely filled with ethanol. Obviously, you then end up with the problem of even less tissue contrast and might - depending on what kind of tissue you need to visualize - have to stain the specimen, something that might be problematic using type material in particular. The approach you have chosen is OK if you just want to see mineralized parts (e.g. a vertebrate's skeleton), but in many, many zoological samples there are either no mineralized parts or they are of little interest. Your analysis therefore only applies to a very small range of potential zoological samples (mostly vertebrates), thus rendering your guideline inappropriate for the remaining zoological species. Try to modify your text according to this issue

- The article is too long for being a "how-to guide" - I therefore believe that the manuscript will profit significantly from a reduction of the text to about 60% of its current length. There is no specific paragraph or section that should be removed, but rather the authors should try to condense the text by removing overly generalized sentences and redundant information

- The article is at current a mix between a "Protocols" type of article (bullet points) and a "Methods" paper (running text). I would suggest to restructure most sections into running text were bullet points are currently used (e.g. 3.3.1. i. - ix. or 3.8.1. i.-vi., and especially 3.8.2.-3.8.4.). Usage of bullet points should be restricted to the five guidelines

- Given the complexity of the structure of the text (number of headings and subheadings), please consider adding a "Content" section - if the journal style permits - at the beginning of your article

- Language/grammar: please check your article carefully for spelling and grammar mistakes - I am not a native English speaker, but I spotted a number of mistakes throughout the text, figure legends, and tables

- Figures I: there are too many: remove 3 (not very informative), fuse 4 & 5, remove 6 (redundant with text/confusing), fuse 8 & 9, fuse 11 & 12 & 13

- Figures II: with your article you are targeting a readership that is accustomed to concise yet appealing figures. At present, most of your figures do not adhere to the basic standards in zoological articles and should therefore be amended: 1. Create proper figure plates with minimum spacing between individual images, 2. Use one type of standardized scale in all figures, 3. Label structures - what are we looking at? Consult with a vertebrate zoologist, 4. Crop images and remove all uninformative space (e.g. in Fig. 4 roughly 50% of each image are not needed), 5. Place image designators (e.g. (a), (b)) in the upper left corner of each image, 6. Restructure figure plates in such a manner that the final figure is broader than long (e.g. Fig. 10 should be composed of four images arranged in a rectangle rather than three images in a row)

- Please remove the line numbers in your original document file, the submission system is adding new numbers on top of your own line numbers

- I would recommend that, as a test prior to resubmission, you ask a zoologist that has never been in contact with a µCT scanner to read your manuscript to see if he or she can grasp what you want to communicate. I have the impression that the terminology in some parts might be too specific for a newcomer

Specific comments

- Keywords: some are redundant with the title - please remove

- Title is too long: please remove "using a three-horned chameleon as example". Your intention is to provide a general guideline, the example is irrelevant in this context

- Your line 18: "fascinating" - such terminology should be removed from the entire article. This is your personal opinion

- Your line 30: remove "medical", the abbreviation CT stands for "computed tomography", not "medical computed tomography"

- Your line 45: add "µCT"

- Your line 60: add reference for SRµCT, e.g. Betz et al. 2007 J Microscopy 227:51-71

- Your line 96: should read "usually 180° or 360°", I know of many colleagues that in order to reduce scan time no longer are scanning at 360°

- Your line 110: remove abbreviation "(CT)" from title

- Your line 122: remove URL

- Your line 127: guidelines - please think of another way to distinguish the five guidelines from the remainder of the text. Italics look inappropriate to me, use regular font

- Your line 136: the statements made here on staining specimens are too generalized, I recommend consulting literature currently not cited in your paper, e.g. Metscher 2009 BMC Physiology, Gignac et al. 2016 J Anat doi: 10.1111/joa.12449

- Your line 163: "cloth + skin" - this is indeed a very bad approach, please see my general comments. In order to circumvent the issues you are mentioning I have previously placed objects in ethanol-filled plastic jars and then have used very thin plastic balloons (the squeezing end of a plastic pipette) to position the animal at the center of the jar. Very tedious process, but it works. Although it usually means that the researcher could be experimenting around for an hour or more in order to find the best way to position his/her specimen in the container and then in the µCT scanner

- Your line 205 + 206: just use one scale for all values here: µm

- Your line 441: because of the points mentioned here, please also provide the raw data (i.e., the projections) of each scan together with the image stacks. This aspect has been discussed in Lenihan et al. 2014 GigaScience 3:6

- Your line 468: should read "0 to 255"

- Your line 469: should read "0 to 65,535"

- Your lines 504-508: this selection appears random and does not specify whether the software is free or commercial. Please look for articles that provide such information, e.g. Walter et al. 2010 Nat Meth Suppl 7:S26-S41 or better more recent papers

- Your line 604: there are also systems on the marketthat are almost maintenance-free, in particular those with a sealed X-ray source

- Your line 696: remove "(due to its use for catching prey)"

- Your line 717: why would it be "more costly" if the scans are for free?

- Your line 735: remove the entire sentence starting with "It is similar to most..." - you are addressing newcomers to µCT, so how can they "well know" benchtop µCT systems?

- Your line 759: remove sentence starting with "Data sets are included..." - redundant content and inappropriate terminology

- Your line 772: this is text that should be written by a zoologist - either modify and correct it or remove it

- Your line 755: remove second half of sentence - speculative and exaggerated

- Table 1: it would be nice to have visual examples for all artifacts/issues listed here - see , e.g. Fernandez et al. 2014 PLoS ONE 9:96617

- Deposited data: please rename your individual stacks in a consistent manner, e.g. Full body stack 75 µm, Head stack 30 µm etc. Add the specimen ID for future reference

- Figures: please see my comments in the general comments section above

- Figure 1: improve by adding more labeling, e.g. sample, radiation (as opposed to attenuated radiation) and by centering/improving the labels in the figure

- Figure 2: (b) is shown from an oblique angle - inappropriate to illustrate what you want to convey; a + b vs. c are extremely different images - the context should be made clearer by showing step-wise 1. mounting of the animal and then 2. positioning it in the scanner

- Figure 3: remove

- Figure 4: these images should be taken at the same level for direct comparison

- Figure 6: remove

- Figure 9: remove the red crossmarks from the image - artificial structure

- Figure 10: (a) why is the head surface-rendered in green when it is grey in Fig. 8? Check all figures for consistency (same applies to skeletal parts...yellow, grey). Remove the cropping box from the image (c) - you might then need to choose another angle to illustrate what you want to show

- Figure 11: remove "35%" - what does this mean?

- Figure 12: orient images in same way - why is (b) shown oblique?

- Figure 13: as in Fig. 12 - why are they oriented differently? Also, remove the labeling "Defect volume" - an osteocyte is not a defect

I hope that these suggestions will be helpful to conduct the necessary corrections.

Kind regards

Alexander Ziegler

Level of Interest

Please indicate how interesting you found the manuscript: An article whose findings are important to those with closely related research interests

Quality of Written English

Please indicate the quality of language in the manuscript: Needs some language corrections before being published

Declaration of Competing Interests

Please complete a declaration of competing interests, considering the following questions:

- Have you in the past five years received reimbursements, fees, funding, or salary from an organisation that may in any way gain or lose financially from the publication of this manuscript, either now or in the future?
- Do you hold any stocks or shares in an organisation that may in any way gain or lose financially from the publication of this manuscript, either now or in the future?
- Do you hold or are you currently applying for any patents relating to the content of the manuscript?
- Have you received reimbursements, fees, funding, or salary from an organization that holds or has applied for patents relating to the content of the manuscript?
- Do you have any other financial competing interests?
- Do you have any non-financial competing interests in relation to this paper?

If you can answer no to all of the above, write 'I declare that I have no competing interests' below. If your reply is yes to any, please give details below.

I declare that I have no competing interests.

I agree to the open peer review policy of the journal. I understand that my name will be included on my report to the authors and, if the manuscript is accepted for publication, my named report including any attachments I upload will be posted on the website along with the authors' responses. I agree for my report to be made available under an Open Access Creative Commons CC-BY license (http://creativecommons.org/licenses/by/4.0/). I understand that any comments which I do not wish to be included in my named report can be included as confidential comments to the editors, which will not be published.

I agree to the open peer review policy of the journal
